# Evolutionary innovations in Antarctic brittle stars linked to glacial refugia

**DOI:** 10.1002/ece3.8376

**Published:** 2021-11-29

**Authors:** Sally C. Y. Lau, Jan M. Strugnell, Chester J. Sands, Catarina N. S. Silva, Nerida G. Wilson

**Affiliations:** ^1^ Centre for Sustainable Tropical Fisheries and Aquaculture and College of Science and Engineering James Cook University Townsville Qld Australia; ^2^ Department of Ecology, Environment and Evolution School of Life Sciences La Trobe University Melbourne Vic Australia; ^3^ Securing Antarctica's Environmental Future James Cook University Townsville Qld Australia; ^4^ British Antarctic Survey Natural Environment Research Council Cambridge UK; ^5^ Collections & Research Western Australian Museum Welshpool WA Australia; ^6^ School of Biological Sciences University of Western Australia Perth WA Australia; ^7^ Securing Antarctica's Environmental Future Western Australian Museum Welshpool WA Australia

**Keywords:** contrasting life histories, evolutionary innovation, glacial refugia, morphological innovation, population genetics

## Abstract

The drivers behind evolutionary innovations such as contrasting life histories and morphological change are central questions of evolutionary biology. However, the environmental and ecological contexts linked to evolutionary innovations are generally unclear. During the Pleistocene glacial cycles, grounded ice sheets expanded across the Southern Ocean continental shelf. Limited ice‐free areas remained, and fauna were isolated from other refugial populations. Survival in Southern Ocean refugia could present opportunities for ecological adaptation and evolutionary innovation. Here, we reconstructed the phylogeographic patterns of circum‐Antarctic brittle stars *Ophionotus victoriae* and *O*. *hexactis* with contrasting life histories (broadcasting vs brooding) and morphology (5 vs 6 arms). We examined the evolutionary relationship between the two species using cytochrome c oxidase subunit I (COI) data. COI data suggested that *O*. *victoriae* is a single species (rather than a species complex) and is closely related to *O*. *hexactis* (a separate species). Since their recent divergence in the mid‐Pleistocene, *O*. *victoriae* and *O*. *hexactis* likely persisted differently throughout glacial maxima, in deep‐sea and Antarctic island refugia, respectively. Genetic connectivity, within and between the Antarctic continental shelf and islands, was also observed and could be linked to the Antarctic Circumpolar Current and local oceanographic regimes. Signatures of a probable seascape corridor linking connectivity between the Scotia Sea and Prydz Bay are also highlighted. We suggest that survival in Antarctic island refugia was associated with increase in arm number and a switch from broadcast spawning to brooding in *O*. *hexactis*, and propose that it could be linked to environmental changes (such as salinity) associated with intensified interglacial‐glacial cycles.

## Graphical abstract

The drivers behind evolutionary innovations such as contrasting life histories and morphological change are central questions of evolutionary biology, but the contexts linked to innovations are generally unclear. We explored the phylogeographic patterns of the circum‐Antarctic brittle stars Ophionotus victoriae and *O*. *hexactis* with contrasting life histories (broadcasting vs brooding) and morphology (5 vs 6 arms). We propose that survival in refugia was associated with increase in arm number and a switch from broadcast spawning to brooding in *O*. *hexactis*, and suggest that innovations could be linked to environmental changes associated with intensified interglacial‐glacial cycles.

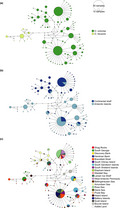

## 1 INTRODUCTION

In marine invertebrates, early life‐history strategy influences species dispersal potential, and this, in turn, can shape population‐level gene flow and long‐term evolutionary histories (Hart & Marko, 2010). Although pelagic development with planktotrophic (feeding) larvae has been suggested as the ancestral mode in most marine taxa (Strathamnn, 1985), non‐pelagic direct development (brooding) has been linked to evolution under increased offspring provisioning (Wray & Raff, 1991). Brooding is commonly observed as an evolutionary transition from broadcast spawning across broad lineages (Strathamnn, 1985), but contrasting life histories (brooding and broadcast spawning) are also often reported between congeneric species in speciose clades, including echinoderms (Collin & Moran, 2018). The main drivers behind contrasting life histories are often unknown, but in some reported cases, transitions from pelagic to direct development can be linked to ecological and/or environmental changes (Boissin et al., 2011). Contrasting life histories have also been observed in congeneric species in the Antarctic and Southern Ocean (e.g., Jossart et al., 2019). If investigated, they could offer insights into variation in evolutionary processes, constrained within similar environments.

Throughout the Plio‐Pleistocene (5,000,000–12,000 years ago), glacial cycles driven by climatic oscillations were significant in structuring past evolutionary histories in the terrestrial and marine realm (Hewitt, 2004; Maggs et al., 2008; Provan & Bennett, 2008). In the Northern Hemisphere, in response to ice sheet expansion and subsequent erosion of habitats, some Arctic and temperate taxa migrated to warmer, lower latitude ice‐free areas for refuge, with some persisting in small‐scale ice‐free refugia (Maggs et al., 2008; Provan & Bennett, 2008). In the Southern Hemisphere, the continental‐based Antarctic ice sheet also expanded and eroded most of the continental shelf seafloor habitats in the Southern Ocean (Clarke & Crame, 1992; Thatje et al., 2005). However, migration to lower latitudes appears improbable for Southern Ocean fauna because of the strong Antarctic Circumpolar Current (ACC) with various frontal boundaries surrounding the Antarctic continent (Rintoul et al., 2001; Thatje et al., 2005). These ocean barriers have been suggested to play an important role in isolating Southern Ocean taxa from other ocean basins since the mid‐Miocene (~14 million years ago) (Crame, 2018). Throughout the Pleistocene glacial cycles, the Southern Ocean benthic fauna are hypothesized to have either persisted in limited, isolated ice‐free areas on the Antarctic continental shelf, or migrated to, and survived in, the surrounding deep sea or around Antarctic islands off the shelf (Allcock & Strugnell, 2012; Convey et al., 2009; Thatje et al., 2005).

Persistence in isolated Southern Ocean refugia has been suggested to favor non‐pelagic development due to higher chances of surviving glacial periods, when the Southern Ocean experienced limited habitat availability and low primary productivity linked to permanent ice cover (Convey et al., 2009; Pearse et al., 2009; Poulin et al., 2002; Thatje et al., 2005). However, it has been suggested that pelagic development was also a successful strategy in persisting throughout glacial cycles in the Southern Ocean, and selection likely acted differently on different developmental modes throughout glacial periods (Lau et al., 2020). Survival in allopatric refugia would present unique challenges for fauna to persist within each isolated environment, as well as providing opportunities to drive evolutionary innovations and phenotypic changes (e.g., morphological variation and reproductive specialization) between isolated populations. Furthermore, the Plio‐Pleistocene glacial period was also characterized by several, but rare, “warm” climate periods (between 1 and 4°C warmer than the Holocene) (Noble et al., 2020). The environmental fluctuation between extreme conditions (glacial maxima and warm interglacial) could also promote niche diversity and ecological diversification (Clarke & Crame, 1992).

Evolutionary innovations can be represented by new traits, which often opens new ecological niches where further evolutionary changes can unfold (Wagner, 2011). New traits can include trait expression and/or novel function, and these can be a broad range of behavioral, physiological, and morphological characteristics (Love, 2003; Moczek et al., 2011), whereas key innovations are a small subset of these that are invoked as underpinning evolutionary radiations. Events such as increased diversification rate and utilization of new and/or altered habitats have been suggested to be associated with evolutionary innovations (Dumont et al., 2012; Wilson et al., 2013). There is evidence indicating Southern Ocean glacial refugia could have provided opportunities for evolutionary innovations. First, persistence in Southern Ocean refugia has been suggested to have promoted allopatric diversification and subsequent speciation (i.e., the “species pumps” and the “Antarctic biodiversity pump” hypotheses) (Clarke & Crame, 1989; Crame, 1997; Willis & Whittaker, 2000). Cryptic speciation and/or lineage diversification linked to glacial cycles and/or glacial refugia survival has been suggested for many benthic taxa (Allcock et al., 2011; Baird et al., 2011; González‐Wevar et al., 2019; Strugnell et al., 2012; Wilson et al., 2009). Second, Southern Ocean benthic taxa experienced repeated migrations to new and/or altered habitats as ice sheets expanded and contracted throughout glacial‐interglacial cycles (see Lau et al., 2020 for a review). Lastly, evolutionary innovations with novel biological changes linked to survival in Southern Ocean glacial refugia have also been observed. Cryptic species within the sea slug *D*. *kerguelenensis* species complex survived in allopatric refugia on the Antarctic continental shelf. Over glacial cycles, *D*. *kerguelenensis* underwent lineage diversification across allopatric refugia, as well as developing distinct metabolites as an adaptation to unique predation pressure within local refugial environments (Wilson et al., 2013). Nonetheless, limited examples have reported the association between evolutionary innovation and survival within glacial refugia, or past interglacial periods, to date in the global marine realm.

The brittle star genus *Ophionotus* Bell, 1902 is distributed widely throughout the Southern Ocean including the Antarctic continental shelf, deep sea, and islands within the Antarctic Polar Front (APF). *Ophionotus* is comprised of three species, including *O*. *victoriae* Bell, 1902, *O*. *hexactis* E. A. Smith, 1876, and *O*. *taylori* McKnight, 1967. *Ophionotus victoriae* is characterized by five arms, pelagic planktotrophic larvae (Grange et al., 2004), and a widespread distribution across the Southern Ocean at depths ranging from shallow water to 1750 m (this study; specimen IDs: WAMZ88591—WAMZ88594). *Ophionotus hexactis* is characterized by six arms, brooding larvae, and is mainly distributed around Antarctic islands near the APF at depths ranging from shallow water to 459 m (GBIF.org, 2019; McClintock, 1994; Turner & Dearborn, 1979). However, *O*. *hexactis* has also been collected from the Antarctic Peninsula on the Antarctic continental shelf (Hugall et al., 2016). *Ophionotus taylori* is characterized by five arms, and its occurrence has never been reported since the type specimen was collected from Cape Hallett, Ross Sea. Compared to *O*. *victoriae*, *O*. *taylori* possesses notably coarser and thicker scales, with other taxonomic features (including arm shape, arm spines, arm plates, oral shields) different in size and shape relative to *O*. *victoriae* (McKnight, 1967).

Previous studies employing genetic (mitochondrial markers 16S rRNA and COI) and genomic (2b‐restriction site‐associated DNA (2b‐RAD) sequencing) methods have suggested high genetic differentiation between distant sampling locations within *O*. *victoriae*, and it has been suggested to be comprised of multiple cryptic species (Galaska et al., 2017a; Hunter & Halanych, 2010). However, prior sampling efforts have been focused in some locations in West Antarctica. Samples from East Antarctica and some Antarctic islands (Shag Rocks, South Georgia, Heard Island, Scott Island, Balleny Islands) are not yet represented in genetic studies for this taxon. Therefore, the previous interpretation of cryptic species within *O*. *victoriae* may have been influenced by the limited and disjunct sampling of a widely distributed species, reflecting artifacts caused by isolation‐by‐distance. Lack of comprehensive sampling is common in Southern Ocean ecological studies. Biological samples from East Antarctica and Antarctic islands are incredibly rare, since these areas are difficult to access and are distant from most national research stations (Griffiths et al., 2014).

Given that *O*. *victoriae* and *O*. *hexactis* are characterized by overlapping distribution and wide‐ranging depths in the Southern Ocean, increased sampling effort may provide a more thorough understanding of their past demographic histories. The morphology and early life history of *O*. *hexactis* is striking within echinoderms, as this phylum is evolutionarily primed to pentameral symmetry (i.e., 5 arms in brittle stars) (Rozhnov, 2012) and pelagic development with planktotrophic larvae (Gillespie & McClintock, 2007). *Ophionotus victoriae* and *O*. *hexactis* are currently recognized as separate species (WoRMS Editorial Board, 2021), distinguished by the number of arms (5 versus 6) and reproductive features (oviparous vs viviparous) (Bell, 1902; Smith, 1876). However, a single five‐armed *O*. *hexactis* specimen from South Georgia has also been reported to exhibit brooding behavior with six‐arm juveniles (Mortensen, 1936), indicating there could be rare biological exceptions. Recent COI and exon capture data also suggest a close genetic distance between *O*. *victoriae* and *O*. *hexactis* (Galaska et al., 2017a; Hugall et al., 2016), despite their obvious morphological differences. Given the apparent close phylogenetic relationship and highly differentiated morphological variation between *O*. *victoriae* and *O*. *hexactis*, comparing their past demographic histories could provide insight into species history.

Critically, in this study, we have incorporated new *O*. *victoriae* samples from rarely sampled regions including East Antarctica (Prydz Bay, Davis Sea, Adélie Land) and Antarctic islands (South Georgia, Shag Rocks, Discovery Bank, Herdman Bank, Balleny Islands, Scott Islands, Heard Island) in order to holistically examine evolutionary processes across the Southern Ocean, along a geographical and circumpolar cline, in a species with a circum‐Antarctic distribution. We also incorporated new *O*. *victoriae* samples from previously surveyed areas (Bouvet Island, Bransfield Strait, Discovery Bank, Elephant Island, Shetland Islands, South Sandwich Islands, Larsen Ice Shelf, Ross Sea, and Weddell Sea) to increase sample robustness. We used COI sequence data from samples collected from an expanded distribution to determine (a) whether *O*. *victoriae* contains cryptic species, (b) how genetic structure is characterized in *O*. *victoriae* and *O*. *hexactis*, (c) if there is genetic evidence indicating how *O*. *victoriae* and *O*. *hexactis* have survived glacial cycles, and finally, (d) whether the divergence between *O*. *victoriae* and *O*. *hexactis* can be linked to isolation‐by‐environment and present‐day conditions. We used these analyses to investigate the ecological and evolutionary context that could explain the life‐history and morphological differences between *O*. *victoriae* and *O*. *hexactis*.

## 2 METHODS

### 2.1 Sample collection

**FIGURE 1:**
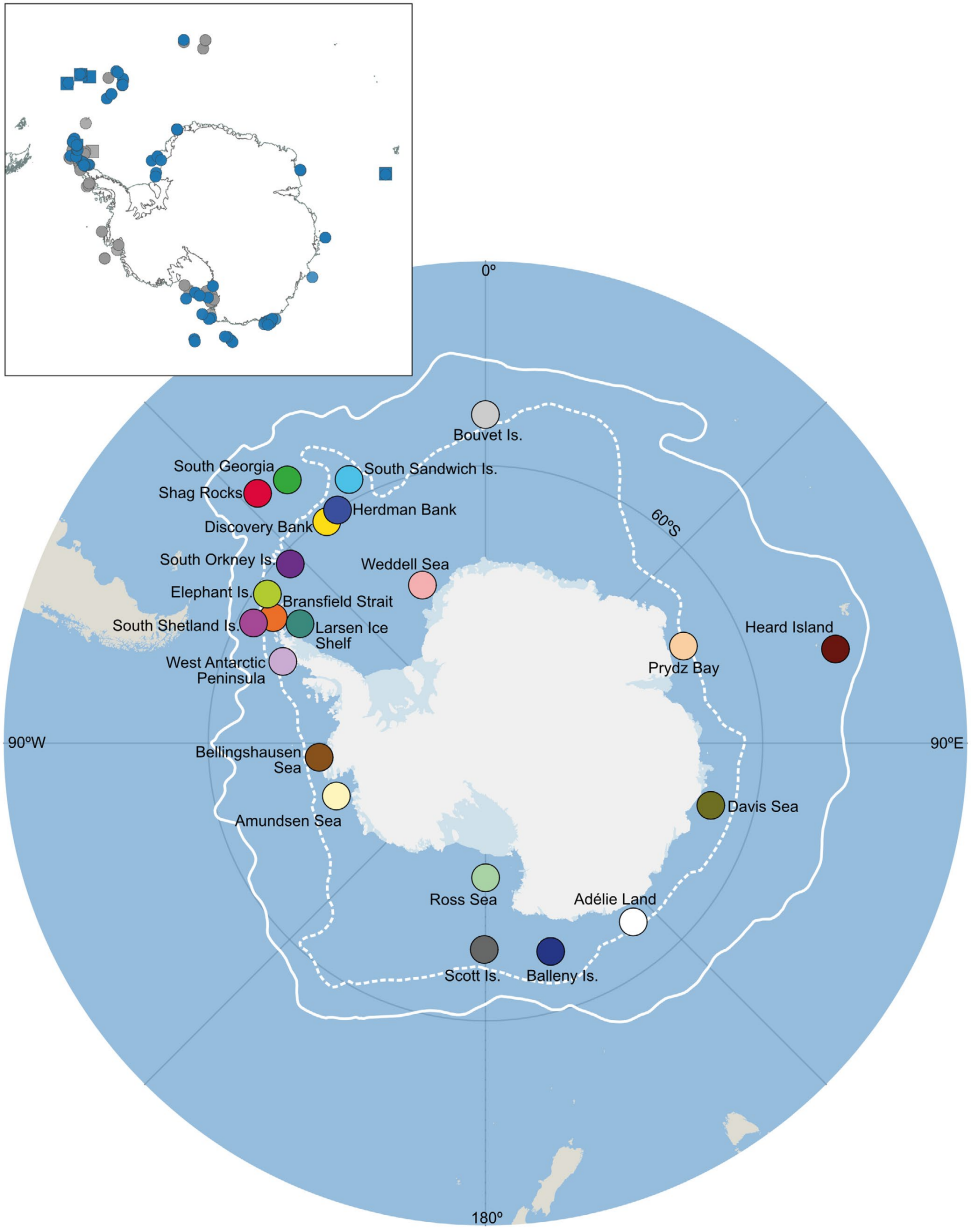
Map of Southern Ocean with sampling locations of *Ophionotus* *victoriae* and *O. hexactis* defined for population genetic analyses. White lines = Antarctic Polar Front (APF) (solid) and southern boundary of the Antarctic Circumpolar Current (dashed). Top left map indicates the distribution of individual samples, blue = sequences generated in this study, gray = GenBank accessions, circles = *O*. *victoriae*, square = *O. hexactis*

This newly generated dataset was sequenced from individuals of *Ophionotus victoriae* (*n* = 443) and *O*. *hexactis* (*n* = 72) deposited at Western Australian Museum (WAM), Muséum National d'Histoire Naturelle (MNHN‐IE), Museum Victoria (MV), Scripps Institution of Oceanography (SIO‐BIC), and the National Institute of Water and Atmospheric Research (NIWA). All newly sequenced *O*. *victoriae* and *O*. *hexactis* samples were preserved in 50–100% ethanol and were identified through their readily distinguishable (and diagnostic) pentamerous and hexamerous arm symmetry, respectively. We note that it is plausible that *O*. *hexactis* with 5 arms exist in the dataset. Partial COI sequences of *O*. *victoriae* (*n* = 419) and *O*. *hexactis* (*n* = 1) from previous studies were also included in the data analysis (Galaska et al., 2017a; Hunter & Halanych, 2010) (Appendix S1 for GenBank Accession numbers). All brittle star samples (*n* = 935) investigated in this study were collected between the years 2004–2019, from depths of 34–1750 m during expeditions in the Southern Ocean (Figure 1, details of sampling information and GenBank Accession numbers are presented in Appendix S1).

### 2.2 Molecular sequencing

Genomic DNA of the collected samples was extracted using DNeasy Blood and Tissue Kit (Qiagen), following the manufacturer's protocol. Partial COI sequences were then amplified using genus‐specific primers Op4f (5′‐TAGTGACTGCCCATGCCTTC‐3′) and COI_op3r (5′‐TTTTTCGATCAGTGAGGAGC‐3′) developed by Jose Carvajal (SIO/WAM). Each 25 μl PCR contains 5.0 μl of 5× MyTaq PCR buffer (Bioline), 0.2 μl of 5 U/μl MyTaq DNA polymerase (Bioline), 0.8 μl of each 10 μM primer (forward and reverse), 1.5 μl of template genomic DNA (5 – 20 ng/μl), and 16.8 μl of water. PCR cycling profile conditions were as follows: initial denaturation at 95°C for 3 min, 8 cycles of 95°C for 30 s, 52°C for 30 s with a touchdown which the annealing temperature was reduced by 1°C at every cycle, and 72°C for 45 s, followed by 38 cycles of 95°C for 30 s, 48°C for 30 s, and 72°C for 45 s, and a final extension step at 72°C for 5 min. PCR products were then sent to Australian Genome Research Facility (AGRF) (Perth and Brisbane, Australia) for purification and sequencing in both directions.

While PCR amplification with Op4f and COI_op3r was successful in most specimens, two *O*. *victoriae* individuals collected from South Georgia yielded poorly amplified but detectable PCR products (ID: SIO‐BIC E6408 and SIO‐BIC E6420). Therefore, based on the assembled COI alignments of *O*. *victoriae* and *O*. *hexactis* collected from their overlapped distributional regions (South Georgia, Bransfield Strait and Heard Island) (see Appendix S2 for photos of specimens), two sets of internal primers were designed for nested PCR to target the same positions as those Op4f and COI_op3r would target in *Ophionotus* spp. The two internal primer pairs designed by this study include the following: oph_head‐F (5′‐TTGGGGGATTTGGAAACTGG‐3′)/‐R (5′‐AGACCAAACAAATAAAGGAGTTCGG‐3′) and oph_tail‐F (5′‐CCCCGGATATGGCATTTCCT‐3′)/‐R (5′‐TTGCCCCTGCTAATACTGGT‐3′). All assembled COI sequences were aligned using the Multiple Alignment using Fast Fourier Transform (MAFFT) (Katoh & Standley, 2013) plug‐in Geneious v10.2.4 (https://www.geneious.com), using default values and trimmed to 434 base pairs (bp).

### 2.3 Network reconstruction and population genetics

A median joining (MJ) haplotype network (Bandelt et al., 1999) with epsilon = 0 was constructed using *PopART* (Leigh & Bryant, 2015) to visualize the relationships between individual samples within *O*. *victoriae* and *O*. *hexactis*, as well as relationships between species. We have also explored a MJ network with epsilon = 10 to widen the search for unobserved sequences (i.e., hypothesized haplotypes). However, the algorithm produced too many unnecessary hypothesized haplotypes and eventually broke down. Therefore, the MJ network with epsilon = 0 has achieved a sufficient level of exploration in reconstructing all possible shortest and least complex phylogenetic trees for discussion. A TCS haplotype network with a default connection limit of 95% was also constructed using *PopART* to evaluate consistency of results across different network assumptions. For population genetic analyses, COI sequences were first grouped by species and then further divided into sample localities defined in Table 1 (Figure 1). All the sampled locations are within the APF. Sample locations on the Antarctic continental shelf were considered “continental shelf,” and islands located off the Antarctic continental shelf were considered as “Antarctic islands.” Population genetic statistics including genetic diversity (nucleotide and haplotype), number of polymorphic sites, and average number of nucleotide difference were calculated for each sampling locality using Arlequin v3.5 (Excoffier & Lischer, 2010). Pairwise F_ST_ and subsequent analysis of molecular variance (AMOVA) based on 1000 permutations was also calculated in Arlequin to examine genetic differentiation between *O*. *victoriae* and *O. hexactis*, as well as between sampling localities within *O*. *victoriae* and *O*. *hexactis*. The number of private haplotypes at each locality was calculated using Fabox v1.5 (Villesen, 2007).

**TABLE 1:** Population statistics of *Ophionotus victoriae* and *O*. *hexactis* based on COI data

Species	Locality		*n*	Hd	Hp	*h*	P(Hp)	*S*	*π*	Π	Tajima's *D*	Fu's *F_S_*	Mismatch distribution
*O. victoriae*	All		862	0.887	102	165	0.62	60	0.01801	5.44006	−0.64100	−23.83934*	Unimodal
	Antarctic islands		445	0.857	74	103	0.72	50	0.00994	3.01191	−1.44457*	−22.42260*	Unimodal
		South Georgia	2										
		Discovery Bank	20	0.589	7	8	0.88	10	0.00308	1.33684	−1.41266	−1.083	Multimodal
		Herdman Bank	20	0.811	5	8	0.63	8	0.00589	2.26842	−0.02138	11.20541	Unimodal
		Bransfield Strait	97	0.835	18	28	0.64	37	0.01106	4.31508	−1.06489	−1.07556	Multimodal
		South Orkney Island	1										
		South Sandwich Islands	104	0.768	25	30	0.83	29	0.00780	3.01680	−1.23021	−4.71546	Unimodal
		Shetland Islands	63	0.919	10	24	0.42	30	0.01577	6.29237	0.12392	−0.07908	Unimodal
		Elephant Island	17	0.890	5	10	0.50	8	0.00552	2.39706	0.04589	−3.16436	Unimodal
		Heard Island	1										
		Balleny Islands	48	0.441	9	11	0.82	19	0.00229	0.99366	−2.55063	−4.10089*	Multimodal
		Scott Island	25	0.410	7	9	0.78	3	0.00144	0.44000	−0.86557	−0.5395	Unimodal
		Bouvet Island	47	0.732	9	13	0.69	18	0.00559	2.42738	−1.28531	−3.75045	Multimodal
	Antarctic continental shelf		417	0.838	36	71	0.51	54	0.01832	6.57682	−0.09963	−11.08477	Multimodal
		Weddell Sea	38	0.853	5	13	0.38	25	0.01696	7.34424	0.7998	3.40484	Multimodal
		Larsen Ice Shelf	106	0.915	9	27	0.33	33	0.02003	8.67260	1.14744	−0.72463	Multimodal
		West Antarctic Peninsula	56	0.716	6	13	0.46	21	0.01314	5.70455	0.78143	0.83817	Multimodal
		Bellingshausen Sea	9	0.944	5	7	0.71	17	0.01140	4.83333	−1.10641	−1.31053	Multimodal
		Amundsen Sea	67	0.903	9	18	0.50	24	0.00690	2.99367	−1.26273	−5.7985	Multimodal
		Ross Sea	114	0.478	11	15	0.73	30	0.00638	2.30255	−1.01988	0.96876	Multimodal
		Adélie Land	17	0.787	7	9	0.78	27	0.01792	7.77941	−0.1049	0.73887	Multimodal
		Davis Sea	4	0.833	2	3	0.67	15	0.01728	7.50000	−0.84729	2.14949	Multimodal
		Prydz Bay	6	0.600	2	3	0.67	2	0.00154	0.66667	−1.13197	−0.85842	Multimodal
*O. hexactis*	All		73	0.715	7	13	0.54	27	0.01276	5.49500	−0.0248	1.41432	Multimodal
	Antarctic islands		72	0.707	6	12	0.50	26	0.01271	5.51598	0.09727	2.02924	Multimodal
		South Georgia	40	0.535	3	7	0.43	6	0.00269	1.16923	−0.45659	−1.74652	Multimodal
		Shag Rocks	10	0.200	1	2	0.50	1	0.00046	0.20000	−1.11173	−0.33931	Multimodal
		Bransfield Strait	12	0.295	2	3	0.67	20	0.00798	3.46154	−1.98015*	4.30689	Multimodal
		Heard Island	10	0.200	1	2	0.50	3	0.00138	0.60000	−1.56222	1.22453	Multimodal
	Antarctic continental shelf												
		Larsen Ice Shelf	1										

Note

Antarctic islands are referred to the islands south of the Antarctic Polar Front. Hd = haplotype diversity, Hp = number of private haplotypes, *h* = number of haplotypes, P(Hp) = proportion of private haplotypes, *S* = number of polymorphic sites, *π* = nucleotide diversity, Π = average number of nucleotide difference.

*Statistical significance at *p* < .05.

We have also examined the species boundaries and relationships between *O*. *victoriae* and *O*. *hexactis* using phylogenetic tree reconstructions and species delimitation methods. Our study only contains one genetic marker (mitochondrial COI gene), which may not contain sufficient information to diagnose species status (DeSalle et al., 2005). However, as species delimitation using COI may still be of interest to the wider research community (DeSalle & Goldstein, 2019) and is also useful in for providing hypotheses for future studies employing nuclear data, we have presented the methods and results of species delimitation in Appendix S3. Throughout this study, we view *O. victoriae* and *O*. *hexactis* as two taxonomically recognized separate species.

### 2.4 Demographic histories

The past demographic histories of *O*. *victoriae* and *O*. *hexactis* (analyzed separately) were investigated via neutrality tests (Tajima's *D* and Fu's *F_S_*), mismatch distributions (pairwise differences distributions), and past population size changes (Bayesian Skyline Plots; BSP). Tajima's *D* and Fu's *F_S_* were calculated in Arlequin to examine whether data deviated from a neutral evolution model, with significance tested by 1000 permutations. Distributions of pairwise differences to estimate parameters of demographic expansion (mismatch distribution) were calculated using the R package “adegenet” (Jombart & Ahmed, 2011) and “pegas” (Paradis, 2010) in R v 3.3.3. Past changes in effective population size over time in *O*. *victoriae* and *O*. *hexactis* were also estimated using BSP in BEAST v2.5.0 (Bouckaert et al., 2019). BEAST was performed under the substitution model of TN+F+I+G4 (identified via Bayesian information criterion (BIC) using ModelFinder on the IQ‐TREE web server (Kalyaanamoorthy et al., 2017)), uncorrelated lognormal relaxed clock and using a constant coalescent constant population tree prior (Michonneau, 2016). A Markov chain Monte Carlo (MCMC) analysis was run for 500 million (*O*. *victoriae*) and 200 million (*O*. *hexactis*) generations sampled at every 5000 generations. A longer MCMC was required for *O*. *victoriae* as the length of chain is correlated to the number of individual sequences included (*n* = 826 in *O*. *victoriae*) (Drummond et al., 2007). Tracer v1.7.1 (Rambaut et al., 2018) was used to inspect convergence based on trace plots and effective sample size (ESS; >200). A substitution rate of 2.48% per million years (2.48 × 10^−8^ per lineage per year) was employed following other analyses of COI data for ophiuroids (Naughton et al., 2014; Sands et al., 2015).

### 2.5 Spatial genetic variation within *O. victoriae*

To explore how genetic variation is spatially structured in the Southern Ocean, spatial pattern detection analysis was performed within *O*. *victoriae*. Spatial pattern detection analysis was not performed for *O*. *hexactis* as the variation in sample coordinates was limited, leading to a singular matrix not suitable for a multivariate correlation analysis. For *O*. *victoriae*, a matrix of genetic *p*‐distance between individual COI sequences was first calculated based on the substitution model of TN+F+I+G4 using the “APE” package in R (Paradis et al., 2004). The “mgQuick” function of the R package “MEMGENE” (Galpern et al., 2014) was used to extract the spatial components of genetic variation attributed to isolation‐by‐distance (i.e., Euclidean distances; straight linear geographical distance) between samples. “mgQuick” uses Moran's eigenvector maps (MEM) to create orthogonal eigenvectors from Euclidean distances and then uses redundancy analysis (RDA) to quantify the proportion of genetic variation explained by each eigenvector (i.e., MEMGENE variables). MEMGENE variables are ranked by the amount of genetic variation explained by Euclidean distances from the most to least, and the first two MEMGENE variables typically outline most of the detected spatial genetic patterns (Galpern et al., 2014). To visualize each MEMGENE variable, samples are first mapped based on their geographical locations. Each sample's predicted eigenvector score is then overlaid on the map to visualize the spatial component of genetic similarity or dissimilarity among individuals, thus highlighting genetic clusters linked to isolation‐by‐distance.

### 2.6 Isolation‐by‐environment between species

Isolation‐by‐environment was also investigated in MEMGENE to detect whether environmental heterogeneity (in the form of resistance surfaces) may also explain the genetic variation between *O*. *victoriae* and *O*. *hexactis*. Any significant association to environmental variables detected by isolation‐by‐environment may reflect nonrandom mating linked to environmental differences and/or local adaptation linked to selection (Sexton et al., 2013). Although including samples from both species in an IBE analysis assumes the intrinsic reproductive isolation between *O*. *victoriae* and *O*. *hexactis* is incomplete, exploring how the genetic variation between closely related species is associated with an heterogeneous environment could offer insights into how the environment could influence species differentiation (e.g., Saenz‐Agudelo et al., 2015). The environmental parameters considered in this analysis included sea surface temperature, seafloor temperature, sea surface salinity, seafloor salinity, surface current velocity (as a variable of physical transport patterns), and geological bathymetry. The resistance surfaces representing each environmental parameter were produced from the temperature and salinity point datasets from World Ocean Atlas 2018 (Locarnini et al., 2018; Zweng et al., 2018), as well as extracted from the raster layers of Southern Ocean State Estimate (SOSE) mean surface current speed (cell resolution = 16 km) (Mazloff et al., 2010) and ETOPO1/IBCSO/RAMP2 hillshades and elevation (surface and seafloor) model (cell resolution = 1 km) (Amante & Eakins, 2009) from Quantarctica (Matsuoka et al., 2018) using QGIS (QGIS Development Team, 2019). Southern Ocean temperature and salinity data were reproduced from global point datasets (climatological means) at 1°C spatial resolution of annual average per decade between 1955 and 2010, with temperature and salinity data available at 102 depth levels ranging from 0 to 5500 m for each point. Surface temperature and salinity data were estimated from a value at 0 m water depth for each point, whereas seafloor temperature and salinity data were estimated based on the data value at the depth interval closest to the maximum depth available for each point. All extracted temperature and salinity data were transformed to single raster layers via triangular interpolation method in QGIS (Interpolation plug‐in).

As the raster layer of surface current speed was pre‐defined with a cell resolution of 16 km in Mazloff et al. (2010), the resistance surfaces analyzed in this study were interpolated (temperature and salinity) or resampled to reduce resolution (surface and seafloor elevation) to match the extent of the surface current speed layer for subsequent “mgLandscape” (MEMGENE) analysis (Appendix S4). While the interpolation of oceanic conditions may include prediction errors and deviation from true environmental conditions (especially in a heterogeneous environment) (Rellstab et al., 2015), the raster layers capture the overall dynamics of the Southern Ocean and serve as reasonable estimates for analyzing genetic‐environmental association at a circumpolar scale. Collinearity between selected environmental variables was checked using a pairwise Pearson correlation analysis using the R package “Raster” (Hijmans, 2016). The resulting correlation coefficients (r between −0.51 and 0.69) were below the threshold of collinearity (*r* < 0.7) in ecological datasets (Dormann et al., 2013) and were therefore appropriate for subsequent environmental association analysis.

The “mgLandscape” function of “MEMGENE” was used to characterize the MEM eigenvectors from the six resistance surfaces and Euclidean distances and to relate the MEM eigenvectors to genetic distance matrix using RDA. A matrix of genetic distance between individual COI sequences (i.e., sequences of both species were pooled together) was calculated based on the substitution model of TN+F+I+G4.

For “mgQuick” (within *O*. *victoriae*) and “mgLandscape” (between *O*. *victoriae* and *O*. *hexactis*) analyses, forward permutations of 500 were used to test for forward selection of MEM eigenvectors and final permutations of 1000 were used to test for significance levels at 0.05. Both “mgQuick” and “mgLandscape” produce values of adjusted *R* ^2^ (adj*R* ^2^) that estimate the overall proportion of the genetic variation that can and cannot be understood by each spatial predictor (“mgQuick”: Euclidean distances; “mgLandscape”: Euclidean distances, sea surface temperature, seafloor temperature, sea surface salinity, seafloor salinity, seafloor bathymetry, and surface current velocity).

## 3 RESULTS

### 3.1 Haplotype networks

**FIGURE 2:**
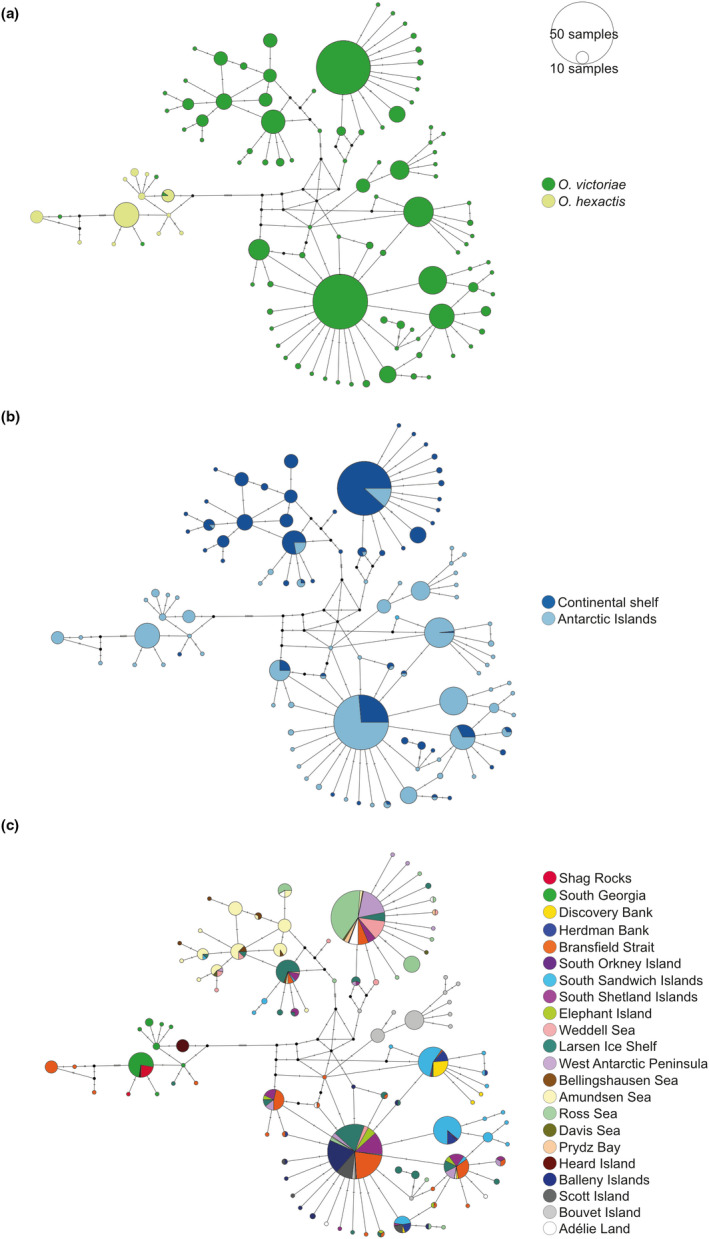
Median joining haplotype network of *Ophionotus victoriae* and *O. hexactis* COI sequences (434 bp, *n* = 935), separated by (a) species, (b) Antarctic continental shelf and Antarctic islands within the Antarctic Polar Front, and (c) location. Size and colors of circle represent the number of samples and sample locations associated with each haplotype. Black circle = inferred haplotype missing in the dataset. Hatch lines = inferred mutation steps between haplotypes

A total of 935 COI sequences of *O*. *victoriae* (*n* = 862) and *O*. *hexactis* (*n* = 73), comprised of 165 unique haplotypes, were included in data analysis. Median joining (MJ) network analysis of COI alignments revealed both species are highly separated but not perfectly reciprocally monophyletic groups and that *O*. *victoriae* is frequently connected forming a single haplotype network (Figure 2). TCS network also produced an identical conclusion to the MJ network (Appendix S5). Here, we discuss our network results based on the MJ network. Sample distribution revealed that both species were found on the Antarctic continental shelf and around Antarctic islands, with *O*. *hexactis* samples much more commonly collected around Antarctic islands than on the Antarctic shelf (Figure 2a,b).

On the *O*. *victoriae* side of the haplotype network, haplotypes from different sampled locations are dispersed throughout, thus forming a diffused network (Figure 2c). The network also shows some, but not complete, separation of continental shelf and sub‐Antarctic island haplotypes (Figure 2b). However, in many cases haplotypes are shared among *O*. *victoriae* sampled on the continental shelf and Antarctic islands (Figure 2b,c). Structured populations of *O*. *victoriae* are also observed around Bouvet Island, Amundsen Sea, and within the Scotia Sea.

For *O*. *hexactis*, the differentiation between sampled locations reflects structured populations within species. Close affinities were observed between Heard Island and South Georgia haplotypes via one or few mutational steps, with South Georgia haplotypes also linked to haplotypes in Shag Rocks, Bransfield Strait, and Antarctic Peninsula via a one or few mutational steps on the haplotype network (Figure 2c). Importantly, one *O*. *victoriae* individual from Heard Island off East Antarctica possessed the same haplotype as nine *O*. *hexactis* individuals collected within the same area (Figure 2c). The haplotypes of three *O*. *victoriae* individuals sampled from the Scotia Sea (two from South Georgia and one from Bransfield Strait) were also found to be nested within the *O*. *hexactis* haplotype network (Figure 2c).

### 3.2 Population genetic metrics

Genetic diversity differed between species and sampling locations. Overall, the nucleotide diversity was similarly high in *O*. *victoriae* (0.01801) and *O*. *hexactis* (*π* = 01801 and 0.01276, respectively), but higher haplotype diversity was detected in *O*. *victoriae* compared to *O*. *hexactis* (Hd = 0.887 and 0.715, respectively) (Table 1). A higher proportion of private haplotypes was also detected within *O*. *victoriae* (62%) compared to *O*. *hexactis* (54%). For *O*. *victoriae*, similar levels of nucleotide and haplotype diversity were found between samples on the continental shelf and sub‐Antarctic islands. However, the proportion of private haplotypes was generally higher around Antarctic islands (72% of all haplotypes found in waters around Antarctic islands) compared to continental shelf (51% of all haplotypes found on the shelf) (Table 1).

Within *O*. *victoriae*, the lowest genetic variation was detected around Scott Island and Balleny Islands (*π* < 0.00229 and Hd < 0.44) (Table 1). Conversely, high genetic variation in *O*. *victoriae* was found in most areas on the continental shelf including Bellingshausen Sea, West Antarctic Peninsula, Larsen Ice Shelf, Weddell Sea, Davis Sea, and Adélie Land (*π* > 0.01 and Hd > 0.7) (see Goodall‐Copestake et al., 2012 for global average of COI genetic diversity). However, *O*. *victoriae* in Prydz Bay, Ross Sea, and Amundsen Sea exhibited a relatively low level of nucleotide diversity (*π* between 0.00154 and 0.0069). Interestingly, while Prydz Bay and Ross Sea samples are characterized with medium level of haplotype diversity (Hd = 0.6 and 0.478, respectively), Amundsen Sea samples had a high haplotype diversity of 0.903. This low nucleotide diversity coupled with high haplotype diversity detected in Amundsen Sea samples may result from limited spatial sampling effort within the region, as all samples were collected within and around Pine Island Bay. For *O*. *victoriae* collected on the Antarctic continental shelf, a high proportion of private haplotypes (>50%) was found in the Bellingshausen Sea, Ross Sea, Adélie Land, Davis Sea, and Prydz Bay (53.3%) (Table 1). Among Antarctic islands, a high proportion of private haplotypes was detected from multiple island localities, including Heard Island, the Balleny Islands, Scott Island, Discovery Bank, Herdman Bank, Elephant Island, the South Sandwich Islands, Bransfield Strait, and Bouvet Island.

Within *O*. *hexactis*, the nucleotide diversity per locality was lowest around Shag Rocks (*π* = 0.00046) and highest on Heard Island (*π* = 0.00138) (Table 1). However, *O*. *hexactis* samples from Shag Rocks and Heard Island exhibited the same level of haplotypic diversity (Hd = 0.2). Although no private haplotypes were detected around Heard Island in *O*. *hexactis*, areas in the Scotia Sea (South Georgia, Shag Rocks, and Bransfield Strait) showed a relatively high proportion of private haplotypes (>50%) (Table 1).

### 3.3 Past species demography

**FIGURE 3:**
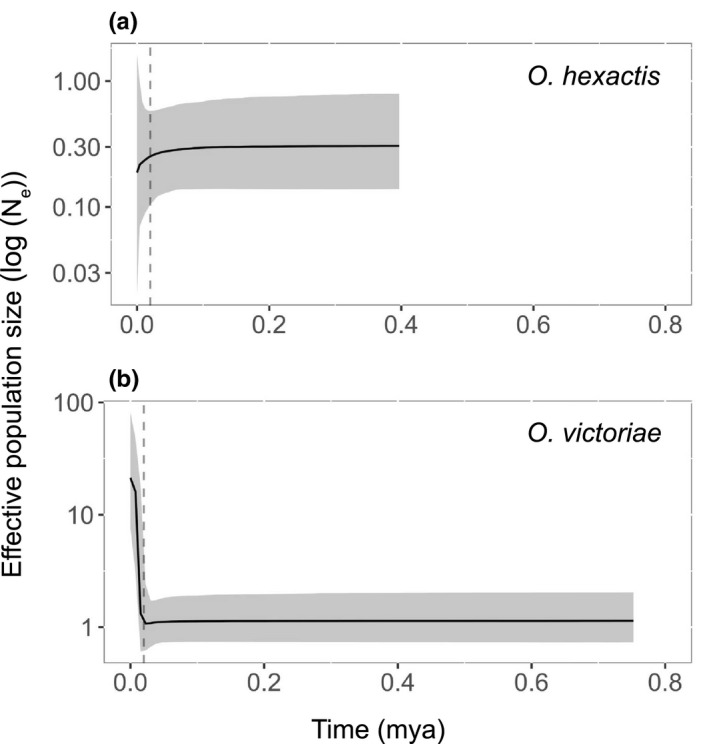
Bayesian skyline plots (BSP; log_10_ scale) of past effective population size of the Southern Ocean brittle stars *Ophionotus victoriae* and *O*. *hexactis* based on COI sequences. Dashed line represents the time of the last glacial maximum (~20,000 years ago)

Overall, evidence for a past population bottleneck and subsequent expansion was inferred in *O*. *victoriae* from all sample locations, as seen from a significantly negative Fu's *F_S_* value (−23.84, *p* = .007) and a unimodal mismatch distribution (Table 1). A BSP suggests a past population expansion was detected in *O*. *victoriae* on the shelf at around 20,000 years ago (Figure 3), coinciding with the timing of ice sheet retreat after the LGM. However, within each sample locality, signatures of past population bottlenecks and expansions appear to be ambiguous as non‐significant negative neutrality values and/or multimodal mismatch distribution were detected (Table 1).

In *O*. *hexactis*, the hypothesis of past population bottleneck and expansion was rejected due to non‐significant negative neutrality tests and multimodal mismatch distribution (Table 1). A BSP also indicated an overall stable population size over time in *O*. *hexactis* (Figure 3).

### 3.4 Genetic differentiation within and between species

When analyzing genetic structure through molecular variance, AMOVA via pairwise F_ST_ revealed a significant differentiation between species (*p* = .003) (Appendix S6). However, differentiation among sample locations (within species) represented only 22.64% of the overall genetic variation (AMOVA, *df* = 23, sum^2^ = 108.136, variance components = 0.121), and differentiation within sample locations for each species amounted to 61.48% of the overall variation (AMOVA, *df* = 910, sum^2^ = 297.784, variance components = 0.327) (Appendix S6). Pairwise F_ST_ showed a high and significant level of genetic differentiation between *O*. *victoriae* and *O*. *hexactis* (*F*_ST_ = 0.203, *p* < .0001) (Appendix S7). Within *O*. *victoriae*, pairwise *F*_ST_ showed low levels of differentiation between locations that are geographically proximal, including Elephant Island, Bransfield Strait, Shetland Islands in the Scotia Sea, and Balleny Islands and Scott Island (Appendix S8). Affinities between distant locations are also observed in *O*. *victoriae*, including West Antarctic Peninsula, Weddell Sea, Ross Sea, Adélie Land, and Prydz Bay, and also between Elephant Island, Shetland Islands, Balleny Islands, and Scott Island (Appendix S8). Within *O*. *hexactis*, pairwise *F*_ST_ indicated no significant differentiation between Shag Rocks and South Georgia, and Shag Rocks and Heard Island (Appendix S8).

### 3.5 Spatial pattern detection within *O. victoriae*

**FIGURE 4:**
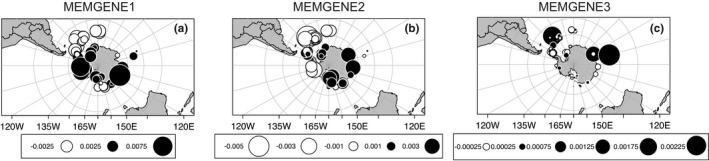
Visualization of spatial genetic patterns among *Ophionotus victoriae* samples based on the first three MEMGENE variables (“mgQuick”). Values alongside circles in the legend indicate MEMGENE score values. Circles of similar size and the same color represent individual sequence with similar scores on the MEMGENE axis (i.e., genetic similarities attributed to isolation‐by‐distance between samples). Overall, 46.0% of genetic variation can be explained by spatial scale (adj*R* ^2^ = 0.460). (a) MEMGENE1 shows a strong spatial pattern of two genetic clusters distinct to the continental shelf and Antarctic islands near the Antarctic Polar Front which contributes 57.2% of the adj*R* ^2^. (b) MEMGEN2 shows the second strongest spatial pattern of connectivity between Amundsen Sea, West Antarctic Peninsula, Scotia Sea and Bouvet Island which contributes 31.8% of the adj*R* ^2^. (c) MEMGENE3 shows the third strongest spatial pattern demonstrating structure connecting Scotia Sea, Heard Island and Prydz Bay, which contributes 4.78% of the adj*R* ^2^

Spatial pattern detection analysis (MEMGENE) revealed that variation within *O*. *victoriae* was discernible when comparing genetic distance between individual COI sequences. MEMGENE analysis suggested 46.0% of overall genetic variation can be explained by spatial scale (adj*R* ^2^ = 0.460). MEMGENE1, the variable that represented the strongest spatial pattern detected by MEMGENE (57.2% of the adj*R* ^2^), showed clear genetic divergence between continental shelf and most island localities (Scotia Sea + Bouvet Island + Balleny Islands + Scott Island) (Figure 4a). However, the genetic structure of the continental shelf and Antarctic islands does not appear to be independent of each other as MEMGENE1 also detected genetic similarity among island localities and Prydz Bay (Figure 4a). Samples from Heard Island also showed genetic similarity from continental shelf samples (Figure 4a). MEMGENE2, the variable that explains the second strongest spatial pattern (31.8% of the adj*R* ^2^), further indicates relatedness between the continental shelf and Antarctic islands (Figure 4b). In particular, a strong regional structure is observed between Amundsen Sea, West Antarctic Peninsula, Scotia Sea, and Bouvet Island (Figure 4b). MEMGENE3, the variable that explains most of the remaining spatial structure in the dataset (4.78% of the adj*R* ^2^), demonstrated clear spatial structure connecting Scotia Sea, Heard Island, and Prydz Bay (Figure 4c).

### 3.6 Isolation‐by‐environment between species

Isolation‐by‐environment analysis (via analyzing resistance surfaces) indicates that isolation‐by‐geographical distance (Euclidean distances), ocean surface and seafloor temperature, surface and seafloor salinity, surface current velocity, and bathymetry were all significant in explaining spatial genetic variation between *O*. *victoriae* and *O*. *hexactis* (*p *< .001; Table 2). Euclidean distances appeared to be the best spatial predictor in explaining the observed genetic variations ([a] adj*R* ^2^ = 0.426), followed by bathymetry ([a] adj*R* ^2^ = 0.390), surface temperature ([a] adj*R* ^2^ = 0.373), surface salinity ([a] adj*R* ^2^ = 0.271), surface current speed ([a] adj*R* ^2^ = 0.265), seafloor salinity ([a] adj*R* ^2^ = 0.264), and seafloor temperature ([a] adj*R* ^2^ = 0.237) (Table 2).

**TABLE 2:** Results of Isolation‐by‐environment (“mgLandscape”) analysis comparing the proportion of spatial genetic variation between *Ophionotus* *victoriae* and *O. hexactis* (adj*R* ^2^) explained and not explained by environmental parameters of present‐day condition

Model	[abc]	P[abc]	[a]	P[a]	[c]	P[c]	[b]	[d]
IBD	0.518	0.001	0.426	0.001	0.520	0.001	0.403	0.482
Water depth (m)	0.483	0.001	0.390	0.001	0.512	0.001	0.411	0.517
Surface temperature (°C)	0.465	0.001	0.373	0.001	0.510	0.001	0.414	0.535
Surface salinity	0.364	0.001	0.271	0.001	0.198	0.001	0.725	0.636
Seafloor salinity	0.357	0.001	0.264	0.001	0.227	0.001	0.696	0.643
Surface current speed (m s^−1^)	0.357	0.001	0.265	0.001	0.211	0.001	0.712	0.643
Seafloor temperature (°C)	0.329	0.001	0.237	0.001	0.179	0.001	0.744	0.671

Note

Environmental parameters considered including isolation‐by‐distance (IBD), surface and seafloor temperature, surface and seafloor salinity, surface current speed, and water depth. Numbers in table represent the adj*R* ^2^ explained by [abc] spatial predictors (MEM eigenvectors), [a] spatial patterns in given model, [c] coordinates, [b] confounded pattern between given model and coordinates, [d] residuals not explained by spatial predictors. P[abc], P[a], P[c] represent the significance value calculated for each proportion with significance level at *p* = .05.

## 4 DISCUSSION

### 4.1 *Ophionotus victoriae* as a single entity based on COI data

Rather than comprising multiple cryptic species as proposed by previous studies (Galaska et al., 2017a; Hunter & Halanych, 2010), the haplotype network of *O*. *victoriae* is frequently connected, suggesting this species is one entity with a circumpolar distribution in the Southern Ocean. In the previously published studies utilizing Southern Ocean COI datasets, *O*. *victoriae* had been collected from relatively few locations in West Antarctica (Galaska et al., 2017a; Hunter & Halanych, 2010). Additionally, the existing SNP dataset of *O*. *victoriae* is comprised of samples from an even more restricted distribution, with individuals collected from more disjunct locations (Galaska et al., 2017a). In this study, we have utilized an expanded analysis with an updated sampling coverage representing individuals collected along a geographical, circumpolar cline rather than from disjunct locations. After incorporating the additional *O*. *victoriae* samples (*n* = 443) to the published COI sequences in the haplotype network, *O*. *victoriae* is characterized by a single connected network rather than multiple clusters (as found in Galaska et al., 2017a). Therefore, the new samples included in this study represent the missing links that connect divergent lineages described in previous studies. The previous interpretation of multiple cryptic species was likely caused by the limited spatial sampling of a genetically diverse and widely distributed species, where “individual clusters” likely represented artifacts driven by isolation‐by‐distance.

### 4.2 Genetic relationship between *O*. *victoriae* and *O. hexactis*

Although current taxonomy and previous studies (Galaska et al., 2017a; Hugall et al., 2016) recognized *O*. *victoriae* and *O*. *hexactis* are separate species, our analyses, including the haplotype network and phylogenetic trees (ML and BI), show the two taxa to be highly separated, but not perfectly reciprocally monophyletic groups. In the current dataset, the distributions of *O*. *victoriae* and *O*. *hexactis* overlap around Heard Island, as well as around Bransfield Strait and South Georgia in the Scotia Arc. We observed *O*. *victoriae* samples from these three areas within the *O*. *hexactis* clade (including all *O*. *victoriae* samples from Heard Island and South Georgia). Coincidentally, this dataset contains a very low sample size of *O*. *victoriae* from Heard Island (*n* = 1) and South Georgia (*n* = 2). However, a relatively higher sample size of *O*. *victoriae* from Bransfield Strait was included in this study (*n* = 97, including *n* = 67 from newly sequenced samples). Out of the 97 samples, only one sample fell within the *O*. *hexactis* clade (ID: SIO‐BIC E5524E). Furthermore, eight other *O*. *victoriae* samples collected from the same trawl containing as SIO‐BIC E5524E did not fall within the *O*. *hexactis* clade. *Ophionotus victoriae* seems to share an unusually close genetic relationship (in terms of COI data) with *O*. *hexactis* in locations where they overlap. However, from the sampled diversity of *O*. *victoriae* in Bransfield Strait, it appears that not all *O*. *victoriae* exhibit the similarly close genetic distance with *O*. *hexactis* under the same environmental opportunity.

Species‐level paraphyly and haplotype affinities between *O*. *victoriae* and *O*. *hexactis* could represent either incomplete lineage sorting or hybridization following secondary contact (McKay & Zink, 2010). There are parallel explanations that may explain this. First, only one haplotype shared between *O. victoriae and O*. *hexactis* was detected in this study, collected from around Heard Island. Heard Island is a remote island on the Kerguelen Plateau and is separated from the main Antarctic continental shelf and other Antarctic islands outside of the Plateau via the deep sea and a long geographical distance (Griffiths et al., 2008). It is possible that the unique environmental setting of Heard Island allowed the two species to hybridize, which enabled mitochondrial introgression to occur between species in that location, while geographical isolation prevented the shared haplotypes from spreading to other localities. Secondly, there are *O*. *victoriae* individuals possessing haplotypes within the *O*. *hexactis* clade that are not shared by the two species. This could reflect either incomplete lineage sorting, or signatures of hybridization and introgression in the past followed by mutation.

While *O*. *victoriae* and *O*. *hexactis* exhibit contrasting reproductive strategies (broadcast spawning and brooding, respectively), previous studies have suggested sperm chemotaxis (sperm recognition of eggs) appears to be species‐specific in most, but not all, brittle stars (Miller, 1998; Weber et al., 2017). Therefore, as well as the overlapping distribution of *O*. *victoriae* and *O*. *hexactis*, physiological opportunities enabling intraspecific hybridization may also exist. Strong incomplete lineage sorting and past hybridization have also been detected among six cryptic brittle stars species *Ophioderma* spp. with brooding or broadcast spawning strategies (Weber et al., 2017). As we only utilized a single mitochondrial marker (COI) which is a maternally inherited marker, our data could be influenced by selection, as well as bias toward the history of mitochondrial lineages that may be incongruent with species history, and the history of maternal lineages in the event of sex‐biased dispersal (Sloan et al., 2017). Overall, we highlight an interesting additional case of possible incomplete lineage sorting or hybridization between two sister taxa with contrasting morphology and life history for future multi‐locus studies.

### 4.3 Contrasting signals of Southern Ocean refugia between species

Evidence of deep‐sea refugia in *O*. *victoriae* is demonstrated through the overall absence of population bottleneck signatures (summary statistics), combined with signs of population expansion after the LGM (BSP). Results of BSP can be confounded by the effect of population structure (Heller et al., 2013); therefore, results should be interpreted with caution. However, the overall high haplotypic diversity and the “diffused” pattern in the haplotype network also suggest *O*. *victoriae* continued to diversify during glacial periods (Allcock & Strugnell, 2012). These patterns of past population size change and population connectivity point toward the key characteristics associated with deep‐sea refugia survival (Lau et al., 2020). The deep sea was hypothesized as the only large scale, ice‐free habitable area that could support a large population size and continued diversification during glacial periods; in comparison, the Antarctic continental shelf was largely covered in grounded ice (Thatje et al., 2005). While evidence of LGM grounded ice was observed around some Antarctic islands (Elephant Island, Bouvet Island, Heard Island), areas free of grounded ice were also observed around South Georgia and South Sandwich Islands during the LGM (Barnes et al., 2016; Graham et al., 2008; Hodgson et al., 2014). However, the habitable areas around Antarctic Islands are small and restricted relative to the deep sea, especially for steep, volcanic islands. Therefore, islands are unlikely to have supported the continued diversification throughout glacial periods. The eurybathic distribution of *O*. *victoriae* (34–1750 m; the sampled depth range of this study) further supports its capability to migrate to deep‐sea refugia and then subsequently recolonize the shelf after the LGM. Association between eurybathic distributions and deep‐sea refugia was also suggested in the Southern Ocean shrimp *Nematocarcinus lanceopes* (Raupach et al., 2010) and the sea spider *Nymphon australe* (Soler‐Membrives et al., 2017).

Evidence of Antarctic island refugia for *O*. *hexactis* is demonstrated through a stable population structure throughout glacial maxima, as seen from the lack of strong population bottlenecks and the absence of population expansion (summary statistics and BSP). The structured populations between distant locations (e.g., Heard Island and the Scotia Arc) reflected in the haplotype network are also suggestive of *in situ* survival within these locations. The known depth and range distribution of *O*. *hexactis*, which is restricted to the shallow Southern Ocean mainly around Antarctic islands (0–459 m, GBIF.org, 2019), also support the case of *in situ* survival within island refugia. Given that connectivity between the Scotia Arc and Heard Island are detected on the haplotype network (i.e., haplotypes from both locations separated by one mutation step), this long‐distance connectivity is probably facilitated by rafting, as suggested in other Southern Ocean benthic fauna also with brooding characteristic (Helmuth et al., 1994; Leese et al., 2010; Nikula et al., 2010).

Isolation‐by‐environment analysis also indicated the spatial genetic patterns between *O*. *victoriae* and *O*. *hexactis* were most associated with geographic distance and water depth, suggesting isolation‐by‐geographical distance and depth. Isolation‐by‐distance and depth are expected when populations have been stable over time, with gene flow occurring more often between spatially neighboring populations, as well as selective ecological forces and reproductive barrier between diverging populations (Wright, 1943). Since the depth range of *O*. *victoriae* extends into the deep sea while *O*. *hexactis* is only known from relatively shallow water, the isolation‐by‐depth pattern might reflect a stepwise recolonization pattern in *O*. *victoriae* from deep‐sea refugia to the continental shelf along the seafloor bathymetry after the LGM. Given that there is also a distributional difference between *O*. *victoriae* and *O*. *hexactis*, whereby *O*. *victoriae* inhabits the deep sea, Antarctic continental shelf, and Antarctic islands, and *O*. *hexactis* is mainly observed around Antarctic islands, the strong isolation‐by‐distance pattern detected likely reflects genetic differentiation between the two species.

### 4.4 Evolutionary implications of different refugial uses in the Southern Ocean

In the Southern Ocean, glacial cycles have been hypothesized to drive allopatric speciation due to populations being contained within isolated refugia on the continental shelf (i.e., the Antarctic biodiversity pump hypothesis) (Clarke & Crame, 1989, 1992; Crame, 1997). In the case of *Ophionotus* spp., the evidence suggests that *O*. *victoriae* and *O*. *hexactis* have taken refuge within independent, largely non‐overlapping environments (the deep sea and islands, respectively). Interestingly, a recent study also presented a seemingly similar case to *Ophionotus*, in which a brooding clade (clade V) was reported in the Antarctic brittle star *Astrotoma agassizii* species complex around South Georgia, and a broadcast spawning clade (clade I) distributed on both the Antarctic continental shelf and around South Georgia (Jossart et al., 2019). Both sympatric cryptic species are also characterized by a clear size dimorphism (larger and smaller body size in clade I and V, respectively) (Jossart et al., 2019). These clades with contrasting life‐history strategies were also previously reported in Galaska et al. (2017b) but were not known then to be sympatric. Although the evolutionary history of the Southern Ocean *A*. *agassizii* complex was not examined by Jossart et al. (2019), the reported significantly negative values obtained from neutrality tests (Tajima's *D* and Fu's *F_S_*) indicate both clades exhibited signatures of strong population bottlenecks, suggesting *in situ* persistence throughout glacial cycles. The star‐like haplotype networks of clade I and V of *A*. *agassizii* also support a likely scenario of *in situ* refugia in these areas (discussed within Allcock & Strugnell, 2012). The current data suggest South Georgia served as a sub‐Antarctic glacial refugia for both *A*. *agassizii* (clade I and V) and *O*. *hexactis*. For the Southern Ocean ophiuroids that are currently living on the continental shelf, *O*. *victoriae* historically found refuge in the deep sea while *A*. *agassizii* (clade I) likely persisted on the shelf over glacial periods, highlighting that refugium survival can be different between brittle stars with the same reproductive strategy (broadcast spawning).

Both the cases of the Southern Ocean *Ophionotus* spp. and *A*. *agassizii* (Clades I and V) show different morphologic traits are observed between closely related species, including size dimorphism between the two clades in *A*. *agassizii*, and different arm numbers in *O*. *victoriae* and *O*. *hexactis*. For the case of *A*. *agassizii*, the two closely related species cannot coexist in sympatry without evolving character displacement (competitive exclusion principle) (Hardin, 1960). However, survival in independent glacial refugia (deep sea and Antarctic islands) is also associated with character changes between *O*. *victoriae* and *O*. *hexactis*. Our data highlight that species histories can vary among Southern Ocean taxa, even within the same class (i.e., the case of ophiuroids presented here). Another interesting aspect common in both cases is that brooding as a reproductive trait is mostly exclusive to Antarctic islands, indicating brooding could be positively selected around the islands.

### 4.5 Connectivity and isolation between the continental shelf and Antarctic islands

Spatial pattern detection analysis (“mgQuick”) and the haplotype network within *O*. *victoriae* support signatures of both isolation and connectivity, within and between, the Antarctic continental shelf and Antarctic islands that could also be linked to physical transports in the Southern Ocean. First, the strong spatial genetic structures detected by MEMGENE1 that separate the continental shelf and some Antarctic islands (Scotia Arc, Bouvet Island, Balleny Islands, Scott Islands) coincide with the southern frontal structure of the ACC (southern boundary ACC and southern ACC) in the Southern Ocean (Sokolov & Rintoul, 2009). However, as detected by MEMGENE1, 2, and 3 within *O*. *victoriae*, patterns connecting continental shelf localities (Prydz Bay and Amundsen Sea) and Antarctic islands were also observed, indicating a permeable barrier between the two environments.

Within *O*. *victoriae*, similarity was detected between the Scotia Sea, Bouvet Island, and Prydz Bay in MEMGENE1, and between the Scotia Sea, Heard Island, and Prydz Bay in MEMGENE3. Connectivity between the Scotia Sea and Bouvet Island has been observed in the notothenioid *Lepidonotothen larseni* (Damerau et al., 2014). Also, a Scotia Sea–Prydz Bay connection pathway has also been previously described in other Southern Ocean taxa including the asteroid *Glabraster antarctica* (Moore et al., 2018), the octopod *Pareledone turqueti* (Strugnell et al., 2012), the amphipod *Eusirus giganteus* (Baird et al., 2011), and the crinoid *Promachocrinus* phylogroup C and F (Hemery et al., 2012), highlighting a probable seascape corridor that enables gene flow between Antarctic islands and continental shelf in some Southern Ocean benthic taxa. Given that the Scotia Sea, Bouvet Island, Prydz Bay, and Heard Island are separated by a long geographical distance, there are likely unsampled regions between these areas that contribute to this proposed long‐distance connectivity. The regional spatial genetic structure detected by MEMGENE2, found between the Amundsen Sea, Antarctic Peninsula, Scotia Sea, and Bouvet Island, also likely reflects the influence of local oceanographic dynamics into and beyond the Scotia Sea including the eastward flowing ACC (Maldonado et al., 2003).

### 4.6 Life history and morphological innovation in *O*. *hexactis* during glacial periods

In echinoderms, brooding has often emerged under environmental stressful conditions during species selection over macroevolutionary timeframes (Lawrence & Herrera, 2000), even though this strategy requires higher maternal investment compared with pelagic larval development (Fernández et al., 2000). Previous studies have also suggested that, after a shift to hyper‐oligotrophy in the Eastern Mediterranean region, the brittle star *Ophioderma zibrowii* with a brooding characteristic emerged from a broadcast spawning lineage (Boissin et al., 2011; Stöhr et al., 2020; Weber et al., 2019), suggesting brooding character can emerge due to historical environmental changes. Furthermore, in the Southern Ocean, brooding as a characteristic has also been hypothesized as a result of selection from non‐present‐day environmental conditions, rather than an adaptation to generic polar conditions (Pearse et al., 2009). However, an increase in arm number in echinoderms has yet to be linked to changes in past environmental conditions. Nonetheless, laboratories experiments have reported exposure to high salinity and low pH can result in arm number changes in the asteroid *Echinaster* sp. and in the ophiuroid *Ophiothrix fragilis*, respectively, under experimental conditions (Dupont et al., 2008; Watts et al., 1983). Furthermore, an increase in arm number (more than 5 arms) in ophiuroids is positively correlated to coordinated locomotion (Clark et al., 2019) and can be linked to increasingly random escape patterns (thus non‐predictable escape strategies) (Wakita et al., 2020). Therefore, the six‐arm innovation in *O*. *hexactis* could be related to selective forces linked to changes in environmental conditions (such as salinity or pH) or ecological settings that would require increased coordination. Our study also implies that the morphological and life‐history differences observed between *O*. *victoriae* and *O*. *hexactis* could be linked to strong environmental stressors in the past.

While the modern Southern Ocean seafloor is characterized by marked gradients of low temperature (−2.1 to 2.8°C) and salinity (34.2–34.7 psu), the surface of the Southern Ocean is comprised of a series of sharp temperature (<1.5–4°C) and salinity (33.6–34.4) fronts that divide the subtropical (warmer water, saline in the North) and polar fronts (colder, fresher water in the South) (Locarnini et al., 2018; Zweng et al., 2018). Isolation‐by‐environment analysis indicated the spatial genetic pattern between *O*. *victoriae* and *O*. *hexactis* showed stronger associations with sea surface temperature + salinity gradients, compared to associations with seafloor temperature + salinity gradients, despite the two species being benthic species. *Ophionotus victoriae* and *O*. *hexactis* have been suggested to have diverged at 1.64 million years ago (mya; 0.53–5.79 mya) during the Pleistocene (O'Hara et al., 2019), and thus, it is unlikely that the morphological innovation in *O*. *hexactis* was influenced by modern temperature and salinity patterns. Instead, the results likely reflect the divergence between *O*. *victoriae* and *O*. *hexactis* is linked to strong environmental gradients separating the two species in the past. For example, the prolonged glacial‐interglacial cycles, or fluctuation in salinity and overall lower salinity throughout the late Pleistocene, or other environmental changes linked to intensified glacial‐interglacial cycles, were likely the key environmental drivers linked to evolutionary innovations in *O*. *hexactis*. Notably, in the past 1.5 million years, the glacial‐interglacial cycles transitioned from 41 kyr cycles with low amplitude to 100 kyr cycles with intensification in climatic cycles after the mid‐Pleistocene transition (Clark et al., 2006). Since *O*. *hexactis* persisted in Antarctic island refugia in the shallow Southern Ocean, which would have been directly exposed to the prolonged, as well as intensified elements of glaciations and interglacial periods. Additionally, the rapid deglaciation at the beginning of each interglacial cycle should lead to a rapid and steep decline in salinity in the surface Southern Ocean. *Ophiontous hexactis* around Antarctic islands would have been directly and repeatedly exposed to deglacial meltwater after each glacial maximum. It has also been recently suggested that the surface Southern Ocean consisted of a lower level of salinity during glacial maxima (~33.4 psu relative to ~34.56 psu in the deep sea) (Hasenfratz et al., 2019). Therefore, it is also plausible that the rapid and steep decline in salinity during intensified interglacial cycles could have driven the character changes in *O*. *hexactis*, and the overall lower salinity during glacial cycles also maintained such innovations.

Alternatively, an increase in arm number and brooding strategy may not be directly linked to the proposed selective forces during the Pleistocene. The increase in arm number could be linked to ecosystem dynamics around Antarctic islands leading to enhanced coordination or could have simply arisen as a by‐product of vicariance. The advantageous nature of brooding during glacial periods, when the Southern Ocean experienced limited habitat availability and low primary productivity, is more widely accepted (Convey et al., 2009; Pearse et al., 2009; Poulin et al., 2002; Thatje et al., 2005). Nonetheless, the establishment of the morphological difference between *O*. *victoriae* and *O*. *hexactis* could have occurred prior, during, or after lineage splitting. The two characteristic morphological changes in *O*. *hexactis* might also not have happened simultaneously, as arm number and reproductive mode are functionally different. Each innovation leading to ecological success would have been driven by different ecological opportunities, and possibly occurred on independent occasions.

## 5 CONCLUSION

This study suggests that *O*. *victoriae* is a single species and is closely related to *O*. *hexactis* based on COI data. Although there could be incomplete lineage sorting, or hybridization between *O*. *victoriae* and *O*. *hexactis*, the contrasting morphology and life‐history traits support the current taxonomic recognition of two species. The broader implications of this study demonstrate how glacial cycles and oceanic currents have structured genetic patterns in Southern Ocean benthic taxa. While *O*. *victoriae* and *O*. *hexactis* appear to have found refuge in different environments during glacial periods (the deep sea and Antarctic islands, respectively), we highlight that Southern Ocean species with similar life histories can survive in different types of glacial refugia (e.g., the parallel case of *A*. *agassizii* (clade I) and *O. victoriae*).

Our data also demonstrate distinct genetic clusters between the Antarctic continental shelf and Antarctic islands near the Antarctic Polar Front, coinciding with the frontal boundary of the ACC in the Southern Ocean. However, genetic connectivity between the Scotia Sea and Prydz Bay was also detected, suggesting connectivity between the Antarctic islands and the Antarctic continental shelf is ongoing. Genetic structure observed between the Scotia Sea and neighboring regions (Bellingshausen Sea, Antarctic Peninsula, Weddell Sea, and Bouvet Island) also highlights the role of the ACC and Weddell gyre in structuring regional genetic patterns.

Finally, our work also discussed that the morphological and life‐history innovation in *O*. *hexactis* (an increase in arm number and brooding as reproductive strategy) can be linked to selection from environmental conditions in the past, which was first proposed by Pearse et al. (2009). Further work examining genetic structure in *O*. *victoriae* and *O*. *hexactis* using nuclear data should provide a thorough understanding of the genetic relationship between the two species, and signatures of past environmental selection.

## CONFLICT OF INTEREST

The authors declare no competing interests.

## AUTHOR CONTRIBUTIONS

**Sally C. Y. Lau**: Formal analysis (lead); funding acquisition (supporting); investigation (lead); writing‐original draft (lead); writing‐review & editing (Equal). **Jan M. Strugnell**: Conceptualization (equal); formal analysis (equal); funding acquisition (equal); investigation (equal); supervision (equal); writing‐review & editing (equal). **Chester J. Sands**: Data curation (supporting); formal analysis (supporting); investigation (supporting); writing‐review & editing (equal). **Catarina N**. **S. Silva**: Formal analysis (equal); investigation (equal); supervision (supporting); writing‐review & editing (equal). **Nerida G. Wilson**: Conceptualization (equal); data curation (lead); formal analysis (equal); funding acquisition (equal); investigation (equal); supervision (equal); writing‐review & editing (equal).

## Supporting information

Supplementary Material

## Data Availability

All newly generated COI sequences were deposited to GenBank at NCBI under accession numbers MZ543435—MZ543949.
