# Supplementary material for: Evolutionary innovations in Antarctic brittle stars linked to glacial refugia
Source: Ecol Evol. 2021 Nov 29;11(23):17428–46. doi: 10.1002/ece3.8376 (PMC8668817; doi:10.1002/ece3.8376)
Supplement: Supplementary file 1 — Supplementary Material [file ECE3-11-17428-s001.docx]

Evolutionary innovations in Antarctic brittle stars linked to glacial refugia

Authors & affiliations: Sally C.Y. Lau^1^, Jan M. Strugnell^1,2^, Chester J. Sands^3,^ Catarina N.S. Silva^1^, Nerida G. Wilson^4,5^

^1^Centre for Sustainable Tropical Fisheries and Aquaculture and College of Science and Engineering, James Cook University, Townsville, Qld 4810, Australia

^2^Department of Ecology, Environment and Evolution, School of Life Sciences, La Trobe University, Kingsbury Drive, Melbourne, Vic 3086, Australia

^3^British Antarctic Survey, Natural Environment Research Council, Cambridge, UK

^4^Collections & Research, Western Australian Museum, 49 Kew Street, Welshpool, WA 6106, Australia

^4^School of Biological Sciences, University of Western Australia, Crawley, Perth, WA 6009, Australia

**Appendices**

**Appendix S1**. Sample information of all collected samples newly sequenced for this study, and GenBank accessions and associated sampling information from previous studies used in this study.

| **Genus** | **Species** | **Field ID** | **Registration ID** | **Sample locality (defined in this study)** | **Haplotype number in this study** | **GenBank accession from previous studies (if applicable)** | **Collection year** | **Expedition** | **Station ID** | **Event number** | **Latitude** | **Longitude** | **Collection depth (m)** |
| --- | --- | --- | --- | --- | --- | --- | --- | --- | --- | --- | --- | --- | --- |
| Ophionotus | victoriae |  | IE.2009.4672 | Adélie Land | 1 |  | 2008 | CEAMARC | 70EV451 |  | -66.40862 | 140.50817 | 1204 |
| Ophionotus | victoriae |  | IE.2009.4676 | Adélie Land | 1 |  | 2008 | CEAMARC | 70EV451 |  | -66.40862 | 140.50817 | 1204 |
| Ophionotus | victoriae |  | IE.2009.4679 | Adélie Land | 1 |  | 2008 | CEAMARC | 70EV451 |  | -66.40862 | 140.50817 | 1204 |
| Ophionotus | victoriae |  | IE.2009.4690 | Adélie Land | 1 |  | 2007 | CEAMARC | 40EV152 |  | -66.6514 | 142.95743 | 637 |
| Ophionotus | victoriae |  | IE.2009.4726 | Adélie Land | 1 |  | 2007 | CEAMARC | 39EV141 |  | -66.54985 | 142.95882 | 875 |
| Ophionotus | victoriae |  | IE.2009.4734 | Adélie Land | 1 |  | 2008 | CEAMARC | 31EV268 |  | -66.53853 | 144.9725 | 451 |
| Ophionotus | victoriae |  | IE.2009.4753 | Adélie Land | 1 |  | 2008 | CEAMARC | 71EV447 |  | -66.38878 | 140.42885 | 791 |
| Ophionotus | victoriae |  | IE.2009.4763 | Adélie Land | 1 |  | 2008 | CEAMARC | 59EV259 |  | -66.73872 | 144.30702 | 954 |
| Ophionotus | victoriae |  | IE.2009.4675 | Adélie Land | 3 |  | 2008 | CEAMARC | 22EV503 |  | -65.99122 | 139.30742 | 485 |
| Ophionotus | victoriae |  | IE.2009.4754 | Adélie Land | 6 |  | 2007 | CEAMARC | 30EV66 |  | -66.00395 | 143.71608 | 440 |
| Ophionotus | victoriae | IE.2009.4687A | IE.2009.4687 | Adélie Land | 49 |  | 2008 | CEAMARC | 10EV420 |  | -66.3351 | 141.27267 | 227 |
| Ophionotus | victoriae |  | IE.2009.4702 | Adélie Land | 49 |  | 2007 | CEAMARC | 9EV117 |  | -66.53482 | 141.98268 | 521 |
| Ophionotus | victoriae |  | IE.2009.4703 | Adélie Land | 50 |  | 2008 | CEAMARC | 3EV411 |  | -66.0003 | 142.0143 | 248 |
| Ophionotus | victoriae |  | IE.2009.4707 | Adélie Land | 51 |  | 2008 | CEAMARC | 1EV405 |  | -66.00388 | 142.31378 | 240 |
| Ophionotus | victoriae |  | IE.2009.4713 | Adélie Land | 52 |  | 2007 | CEAMARC | 9EV117 |  | -66.53482 | 141.98268 | 521 |
| Ophionotus | victoriae |  | IE.2009.4731 | Adélie Land | 53 |  | 2007 | CEAMARC | 30EV66 |  | -66.00395 | 143.71608 | 440 |
| Ophionotus | victoriae |  | IE.2009.4767 | Adélie Land | 54 |  | 2007 | CEAMARC | 4EV112 |  | -66.31638 | 142.00037 | 257 |
| Ophionotus | victoriae | DSOPH2346 |  | Amundsen Sea | 1 | FJ917337 | 2008 | JR179 | BIO6-AGT-1B |  | -71.152 | -110.013 | 1491 |
| Ophionotus | victoriae | DSOPH2571 |  | Amundsen Sea | 1 | FJ917337 | 2008 | JR179 | BIO6-AGT-2A |  | -71.175 | -109.863 | 1080 |
| Ophionotus | victoriae | DSOPH2204 |  | Amundsen Sea | 14 | FJ917310 | 2008 | JR179 | BIO6-AGT-2A |  | -71.175 | -109.863 | 1080 |
| Ophionotus | victoriae | DSOPH2257 |  | Amundsen Sea | 25 | FJ917319 | 2008 | JR179 | BIO6-AGT-2C |  | -71.182 | -109.926 | 987 |
| Ophionotus | victoriae | DSOPH2568 |  | Amundsen Sea | 43 | FJ917348 | 2008 | JR179 | BIO4-AGT-2C |  | -74.477 | -104.257 | 1151 |
| Ophionotus | victoriae | DSOPH2203 |  | Amundsen Sea | 49 | KY048234 | 2008 | JR179 | BIO6-AGT-2A |  | -71.175 | -109.863 | 1080 |
| Ophionotus | victoriae | DSOPH2207 |  | Amundsen Sea | 49 | KY048234 | 2008 | JR179 | BIO6-AGT-2A |  | -71.175 | -109.863 | 1080 |
| Ophionotus | victoriae | DSOPH2218 |  | Amundsen Sea | 49 | KY048234 | 2008 | JR179 | BIO6-AGT-2A |  | -71.175 | -109.863 | 1080 |
| Ophionotus | victoriae | DSOPH2198 |  | Amundsen Sea | 58 | KY048231 | 2008 | JR179 | BIO6-AGT-2B |  | -71.179 | -109.894 | 998 |
| Ophionotus | victoriae | DSOPH2212 |  | Amundsen Sea | 58 | KY048231 | 2008 | JR179 | BIO6-AGT-2A |  | -71.175 | -109.863 | 1080 |
| Ophionotus | victoriae | DSOPH2251 |  | Amundsen Sea | 58 | KY048231 | 2008 | JR179 | BIO6-AGT-2C |  | -71.182 | -109.926 | 987 |
| Ophionotus | victoriae | DSOPH2678 |  | Amundsen Sea | 58 | KY048231 | 2008 | JR179 | BIO4-AGT-2C |  | -74.477 | -104.257 | 1151 |
| Ophionotus | victoriae | DSOPH2685 |  | Amundsen Sea | 58 | KY048231 | 2008 | JR179 | BIO4-AGT-2C |  | -74.477 | -104.257 | 1151 |
| Ophionotus | victoriae | DSOPH2734 |  | Amundsen Sea | 58 | KY048231 | 2008 | JR179 | BIO6-AGT-2B |  | -71.179 | -109.894 | 998 |
| Ophionotus | victoriae | DSOPH2186 |  | Amundsen Sea | 62 | KY048226 | 2008 | JR179 | BIO6-AGT-2B |  | -71.179 | -109.894 | 998 |
| Ophionotus | victoriae | DSOPH2215 |  | Amundsen Sea | 62 | KY048226 | 2008 | JR179 | BIO6-AGT-2A |  | -71.175 | -109.863 | 1080 |
| Ophionotus | victoriae | DSOPH2217 |  | Amundsen Sea | 62 | KY048226 | 2008 | JR179 | BIO6-AGT-2A |  | -71.175 | -109.863 | 1080 |
| Ophionotus | victoriae | DSOPH2254 |  | Amundsen Sea | 62 | KY048226 | 2008 | JR179 | BIO6-AGT-2C |  | -71.182 | -109.926 | 987 |
| Ophionotus | victoriae | DSOPH2256 |  | Amundsen Sea | 62 | KY048226 | 2008 | JR179 | BIO6-AGT-2C |  | -71.182 | -109.926 | 987 |
| Ophionotus | victoriae | DSOPH2258 |  | Amundsen Sea | 62 | KY048226 | 2008 | JR179 | BIO6-AGT-2C |  | -71.182 | -109.926 | 987 |
| Ophionotus | victoriae | DSOPH2684 |  | Amundsen Sea | 62 | KY048226 | 2008 | JR179 | BIO4-AGT-2C |  | -74.477 | -104.257 | 1151 |
| Ophionotus | victoriae | DSOPH2699 |  | Amundsen Sea | 62 | KY048226 | 2008 | JR179 | BIO6-AGT-2A |  | -71.175 | -109.863 | 1080 |
| Ophionotus | victoriae | DSOPH2743 |  | Amundsen Sea | 62 | KY048226 | 2008 | JR179 | BIO6-AGT-2B |  | -71.179 | -109.894 | 998 |
| Ophionotus | victoriae | DSOPH2187 |  | Amundsen Sea | 63 | KY048227 | 2008 | JR179 | BIO6-AGT-2B |  | -71.179 | -109.894 | 998 |
| Ophionotus | victoriae | DSOPH2191 |  | Amundsen Sea | 64 | KY048228 | 2008 | JR179 | BIO6-AGT-2B |  | -71.179 | -109.894 | 998 |
| Ophionotus | victoriae | DSOPH2199 |  | Amundsen Sea | 64 | KY048228 | 2008 | JR179 | BIO6-AGT-2A |  | -71.175 | -109.863 | 1080 |
| Ophionotus | victoriae | DSOPH2229 |  | Amundsen Sea | 64 | KY048228 | 2008 | JR179 | BIO6-AGT-2A |  | -71.175 | -109.863 | 1080 |
| Ophionotus | victoriae | DSOPH2255 |  | Amundsen Sea | 64 | KY048228 | 2008 | JR179 | BIO6-AGT-2C |  | -71.182 | -109.926 | 987 |
| Ophionotus | victoriae | DSOPH2273 |  | Amundsen Sea | 64 | KY048228 | 2008 | JR179 | BIO6-AGT-2C |  | -71.182 | -109.926 | 987 |
| Ophionotus | victoriae | DSOPH2566 |  | Amundsen Sea | 64 | KY048237 | 2008 | JR179 | BIO4-AGT-2A |  | -74.479 | -104.237 | 1208 |
| Ophionotus | victoriae | DSOPH2733 |  | Amundsen Sea | 64 | KY048228 | 2008 | JR179 | BIO6-AGT-2B |  | -71.179 | -109.894 | 998 |
| Ophionotus | victoriae | DSOPH2736 |  | Amundsen Sea | 64 | KY048228 | 2008 | JR179 | BIO6-AGT-2B |  | -71.179 | -109.894 | 998 |
| Ophionotus | victoriae | DSOPH2193 |  | Amundsen Sea | 65 | KY048229 | 2008 | JR179 | BIO6-AGT-2B |  | -71.179 | -109.894 | 998 |
| Ophionotus | victoriae | DSOPH2206 |  | Amundsen Sea | 65 | KY048229 | 2008 | JR179 | BIO6-AGT-2A |  | -71.175 | -109.863 | 1080 |
| Ophionotus | victoriae | DSOPH2208 |  | Amundsen Sea | 65 | KY048229 | 2008 | JR179 | BIO6-AGT-2A |  | -71.175 | -109.863 | 1080 |
| Ophionotus | victoriae | DSOPH2211 |  | Amundsen Sea | 65 | KY048229 | 2008 | JR179 | BIO6-AGT-2A |  | -71.175 | -109.863 | 1080 |
| Ophionotus | victoriae | DSOPH2216 |  | Amundsen Sea | 65 | KY048229 | 2008 | JR179 | BIO6-AGT-2A |  | -71.175 | -109.863 | 1080 |
| Ophionotus | victoriae | DSOPH2230 |  | Amundsen Sea | 65 | KY048229 | 2008 | JR179 | BIO6-AGT-2A |  | -71.175 | -109.863 | 1080 |
| Ophionotus | victoriae | DSOPH2252 |  | Amundsen Sea | 65 | KY048229 | 2008 | JR179 | BIO6-AGT-2C |  | -71.182 | -109.926 | 987 |
| Ophionotus | victoriae | DSOPH2259 |  | Amundsen Sea | 65 | KY048229 | 2008 | JR179 | BIO6-AGT-2C |  | -71.182 | -109.926 | 987 |
| Ophionotus | victoriae | DSOPH2272 |  | Amundsen Sea | 65 | KY048229 | 2008 | JR179 | BIO6-AGT-2C |  | -71.182 | -109.926 | 987 |
| Ophionotus | victoriae | DSOPH2676 |  | Amundsen Sea | 65 | KY048229 | 2008 | JR179 | BIO4-AGT-2B |  | -74.48 | -104.255 | 1163 |
| Ophionotus | victoriae | DSOPH2738 |  | Amundsen Sea | 65 | KY048229 | 2008 | JR179 | BIO6-AGT-2B |  | -71.179 | -109.894 | 998 |
| Ophionotus | victoriae | DSOPH2195 |  | Amundsen Sea | 66 | KY048230 | 2008 | JR179 | BIO6-AGT-2B |  | -71.179 | -109.894 | 998 |
| Ophionotus | victoriae | DSOPH2213 |  | Amundsen Sea | 66 | KY048230 | 2008 | JR179 | BIO6-AGT-2A |  | -71.175 | -109.863 | 1080 |
| Ophionotus | victoriae | DSOPH2253 |  | Amundsen Sea | 66 | KY048230 | 2008 | JR179 | BIO6-AGT-2C |  | -71.182 | -109.926 | 987 |
| Ophionotus | victoriae | DSOPH2260 |  | Amundsen Sea | 66 | KY048230 | 2008 | JR179 | BIO6-AGT-2C |  | -71.182 | -109.926 | 987 |
| Ophionotus | victoriae | DSOPH2264 |  | Amundsen Sea | 66 | KY048230 | 2008 | JR179 | BIO6-AGT-2C |  | -71.182 | -109.926 | 987 |
| Ophionotus | victoriae | DSOPH2359 |  | Amundsen Sea | 66 | KY048230 | 2008 | JR179 | BIO6-AGT-1A |  | -71.146 | -109.971 | 1531 |
| Ophionotus | victoriae | DSOPH2680 |  | Amundsen Sea | 66 | KY048230 | 2008 | JR179 | BIO4-AGT-2C |  | -74.477 | -104.257 | 1151 |
| Ophionotus | victoriae | DSOPH2730 |  | Amundsen Sea | 66 | KY048230 | 2008 | JR179 | BIO6-AGT-2B |  | -71.179 | -109.894 | 998 |
| Ophionotus | victoriae | DSOPH2732 |  | Amundsen Sea | 66 | KY048230 | 2008 | JR179 | BIO6-AGT-2B |  | -71.179 | -109.894 | 998 |
| Ophionotus | victoriae | DSOPH2747 |  | Amundsen Sea | 66 | KY048230 | 2008 | JR179 | BIO6-AGT-2B |  | -71.179 | -109.894 | 998 |
| Ophionotus | victoriae | DSOPH2753 |  | Amundsen Sea | 66 | KY048230 | 2008 | JR179 | BIO6-AGT-2B |  | -71.179 | -109.894 | 998 |
| Ophionotus | victoriae | DSOPH678 |  | Amundsen Sea | 66 | KY048230 | 2008 | JR179 | BIO5-AGT-3C |  | -73.986 | -107.39 | 542 |
| Ophionotus | victoriae | DSOPH2200 |  | Amundsen Sea | 67 | KY048232 | 2008 | JR179 | BIO6-AGT-2A |  | -71.175 | -109.863 | 1080 |
| Ophionotus | victoriae | DSOPH2276 |  | Amundsen Sea | 67 | KY048232 | 2008 | JR179 | BIO6-AGT-2C |  | -71.182 | -109.926 | 987 |
| Ophionotus | victoriae | DSOPH2201 |  | Amundsen Sea | 68 | KY048233 | 2008 | JR179 | BIO6-AGT-2A |  | -71.175 | -109.863 | 1080 |
| Ophionotus | victoriae | DSOPH2202 |  | Amundsen Sea | 68 | KY048233 | 2008 | JR179 | BIO6-AGT-2A |  | -71.175 | -109.863 | 1080 |
| Ophionotus | victoriae | DSOPH2209 |  | Amundsen Sea | 68 | KY048233 | 2008 | JR179 | BIO6-AGT-2A |  | -71.175 | -109.863 | 1080 |
| Ophionotus | victoriae | DSOPH2731 |  | Amundsen Sea | 68 | KY048233 | 2008 | JR179 | BIO6-AGT-2B |  | -71.179 | -109.894 | 998 |
| Ophionotus | victoriae | DSOPH2752 |  | Amundsen Sea | 68 | KY048233 | 2008 | JR179 | BIO6-AGT-2B |  | -71.179 | -109.894 | 998 |
| Ophionotus | victoriae | DSOPH2205 |  | Amundsen Sea | 69 | KY048235 | 2008 | JR179 | BIO6-AGT-2A |  | -71.175 | -109.863 | 1080 |
| Ophionotus | victoriae | DSOPH2275 |  | Amundsen Sea | 70 | KY048236 | 2008 | JR179 | BIO6-AGT-2C |  | -71.182 | -109.926 | 987 |
| Ophionotus | victoriae | DSOPH2567 |  | Amundsen Sea | 71 | KY048238 | 2008 | JR179 | BIO4-AGT-2B |  | -74.48 | -104.255 | 1163 |
| Ophionotus | victoriae | DSOPH2729 |  | Amundsen Sea | 72 | KY048239 | 2008 | JR179 | BIO6-AGT-2B |  | -71.179 | -109.894 | 998 |
| Ophionotus | victoriae | DSOPH2742 |  | Amundsen Sea | 73 | KY048240 | 2008 | JR179 | BIO6-AGT-2B |  | -71.179 | -109.894 | 998 |
| Ophionotus | victoriae | N0098 | NIWA84670 | Balleny Islands | 1 |  | 2006 | TAN0602 |  | 448 | -66.556833 | 162.569833 | 85 |
| Ophionotus | victoriae | 81991A | NIWA81991 | Balleny Islands | 24 |  | 2001 | TAN0102 |  | K0807 | -67.56800079 | 164.9583282 | 148 |
| Ophionotus | victoriae | 81991B | NIWA81991 | Balleny Islands | 24 |  | 2001 | TAN0102 |  | K0807 | -67.56800079 | 164.9583282 | 148 |
| Ophionotus | victoriae | 94857A | NIWA94857 | Balleny Islands | 24 |  | 2004 | TAN0402 |  | 233 | -67.41783142 | 163.9154968 | 227 |
| Ophionotus | victoriae | 94857B | NIWA94857 | Balleny Islands | 24 |  | 2004 | TAN0402 |  | 233 | -67.41783142 | 163.9154968 | 227 |
| Ophionotus | victoriae | N0081 | NIWA84670 | Balleny Islands | 24 |  | 2006 | TAN0602 |  | 448 | -66.556833 | 162.569833 | 85 |
| Ophionotus | victoriae | N0083 | NIWA84670 | Balleny Islands | 24 |  | 2006 | TAN0602 |  | 448 | -66.556833 | 162.569833 | 85 |
| Ophionotus | victoriae | N0084 | NIWA84670 | Balleny Islands | 24 |  | 2006 | TAN0602 |  | 448 | -66.556833 | 162.569833 | 85 |
| Ophionotus | victoriae | N0085 | NIWA84670 | Balleny Islands | 24 |  | 2006 | TAN0602 |  | 448 | -66.556833 | 162.569833 | 85 |
| Ophionotus | victoriae | N0086 | NIWA84670 | Balleny Islands | 24 |  | 2006 | TAN0602 |  | 448 | -66.556833 | 162.569833 | 85 |
| Ophionotus | victoriae | N0088 | NIWA84670 | Balleny Islands | 24 |  | 2006 | TAN0602 |  | 448 | -66.556833 | 162.569833 | 85 |
| Ophionotus | victoriae | N0089 | NIWA84670 | Balleny Islands | 24 |  | 2006 | TAN0602 |  | 448 | -66.556833 | 162.569833 | 85 |
| Ophionotus | victoriae | N0090 | NIWA84670 | Balleny Islands | 24 |  | 2006 | TAN0602 |  | 448 | -66.556833 | 162.569833 | 85 |
| Ophionotus | victoriae | N0091 | NIWA84670 | Balleny Islands | 24 |  | 2006 | TAN0602 |  | 448 | -66.556833 | 162.569833 | 85 |
| Ophionotus | victoriae | N0092 | NIWA84670 | Balleny Islands | 24 |  | 2006 | TAN0602 |  | 448 | -66.556833 | 162.569833 | 85 |
| Ophionotus | victoriae | N0093 | NIWA84670 | Balleny Islands | 24 |  | 2006 | TAN0602 |  | 448 | -66.556833 | 162.569833 | 85 |
| Ophionotus | victoriae | N0095 | NIWA84670 | Balleny Islands | 24 |  | 2006 | TAN0602 |  | 448 | -66.556833 | 162.569833 | 85 |
| Ophionotus | victoriae | N0096 | NIWA84670 | Balleny Islands | 24 |  | 2006 | TAN0602 |  | 448 | -66.556833 | 162.569833 | 85 |
| Ophionotus | victoriae | N0097 | NIWA84670 | Balleny Islands | 24 |  | 2006 | TAN0602 |  | 448 | -66.556833 | 162.569833 | 85 |
| Ophionotus | victoriae | WAMZ44963 | WAMZ44963 | Balleny Islands | 24 |  | 2017 | ACE 2016/17 | 46 | 1209 | -66.1742 | 162.2029 | 350 |
| Ophionotus | victoriae | WAMZ44964 | WAMZ44964 | Balleny Islands | 24 |  | 2017 | ACE 2016/17 | 46 | 1209 | -66.1742 | 162.2029 | 350 |
| Ophionotus | victoriae | WAMZ44966 | WAMZ44966 | Balleny Islands | 24 |  | 2017 | ACE 2016/17 | 46 | 1209 | -66.1742 | 162.2029 | 350 |
| Ophionotus | victoriae | WAMZ44967 | WAMZ44967 | Balleny Islands | 24 |  | 2017 | ACE 2016/17 | 46 | 1209 | -66.1742 | 162.2029 | 350 |
| Ophionotus | victoriae | WAMZ44968 | WAMZ44968 | Balleny Islands | 24 |  | 2017 | ACE 2016/17 | 46 | 1209 | -66.1742 | 162.2029 | 350 |
| Ophionotus | victoriae | WAMZ44970 | WAMZ44970 | Balleny Islands | 24 |  | 2017 | ACE 2016/17 | 46 | 1209 | -66.1742 | 162.2029 | 350 |
| Ophionotus | victoriae | WAMZ44971 | WAMZ44971 | Balleny Islands | 24 |  | 2017 | ACE 2016/17 | 46 | 1209 | -66.1742 | 162.2029 | 350 |
| Ophionotus | victoriae | WAMZ44972 | WAMZ44972 | Balleny Islands | 24 |  | 2017 | ACE 2016/17 | 46 | 1209 | -66.1742 | 162.2029 | 350 |
| Ophionotus | victoriae | WAMZ44973 | WAMZ44973 | Balleny Islands | 24 |  | 2017 | ACE 2016/17 | 46 | 1209 | -66.1742 | 162.2029 | 350 |
| Ophionotus | victoriae | WAMZ44974 | WAMZ44974 | Balleny Islands | 24 |  | 2017 | ACE 2016/17 | 46 | 1209 | -66.1742 | 162.2029 | 350 |
| Ophionotus | victoriae | WAMZ44975 | WAMZ44975 | Balleny Islands | 24 |  | 2017 | ACE 2016/17 | 46 | 1209 | -66.1742 | 162.2029 | 350 |
| Ophionotus | victoriae | WAMZ44976 | WAMZ44976 | Balleny Islands | 24 |  | 2017 | ACE 2016/17 | 46 | 1209 | -66.1742 | 162.2029 | 350 |
| Ophionotus | victoriae | WAMZ44977 | WAMZ44977 | Balleny Islands | 24 |  | 2017 | ACE 2016/17 | 46 | 1209 | -66.1742 | 162.2029 | 350 |
| Ophionotus | victoriae | WAMZ44978 | WAMZ44978 | Balleny Islands | 24 |  | 2017 | ACE 2016/17 | 46 | 1209 | -66.1742 | 162.2029 | 350 |
| Ophionotus | victoriae | WAMZ44979 | WAMZ44979 | Balleny Islands | 24 |  | 2017 | ACE 2016/17 | 46 | 1209 | -66.1742 | 162.2029 | 350 |
| Ophionotus | victoriae | WAMZ44980 | WAMZ44980 | Balleny Islands | 24 |  | 2017 | ACE 2016/17 | 46 | 1209 | -66.1742 | 162.2029 | 350 |
| Ophionotus | victoriae | WAMZ44982 | WAMZ44982 | Balleny Islands | 24 |  | 2017 | ACE 2016/17 | 46 | 1209 | -66.1742 | 162.2029 | 350 |
| Ophionotus | victoriae | WAMZ44983 | WAMZ44983 | Balleny Islands | 24 |  | 2017 | ACE 2016/17 | 46 | 1209 | -66.1742 | 162.2029 | 350 |
| Ophionotus | victoriae | WAMZ44984 | WAMZ44984 | Balleny Islands | 24 |  | 2017 | ACE 2016/17 | 46 | 1209 | -66.1742 | 162.2029 | 350 |
| Ophionotus | victoriae | WAMZ44969 | WAMZ44969 | Balleny Islands | 93 |  | 2017 | ACE 2016/17 | 46 | 1209 | -66.1742 | 162.2029 | 350 |
| Ophionotus | victoriae | N0078 | NIWA84670 | Balleny Islands | 96 |  | 2006 | TAN0602 |  | 448 | -66.556833 | 162.569833 | 85 |
| Ophionotus | victoriae | WAMZ44965 | WAMZ44965 | Balleny Islands | 96 |  | 2017 | ACE 2016/17 | 46 | 1209 | -66.1742 | 162.2029 | 350 |
| Ophionotus | victoriae | N0079 | NIWA84670 | Balleny Islands | 97 |  | 2006 | TAN0602 |  | 448 | -66.556833 | 162.569833 | 85 |
| Ophionotus | victoriae | N0080 | NIWA84670 | Balleny Islands | 98 |  | 2006 | TAN0602 |  | 448 | -66.556833 | 162.569833 | 85 |
| Ophionotus | victoriae | N0082 | NIWA84670 | Balleny Islands | 99 |  | 2006 | TAN0602 |  | 448 | -66.556833 | 162.569833 | 85 |
| Ophionotus | victoriae | N0087 | NIWA84670 | Balleny Islands | 100 |  | 2006 | TAN0602 |  | 448 | -66.556833 | 162.569833 | 85 |
| Ophionotus | victoriae | N0094 | NIWA84670 | Balleny Islands | 101 |  | 2006 | TAN0602 |  | 448 | -66.556833 | 162.569833 | 85 |
| Ophionotus | victoriae | WAMZ44962 | WAMZ44962 | Balleny Islands | 156 |  | 2017 | ACE 2016/17 | 46 | 1209 | -66.1742 | 162.2029 | 350 |
| Ophionotus | victoriae | WAMZ44981 | WAMZ44981 | Balleny Islands | 157 |  | 2017 | ACE 2016/17 | 46 | 1209 | -66.1742 | 162.2029 | 350 |
| Ophionotus | victoriae | Op531_3E_11 |  | Bellingshausen Sea | 1 | FJ917337 | 2013 | NBP-12-10 | 3 |  | -71.699 | -93.693667 | 670 |
| Ophionotus | victoriae | Op531_3E_6 |  | Bellingshausen Sea | 62 | KY048226 | 2013 | NBP-12-10 | 3 |  | -71.699 | -93.693667 | 670 |
| Ophionotus | victoriae | Op531_3E_13 |  | Bellingshausen Sea | 64 | KY048228 | 2013 | NBP-12-10 | 3 |  | -71.699 | -93.693667 | 670 |
| Ophionotus | victoriae | Op531_3E_9 |  | Bellingshausen Sea | 64 | KY048228 | 2013 | NBP-12-10 | 3 |  | -71.699 | -93.693667 | 670 |
| Ophionotus | victoriae | Op531_3E_4 |  | Bellingshausen Sea | 72 | KY048239 | 2013 | NBP-12-10 | 3 |  | -71.699 | -93.693667 | 670 |
| Ophionotus | victoriae | Op531_3E_8 |  | Bellingshausen Sea | 72 | KY048239 | 2013 | NBP-12-10 | 3 |  | -71.699 | -93.693667 | 670 |
| Ophionotus | victoriae | Op531_3E_10 |  | Bellingshausen Sea | 88 | KY048258 | 2013 | NBP-12-10 | 3 |  | -71.699 | -93.693667 | 670 |
| Ophionotus | victoriae | OP531_3E_12 |  | Bellingshausen Sea | 89 | KY048259 | 2013 | NBP-12-10 | 3 |  | -71.699 | -93.693667 | 670 |
| Ophionotus | victoriae | Op531_3E_5 |  | Bellingshausen Sea | 90 | KY048260 | 2013 | NBP-12-10 | 3 |  | -71.699 | -93.693667 | 670 |
| Ophionotus | victoriae | 196.1E.10 |  | Bouvet Island | 4 | FJ917313 | 2006 | LMG-04-14 | 50 |  | -56.00683 | 2.6013889 | 648 |
| Ophionotus | victoriae | WAMZ44952 | WAMZ44952 | Bouvet Island | 4 |  | 2017 | ACE 2016/17 | 98 | 2765 | -54.419 | -3.4935 | 300 |
| Ophionotus | victoriae | WAMZ44954 | WAMZ44954 | Bouvet Island | 4 |  | 2017 | ACE 2016/17 | 98 | 2765 | -54.419 | -3.4935 | 300 |
| Ophionotus | victoriae | 177.1E.01 |  | Bouvet Island | 28 | FJ917324 | 2004 | LMG-04-14 | 58 |  | -54.816665 | -3.5 | 169 |
| Ophionotus | victoriae | 177.1E.05 |  | Bouvet Island | 28 | FJ917324 | 2004 | LMG-04-14 | 58 |  | -54.816665 | -3.5 | 169 |
| Ophionotus | victoriae | 196.1E.07 |  | Bouvet Island | 28 | FJ917324 | 2006 | LMG-04-14 | 50 |  | -56.00683 | 2.6013889 | 684 |
| Ophionotus | victoriae | DSOPH3146 |  | Bouvet Island | 28 | FJ917324 | 2011 | Polarstern ANT-XXVII/3 | PS77_312-4 |  | -54.481 | 3.189 | 300 |
| Ophionotus | victoriae | SA6 (haplotype ID) |  | Bouvet Island | 29 | FJ917325 | 2004 | Nathaniel B. Palmer |  |  | -54.816665 | -3.5 | 169 |
| Ophionotus | victoriae | WAMZ44951 | WAMZ44951 | Bouvet Island | 29 |  | 2017 | ACE 2016/17 | 98 | 2765 | -54.419 | -3.4935 | 300 |
| Ophionotus | victoriae | WAMZ44953 | WAMZ44953 | Bouvet Island | 29 |  | 2017 | ACE 2016/17 | 98 | 2765 | -54.419 | -3.4935 | 300 |
| Ophionotus | victoriae | WAMZ44947 | WAMZ44947 | Bouvet Island | 30 |  | 2017 | ACE 2016/17 | 98 | 2765 | -54.419 | -3.4935 | 300 |
| Ophionotus | victoriae | WAMZ44948 | WAMZ44948 | Bouvet Island | 30 |  | 2017 | ACE 2016/17 | 98 | 2765 | -54.419 | -3.4935 | 300 |
| Ophionotus | victoriae | WAMZ44949 | WAMZ44949 | Bouvet Island | 30 |  | 2017 | ACE 2016/17 | 98 | 2765 | -54.419 | -3.4935 | 300 |
| Ophionotus | victoriae | WAMZ44950 | WAMZ44950 | Bouvet Island | 30 |  | 2017 | ACE 2016/17 | 98 | 2765 | -54.419 | -3.4935 | 300 |
| Ophionotus | victoriae | WAMZ44957 | WAMZ44957 | Bouvet Island | 30 |  | 2017 | ACE 2016/17 | 98 | 2765 | -54.419 | -3.4935 | 300 |
| Ophionotus | victoriae | WAMZ44958 | WAMZ44958 | Bouvet Island | 30 |  | 2017 | ACE 2016/17 | 98 | 2765 | -54.419 | -3.4935 | 300 |
| Ophionotus | victoriae | WAMZ44961 | WAMZ44961 | Bouvet Island | 30 |  | 2017 | ACE 2016/17 | 98 | 2765 | -54.419 | -3.4935 | 300 |
| Ophionotus | victoriae | 196.1E.03 |  | Bouvet Island | 37 | FJ917336 | 2006 | LMG-04-14 | 50 |  | -56.00683 | 2.6013889 | 548 |
| Ophionotus | victoriae | DSOPH3044 |  | Bouvet Island | 77 | KY048245 | 2011 | Polarstern ANT-XXVII/3 | PS77_312-2 |  | -54.47 | 3.185 | 296 |
| Ophionotus | victoriae | DSOPH3158 |  | Bouvet Island | 80 | KY048249 | 2011 | Polarstern ANT-XXVII/3 | PS77_312-3 |  | -54.502 | 3.225 | 264 |
| Ophionotus | victoriae | DSOPH3159 |  | Bouvet Island | 81 | KY048250 | 2011 | Polarstern ANT-XXVII/3 | PS77_312-3 |  | -54.502 | 3.225 | 264 |
| Ophionotus | victoriae | DSOPH3226 |  | Bouvet Island | 82 | KY048251 | 2011 | Polarstern ANT-XXVII/3 | PS77_312-2 |  | -54.47 | 3.185 | 296 |
| Ophionotus | victoriae | WAMZ44955 | WAMZ44955 | Bouvet Island | 152 |  | 2017 | ACE 2016/17 | 98 | 2765 | -54.419 | -3.4935 | 300 |
| Ophionotus | victoriae | WAMZ44956 | WAMZ44956 | Bouvet Island | 153 |  | 2017 | ACE 2016/17 | 98 | 2765 | -54.419 | -3.4935 | 300 |
| Ophionotus | victoriae | WAMZ44959 | WAMZ44959 | Bouvet Island | 154 |  | 2017 | ACE 2016/17 | 98 | 2765 | -54.419 | -3.4935 | 300 |
| Ophionotus | victoriae | WAMZ44960 | WAMZ44960 | Bouvet Island | 155 |  | 2017 | ACE 2016/17 | 98 | 2765 | -54.419 | -3.4935 | 300 |
| Ophionotus | victoriae | 177.1E.02 |  | Bouvet Island | 29 | FJ917327 | 2004 | LMG-04-14 | 58 |  | -54.816665 | -3.5 | 169 |
| Ophionotus | victoriae | 177.1E.06 |  | Bouvet Island | 29 | FJ917327 | 2004 | LMG-04-14 | 58 |  | -54.816665 | -3.5 | 169 |
| Ophionotus | victoriae | 177.1E.09 |  | Bouvet Island | 29 | FJ917327 | 2004 | LMG-04-14 | 58 |  | -54.816665 | -3.5 | 169 |
| Ophionotus | victoriae | 177.1E.11 |  | Bouvet Island | 29 | FJ917327 | 2004 | LMG-04-14 | 58 |  | -54.816665 | -3.5 | 169 |
| Ophionotus | victoriae | DSOPH3144 |  | Bouvet Island | 29 | FJ917327 | 2001 | Polarstern ANT-XXVII/3 | PS77_312-4 |  | -54.481 | 3.189 | 300 |
| Ophionotus | victoriae | 177.1E.04 |  | Bouvet Island | 30 | FJ917326 | 2004 | LMG-04-14 | 58 |  | -54.816665 | -3.5 | 169 |
| Ophionotus | victoriae | 177.1E.07 |  | Bouvet Island | 30 | FJ917326 | 2004 | LMG-04-14 | 58 |  | -54.816665 | -3.5 | 169 |
| Ophionotus | victoriae | 177.1E.10 |  | Bouvet Island | 30 | FJ917326 | 2004 | LMG-04-14 | 58 |  | -54.816665 | -3.5 | 169 |
| Ophionotus | victoriae | 177.1E.12 |  | Bouvet Island | 30 | FJ917326 | 2004 | LMG-04-14 | 58 |  | -54.816665 | -3.5 | 169 |
| Ophionotus | victoriae | 196.1E.01 |  | Bouvet Island | 30 | FJ917326 | 2004 | LMG-04-14 | 50 |  | -56.00683 | 2.6013889 | 648 |
| Ophionotus | victoriae | 196.1E.04 |  | Bouvet Island | 30 | FJ917326 | 2004 | LMG-04-14 | 50 |  | -56.00683 | 2.6013889 | 648 |
| Ophionotus | victoriae | 196.1E.05 |  | Bouvet Island | 30 | FJ917326 | 2004 | LMG-04-14 | 50 |  | -56.00683 | 2.6013889 | 648 |
| Ophionotus | victoriae | 196.1E.06 |  | Bouvet Island | 30 | FJ917326 | 2004 | LMG-04-14 | 50 |  | -56.00683 | 2.6013889 | 648 |
| Ophionotus | victoriae | 196.1E.08 |  | Bouvet Island | 30 | FJ917326 | 2004 | LMG-04-14 | 50 |  | -56.00683 | 2.6013889 | 648 |
| Ophionotus | victoriae | DSOPH3043 |  | Bouvet Island | 30 | FJ917326 | 2011 | Polarstern ANT-XXVII/3 | PS77_312-2 |  | -54.47 | 3.185 | 296 |
| Ophionotus | victoriae | DSOPH3045 |  | Bouvet Island | 30 | FJ917326 | 2011 | Polarstern ANT-XXVII/3 | PS77_312-2 |  | -54.47 | 3.185 | 296 |
| Ophionotus | victoriae | DSOPH3046 |  | Bouvet Island | 30 | FJ917326 | 2011 | Polarstern ANT-XXVII/3 | PS77_312-2 |  | -54.47 | 3.185 | 296 |
| Ophionotus | victoriae | DSOPH3145 |  | Bouvet Island | 30 | FJ917326 | 2011 | Polarstern ANT-XXVII/3 | PS77_312-4 |  | -54.481 | 3.189 | 300 |
| Ophionotus | victoriae | DSOPH3147 |  | Bouvet Island | 30 | FJ917326 | 2011 | Polarstern ANT-XXVII/3 | PS77_312-4 |  | -54.481 | 3.189 | 300 |
| Ophionotus | victoriae | DSOPH3157 |  | Bouvet Island | 30 | FJ917326 | 2011 | Polarstern ANT-XXVII/3 | PS77_312-3 |  | -54.502 | 3.225 | 264 |
| Ophionotus | victoriae | DSOPH3160 |  | Bouvet Island | 30 | FJ917326 | 2011 | Polarstern ANT-XXVII/3 | PS77_312-3 |  | -54.502 | 3.225 | 264 |
| Ophionotus | victoriae | SIOBICP00524B | SIOBICE5524 | Bransfield Strait | 1 |  | 2012 | Polarstern ANT-XXVIII/4 |  | 79279 | -62.278167 | -55.832667 | 324 |
| Ophionotus | victoriae | SIOBICP00524I | SIOBICE5524 | Bransfield Strait | 1 |  | 2012 | Polarstern ANT-XXVIII/4 |  | 79279 | -62.278167 | -55.832667 | 324 |
| Ophionotus | victoriae | E82.2C.01 |  | Bransfield Strait | 1 | FJ917339 | 2004 | LMG-04-14 | 47 |  | -62.850361 | -59.45995 | 900 |
| Ophionotus | victoriae | E82.2C.02 |  | Bransfield Strait | 1 | FJ917339 | 2004 | LMG-04-14 | 47 |  | -62.850361 | -59.45995 | 900 |
| Ophionotus | victoriae | E82.2C.04 |  | Bransfield Strait | 1 | FJ917337 | 2006 | LMG-04-14 | 47 |  | -62.850361 | -59.45995 | 900 |
| Ophionotus | victoriae | E82.2C.05 |  | Bransfield Strait | 1 | FJ917337 | 2006 | LMG-04-14 | 47 |  | -62.850361 | -59.45995 | 900 |
| Ophionotus | victoriae | Op877_2E_1 |  | Bransfield Strait | 1 | FJ917339 | 2013 | LMG-13-12 | 4 |  | -62.995875 | -58.598617 | 320 |
| Ophionotus | victoriae | Op877_2E_3 |  | Bransfield Strait | 1 | FJ917337 | 2013 | LMG-13-12 | 4 |  | -62.995875 | -58.598617 | 320 |
| Ophionotus | victoriae | Op877_2E_7 |  | Bransfield Strait | 1 | FJ917337 | 2013 | LMG-13-12 | 4 |  | -62.995875 | -58.598617 | 320 |
| Ophionotus | victoriae | Op877_2E_8 |  | Bransfield Strait | 1 | FJ917339 | 2013 | LMG-13-12 | 4 |  | -62.995875 | -58.598617 | 320 |
| Ophionotus | victoriae | Op877_2E_9 |  | Bransfield Strait | 1 | FJ917337 | 2013 | LMG-13-12 | 4 |  | -62.995875 | -58.598617 | 320 |
| Ophionotus | victoriae | Op877_2E_5 |  | Bransfield Strait | 3 | FJ917340 | 2013 | LMG-13-12 | 4 |  | -62.995875 | -58.598617 | 320 |
| Ophionotus | victoriae | 92.10C |  | Bransfield Strait | 4 | FJ917313 | 2004 | LMG-04-14 | 51 |  | -63.383694 | -60.05995 | 277 |
| Ophionotus | victoriae | SIOBICP00496A | SIOBICE5492 | Bransfield Strait | 4 |  | 2012 | Polarstern ANT-XXVIII/4 |  | 79273 | -62.367333 | -55.960833 | 349 |
| Ophionotus | victoriae | SIOBICP00496R | SIOBICE5492 | Bransfield Strait | 4 |  | 2012 | Polarstern ANT-XXVIII/4 |  | 79273 | -62.367333 | -55.960833 | 349 |
| Ophionotus | victoriae | 92.16C |  | Bransfield Strait | 5 | FJ917320 | 2004 | LMG-04-14 | 51 |  | -63.383694 | -60.05995 | 277 |
| Ophionotus | victoriae | SIOBICP00496B | SIOBICE5492 | Bransfield Strait | 5 |  | 2012 | Polarstern ANT-XXVIII/4 |  | 79273 | -62.367333 | -55.960833 | 349 |
| Ophionotus | victoriae | SIOBICP00496D | SIOBICE5492 | Bransfield Strait | 5 |  | 2012 | Polarstern ANT-XXVIII/4 |  | 79273 | -62.367333 | -55.960833 | 349 |
| Ophionotus | victoriae | SIOBICP00496F | SIOBICE5492 | Bransfield Strait | 5 |  | 2012 | Polarstern ANT-XXVIII/4 |  | 79273 | -62.367333 | -55.960833 | 349 |
| Ophionotus | victoriae | SIOBICP00496H | SIOBICE5492 | Bransfield Strait | 5 |  | 2012 | Polarstern ANT-XXVIII/4 |  | 79273 | -62.367333 | -55.960833 | 349 |
| Ophionotus | victoriae | SIOBICP00496K | SIOBICE5492 | Bransfield Strait | 5 |  | 2012 | Polarstern ANT-XXVIII/4 |  | 79273 | -62.367333 | -55.960833 | 349 |
| Ophionotus | victoriae | SIOBICP00496N | SIOBICE5492 | Bransfield Strait | 5 |  | 2012 | Polarstern ANT-XXVIII/4 |  | 79273 | -62.367333 | -55.960833 | 349 |
| Ophionotus | victoriae | SIOBICP00496O | SIOBICE5492 | Bransfield Strait | 5 |  | 2012 | Polarstern ANT-XXVIII/4 |  | 79273 | -62.367333 | -55.960833 | 349 |
| Ophionotus | victoriae | SIOBICP00496T | SIOBICE5492 | Bransfield Strait | 5 |  | 2012 | Polarstern ANT-XXVIII/4 |  | 79273 | -62.367333 | -55.960833 | 349 |
| Ophionotus | victoriae | Op877_2E_10 |  | Bransfield Strait | 5 | FJ917320 | 2013 | LMG-13-12 | 4 |  | -62.995875 | -58.598617 | 320 |
| Ophionotus | victoriae | Op877_2E_4 |  | Bransfield Strait | 5 | FJ917320 | 2013 | LMG-13-12 | 4 |  | -62.995875 | -58.598617 | 320 |
| Ophionotus | victoriae | Op877_2E_6 |  | Bransfield Strait | 5 | FJ917320 | 2013 | LMG-13-12 | 4 |  | -62.995875 | -58.598617 | 320 |
| Ophionotus | victoriae | SIOBICS6316F | SIOBICE4770 | Bransfield Strait | 5 |  | 2011 | Scotia 2011 | BS2 | 89 | -63.34327 | -59.91016 | 213 |
| Ophionotus | victoriae | SIOBICS6338B | SIOBICE4781 | Bransfield Strait | 5 |  | 2011 | Scotia 2011 | BS2 | 90 | -63.28318 | -59.90272 | 290 |
| Ophionotus | victoriae | SIOBICS6338F | SIOBICE4781 | Bransfield Strait | 5 |  | 2011 | Scotia 2011 | BS2 | 90 | -63.28318 | -59.90272 | 290 |
| Ophionotus | victoriae | SIOBICS6760B | SIOBICE4777 | Bransfield Strait | 5 |  | 2011 | Scotia 2011 | BS1 | 87 | -62.753 | -57.322 | 292 |
| Ophionotus | victoriae | SIOBICS6760G | SIOBICE4777 | Bransfield Strait | 5 |  | 2011 | Scotia 2011 | BS1 | 87 | -62.753 | -57.322 | 292 |
| Ophionotus | victoriae | 92.14C |  | Bransfield Strait | 6 | FJ917309 | 2004 | LMG-04-14 | 51 |  | -63.383694 | -60.05995 | 277 |
| Ophionotus | victoriae | 92.9C |  | Bransfield Strait | 6 | FJ917309 | 2004 | LMG-04-14 | 51 |  | -63.383694 | -60.05995 | 277 |
| Ophionotus | victoriae | SIOBICP00496C | SIOBICE5492 | Bransfield Strait | 6 |  | 2012 | Polarstern ANT-XXVIII/4 |  | 79273 | -62.367333 | -55.960833 | 349 |
| Ophionotus | victoriae | SIOBICP00496G | SIOBICE5492 | Bransfield Strait | 6 |  | 2012 | Polarstern ANT-XXVIII/4 |  | 79273 | -62.367333 | -55.960833 | 349 |
| Ophionotus | victoriae | SIOBICP00496I | SIOBICE5492 | Bransfield Strait | 6 |  | 2012 | Polarstern ANT-XXVIII/4 |  | 79273 | -62.367333 | -55.960833 | 349 |
| Ophionotus | victoriae | SIOBICP00524A | SIOBICE5524 | Bransfield Strait | 6 |  | 2012 | Polarstern ANT-XXVIII/4 |  | 79279 | -62.278167 | -55.832667 | 324 |
| Ophionotus | victoriae | SIOBICS6316D | SIOBICE4770 | Bransfield Strait | 6 |  | 2011 | Scotia 2011 | BS2 | 89 | -63.34327 | -59.91016 | 213 |
| Ophionotus | victoriae | SIOBICS6316H | SIOBICE4770 | Bransfield Strait | 6 |  | 2011 | Scotia 2011 | BS2 | 89 | -63.34327 | -59.91016 | 213 |
| Ophionotus | victoriae | SIOBICS6338D | SIOBICE4781 | Bransfield Strait | 6 |  | 2011 | Scotia 2011 | BS2 | 90 | -63.28318 | -59.90272 | 290 |
| Ophionotus | victoriae | SIOBICS6760C | SIOBICE4777 | Bransfield Strait | 6 |  | 2011 | Scotia 2011 | BS1 | 87 | -62.753 | -57.322 | 292 |
| Ophionotus | victoriae | SIOBICS6905B | SIOBICE5162 | Bransfield Strait | 6 |  | 2011 | Scotia 2011 | BS1 | 86 | -62.86951 | -57.21682 | 247 |
| Ophionotus | victoriae | SIOBICS6905K | SIOBICE5162 | Bransfield Strait | 6 |  | 2011 | Scotia 2011 | BS1 | 86 | -62.86951 | -57.21682 | 247 |
| Ophionotus | victoriae | SIOBICP00496E | SIOBICE5492 | Bransfield Strait | 7 |  | 2012 | Polarstern ANT-XXVIII/4 |  | 79273 | -62.367333 | -55.960833 | 349 |
| Ophionotus | victoriae | SIOBICP00496J | SIOBICE5492 | Bransfield Strait | 7 |  | 2012 | Polarstern ANT-XXVIII/4 |  | 79273 | -62.367333 | -55.960833 | 349 |
| Ophionotus | victoriae | SIOBICP00496P | SIOBICE5492 | Bransfield Strait | 7 |  | 2012 | Polarstern ANT-XXVIII/4 |  | 79273 | -62.367333 | -55.960833 | 349 |
| Ophionotus | victoriae | SIOBICS6316G | SIOBICE4770 | Bransfield Strait | 7 |  | 2011 | Scotia 2011 | BS2 | 89 | -63.34327 | -59.91016 | 213 |
| Ophionotus | victoriae | SIOBICS6338C | SIOBICE4781 | Bransfield Strait | 7 |  | 2011 | Scotia 2011 | BS2 | 90 | -63.28318 | -59.90272 | 290 |
| Ophionotus | victoriae | SIOBICS6338G | SIOBICE4781 | Bransfield Strait | 7 |  | 2011 | Scotia 2011 | BS2 | 90 | -63.28318 | -59.90272 | 290 |
| Ophionotus | victoriae | SIOBICS6338J | SIOBICE4781 | Bransfield Strait | 7 |  | 2011 | Scotia 2011 | BS2 | 90 | -63.28318 | -59.90272 | 290 |
| Ophionotus | victoriae | SIOBICS6760F | SIOBICE4777 | Bransfield Strait | 7 |  | 2011 | Scotia 2011 | BS1 | 87 | -62.753 | -57.322 | 292 |
| Ophionotus | victoriae | SIOBICS6905A | SIOBICE5162 | Bransfield Strait | 7 |  | 2011 | Scotia 2011 | BS1 | 86 | -62.86951 | -57.21682 | 247 |
| Ophionotus | victoriae | SIOBICS6905D | SIOBICE5162 | Bransfield Strait | 7 |  | 2011 | Scotia 2011 | BS1 | 86 | -62.86951 | -57.21682 | 247 |
| Ophionotus | victoriae | SIOBICS6905E | SIOBICE5162 | Bransfield Strait | 7 |  | 2011 | Scotia 2011 | BS1 | 86 | -62.86951 | -57.21682 | 247 |
| Ophionotus | victoriae | SIOBICS6905L | SIOBICE5162 | Bransfield Strait | 7 |  | 2011 | Scotia 2011 | BS1 | 86 | -62.86951 | -57.21682 | 247 |
| Ophionotus | victoriae | 92.11C |  | Bransfield Strait | 8 | FJ917316 | 2004 | LMG-04-14 | 51 |  | -63.383694 | -60.05995 | 277 |
| Ophionotus | victoriae | 92.13C |  | Bransfield Strait | 8 | FJ917316 | 2004 | LMG-04-14 | 51 |  | -63.383694 | -60.05995 | 277 |
| Ophionotus | victoriae | 92.15C |  | Bransfield Strait | 8 | FJ917316 | 2004 | LMG-04-14 | 51 |  | -63.383694 | -60.05995 | 277 |
| Ophionotus | victoriae | 92.17C |  | Bransfield Strait | 8 | FJ917316 | 2004 | LMG-04-14 | 51 |  | -63.383694 | -60.05995 | 277 |
| Ophionotus | victoriae | 92.5C |  | Bransfield Strait | 8 | FJ917316 | 2004 | LMG-04-14 | 51 |  | -63.383694 | -60.05995 | 277 |
| Ophionotus | victoriae | 92.6C |  | Bransfield Strait | 8 | FJ917316 | 2004 | LMG-04-14 | 51 |  | -63.383694 | -60.05995 | 277 |
| Ophionotus | victoriae | SIOBICP00496L | SIOBICE5492 | Bransfield Strait | 8 |  | 2012 | Polarstern ANT-XXVIII/4 |  | 79273 | -62.367333 | -55.960833 | 349 |
| Ophionotus | victoriae | SIOBICP00496Q | SIOBICE5492 | Bransfield Strait | 8 |  | 2012 | Polarstern ANT-XXVIII/4 |  | 79273 | -62.367333 | -55.960833 | 349 |
| Ophionotus | victoriae | Op877_2E_2 |  | Bransfield Strait | 8 | FJ917316 | 2013 | LMG-13-12 | 4 |  | -62.995875 | -58.598617 | 320 |
| Ophionotus | victoriae | SIOBICS6316A | SIOBICE4770 | Bransfield Strait | 8 |  | 2011 | Scotia 2011 | BS2 | 89 | -63.34327 | -59.91016 | 213 |
| Ophionotus | victoriae | SIOBICS6316C | SIOBICE4770 | Bransfield Strait | 8 |  | 2011 | Scotia 2011 | BS2 | 89 | -63.34327 | -59.91016 | 213 |
| Ophionotus | victoriae | SIOBICS6338I | SIOBICE4781 | Bransfield Strait | 8 |  | 2011 | Scotia 2011 | BS2 | 90 | -63.28318 | -59.90272 | 290 |
| Ophionotus | victoriae | SIOBICS6760A | SIOBICE4777 | Bransfield Strait | 8 |  | 2011 | Scotia 2011 | BS1 | 87 | -62.753 | -57.322 | 292 |
| Ophionotus | victoriae | SIOBICP00496M | SIOBICE5492 | Bransfield Strait | 9 |  | 2012 | Polarstern ANT-XXVIII/4 |  | 79273 | -62.367333 | -55.960833 | 349 |
| Ophionotus | victoriae | SIOBICP00496S | SIOBICE5492 | Bransfield Strait | 10 |  | 2012 | Polarstern ANT-XXVIII/4 |  | 79273 | -62.367333 | -55.960833 | 349 |
| Ophionotus | victoriae | SIOBICP00524C | SIOBICE5524 | Bransfield Strait | 14 |  | 2012 | Polarstern ANT-XXVIII/4 |  | 79279 | -62.278167 | -55.832667 | 324 |
| Ophionotus | victoriae | SIOBICP00524D | SIOBICE5524 | Bransfield Strait | 14 |  | 2012 | Polarstern ANT-XXVIII/4 |  | 79279 | -62.278167 | -55.832667 | 324 |
| Ophionotus | victoriae | SIOBICP00524G | SIOBICE5524 | Bransfield Strait | 14 |  | 2012 | Polarstern ANT-XXVIII/4 |  | 79279 | -62.278167 | -55.832667 | 324 |
| Ophionotus | victoriae | SIOBICP00524E | SIOBICE5524 | Bransfield Strait | 15 |  | 2012 | Polarstern ANT-XXVIII/4 |  | 79279 | -62.278167 | -55.832667 | 324 |
| Ophionotus | victoriae | SIOBICP00524F | SIOBICE5524 | Bransfield Strait | 16 |  | 2012 | Polarstern ANT-XXVIII/4 |  | 79279 | -62.278167 | -55.832667 | 324 |
| Ophionotus | victoriae | SIOBICP00524H | SIOBICE5524 | Bransfield Strait | 17 |  | 2012 | Polarstern ANT-XXVIII/4 |  | 79279 | -62.278167 | -55.832667 | 324 |
| Ophionotus | victoriae | 92.3C |  | Bransfield Strait | 21 | FJ917314 | 2004 | LMG-04-14 | 51 |  | -63.383694 | -60.05995 | 900 |
| Ophionotus | victoriae | 92.4C |  | Bransfield Strait | 22 | FJ917315 | 2004 | LMG-04-14 | 51 |  | -63.383694 | -60.05995 | 900 |
| Ophionotus | victoriae | 92.7C |  | Bransfield Strait | 23 | FJ917317 | 2004 | LMG-04-14 | 51 |  | -63.383694 | -60.05995 | 277 |
| Ophionotus | victoriae | 92.8C |  | Bransfield Strait | 24 | FJ917318 | 2004 | LMG-04-14 | 51 |  | -63.383694 | -60.05995 | 277 |
| Ophionotus | victoriae | SIOBICS6316B | SIOBICE4770 | Bransfield Strait | 24 |  | 2011 | Scotia 2011 | BS2 | 89 | -63.34327 | -59.91016 | 213 |
| Ophionotus | victoriae | SIOBICS6760H | SIOBICE4777 | Bransfield Strait | 24 |  | 2011 | Scotia 2011 | BS1 | 87 | -62.753 | -57.322 | 292 |
| Ophionotus | victoriae | 92.12C |  | Bransfield Strait | 25 | FJ917319 | 2004 | LMG-04-14 | 51 |  | -63.383694 | -60.05995 | 277 |
| Ophionotus | victoriae | SIOBICS6338H | SIOBICE4781 | Bransfield Strait | 25 |  | 2011 | Scotia 2011 | BS2 | 90 | -63.28318 | -59.90272 | 290 |
| Ophionotus | victoriae | SIOBICS6905C | SIOBICE5162 | Bransfield Strait | 25 |  | 2011 | Scotia 2011 | BS1 | 86 | -62.86951 | -57.21682 | 247 |
| Ophionotus | victoriae | SIOBICS6905I | SIOBICE5162 | Bransfield Strait | 32 |  | 2011 | Scotia 2011 | BS1 | 86 | -62.86951 | -57.21682 | 247 |
| Ophionotus | victoriae | SIOBICS6760D | SIOBICE4777 | Bransfield Strait | 39 |  | 2011 | Scotia 2011 | BS1 | 87 | -62.753 | -57.322 | 292 |
| Ophionotus | victoriae | SIOBICS6905G | SIOBICE5162 | Bransfield Strait | 39 |  | 2011 | Scotia 2011 | BS1 | 86 | -62.86951 | -57.21682 | 247 |
| Ophionotus | victoriae | E82.2C.03 |  | Bransfield Strait | 47 | FJ917354 | 2006 | LMG-04-14 | 47 |  | -62.850361 | -59.45995 | 900 |
| Ophionotus | victoriae | SIOBICS6760E | SIOBICE4777 | Bransfield Strait | 84 |  | 2011 | Scotia 2011 | BS1 | 87 | -62.753 | -57.322 | 292 |
| Ophionotus | victoriae | SIOBICS6316E | SIOBICE4770 | Bransfield Strait | 138 |  | 2011 | Scotia 2011 | BS2 | 89 | -63.34327 | -59.91016 | 213 |
| Ophionotus | victoriae | SIOBICS6338A | SIOBICE4781 | Bransfield Strait | 139 |  | 2011 | Scotia 2011 | BS2 | 90 | -63.28318 | -59.90272 | 290 |
| Ophionotus | victoriae | SIOBICS6338E | SIOBICE4781 | Bransfield Strait | 140 |  | 2011 | Scotia 2011 | BS2 | 90 | -63.28318 | -59.90272 | 290 |
| Ophionotus | victoriae | SIOBICS6338K | SIOBICE4781 | Bransfield Strait | 141 |  | 2011 | Scotia 2011 | BS2 | 90 | -63.28318 | -59.90272 | 290 |
| Ophionotus | victoriae | SIOBICS6905H | SIOBICE5162 | Bransfield Strait | 142 |  | 2011 | Scotia 2011 | BS1 | 86 | -62.86951 | -57.21682 | 247 |
| Ophionotus | victoriae | SIOBICS6905J | SIOBICE5162 | Bransfield Strait | 143 |  | 2011 | Scotia 2011 | BS1 | 86 | -62.86951 | -57.21682 | 247 |
| Ophionotus | victoriae | PNG708 | PNG708 | Davis Sea | 1 |  | 2010 | AAD | Tressler2 | BTC15 | -64.5596915 | 95.31715 | 779 |
| Ophionotus | victoriae | PNG710 | PNG710 | Davis Sea | 1 |  | 2010 | AAD | Tressler2 | BTC15 | -64.5596915 | 95.31715 | 779 |
| Ophionotus | victoriae | WAMZ43206 | WAMZ43206 | Davis Sea | 68 |  | 2016 | AAD |  | 59 | -65.06666667 | 113.7783333 | 1331 |
| Ophionotus | victoriae | PNG703 | PNG703 | Davis Sea | 112 |  | 2010 | AAD | Tressler2 | BTC17 | -64.559508 | 95.3199835 | 758 |
| Ophionotus | victoriae | SIOBICS4306 | SIOBICE5221 | Discovery Bank | 32 |  | 2011 | Scotia 2011 | DB1 | 58 | -60.11104 | -34.82669 | 439 |
| Ophionotus | victoriae | SIOBICS4307 | SIOBICE5169 | Discovery Bank | 32 |  | 2011 | Scotia 2011 | DB1 | 58 | -60.11104 | -34.82669 | 439 |
| Ophionotus | victoriae | SIOBICS4309 | SIOBICE5168 | Discovery Bank | 32 |  | 2011 | Scotia 2011 | DB1 | 58 | -60.11104 | -34.82669 | 439 |
| Ophionotus | victoriae | SIOBICS4310 | SIOBICE5183 | Discovery Bank | 32 |  | 2011 | Scotia 2011 | DB1 | 58 | -60.11104 | -34.82669 | 439 |
| Ophionotus | victoriae | SIOBICS4311 | SIOBICE5239 | Discovery Bank | 32 |  | 2011 | Scotia 2011 | DB1 | 58 | -60.11104 | -34.82669 | 439 |
| Ophionotus | victoriae | SIOBICS4314 | SIOBICE5179 | Discovery Bank | 32 |  | 2011 | Scotia 2011 | DB1 | 58 | -60.11104 | -34.82669 | 439 |
| Ophionotus | victoriae | SIOBICS4315 | SIOBICE5258 | Discovery Bank | 32 |  | 2011 | Scotia 2011 | DB1 | 58 | -60.11104 | -34.82669 | 439 |
| Ophionotus | victoriae | SIOBICS4316 | SIOBICE5261 | Discovery Bank | 32 |  | 2011 | Scotia 2011 | DB1 | 58 | -60.11104 | -34.82669 | 439 |
| Ophionotus | victoriae | SIOBICS4318 | SIOBICE5222 | Discovery Bank | 32 |  | 2011 | Scotia 2011 | DB1 | 58 | -60.11104 | -34.82669 | 439 |
| Ophionotus | victoriae | SIOBICS4319 | SIOBICE5259 | Discovery Bank | 32 |  | 2011 | Scotia 2011 | DB1 | 58 | -60.11104 | -34.82669 | 439 |
| Ophionotus | victoriae | SIOBICS4320 | SIOBICE5231 | Discovery Bank | 32 |  | 2011 | Scotia 2011 | DB1 | 58 | -60.11104 | -34.82669 | 439 |
| Ophionotus | victoriae | SIOBICS4323 | SIOBICE5240 | Discovery Bank | 32 |  | 2011 | Scotia 2011 | DB1 | 58 | -60.11104 | -34.82669 | 439 |
| Ophionotus | victoriae | SIOBICS4325 | SIOBICE5264 | Discovery Bank | 32 |  | 2011 | Scotia 2011 | DB1 | 58 | -60.11104 | -34.82669 | 439 |
| Ophionotus | victoriae | SIOBICS4321 | SIOBICE5260 | Discovery Bank | 33 |  | 2011 | Scotia 2011 | DB1 | 58 | -60.11104 | -34.82669 | 439 |
| Ophionotus | victoriae | SIOBICS4324 | SIOBICE5263 | Discovery Bank | 35 |  | 2011 | Scotia 2011 | DB1 | 58 | -60.11104 | -34.82669 | 439 |
| Ophionotus | victoriae | SIOBICS4308 | SIOBICE5194 | Discovery Bank | 130 |  | 2011 | Scotia 2011 | DB1 | 58 | -60.11104 | -34.82669 | 439 |
| Ophionotus | victoriae | SIOBICS4312 | SIOBICE5218 | Discovery Bank | 131 |  | 2011 | Scotia 2011 | DB1 | 58 | -60.11104 | -34.82669 | 439 |
| Ophionotus | victoriae | SIOBICS4313 | SIOBICE5224 | Discovery Bank | 132 |  | 2011 | Scotia 2011 | DB1 | 58 | -60.11104 | -34.82669 | 439 |
| Ophionotus | victoriae | SIOBICS4317 | SIOBICE5188 | Discovery Bank | 133 |  | 2011 | Scotia 2011 | DB1 | 58 | -60.11104 | -34.82669 | 439 |
| Ophionotus | victoriae | SIOBICS4322 | SIOBICE5262 | Discovery Bank | 134 |  | 2011 | Scotia 2011 | DB1 | 58 | -60.11104 | -34.82669 | 439 |
| Ophionotus | victoriae | SIOBICS1312K | SIOBICE4771 | Elephant Island | 4 |  | 2011 | Scotia 2011 | EI1 | 81 | -61.21769 | -54.25476 | 202 |
| Ophionotus | victoriae | SIOBICS1312A | SIOBICE4771 | Elephant Island | 5 |  | 2011 | Scotia 2011 | EI1 | 81 | -61.21769 | -54.25476 | 202 |
| Ophionotus | victoriae | SIOBICS1312B | SIOBICE4771 | Elephant Island | 5 |  | 2011 | Scotia 2011 | EI1 | 81 | -61.21769 | -54.25476 | 202 |
| Ophionotus | victoriae | SIOBICS1312F | SIOBICE4771 | Elephant Island | 5 |  | 2011 | Scotia 2011 | EI1 | 81 | -61.21769 | -54.25476 | 202 |
| Ophionotus | victoriae | SIOBICS1312H | SIOBICE4771 | Elephant Island | 5 |  | 2011 | Scotia 2011 | EI1 | 81 | -61.21769 | -54.25476 | 202 |
| Ophionotus | victoriae | SIOBICS6775A | SIOBICE4803 | Elephant Island | 5 |  | 2011 | Scotia 2011 | EI2 | 84 | -61.30365 | -55.70822 | 170 |
| Ophionotus | victoriae | SIOBICS1312I | SIOBICE4771 | Elephant Island | 6 |  | 2011 | Scotia 2011 | EI1 | 81 | -61.21769 | -54.25476 | 202 |
| Ophionotus | victoriae | SIOBICS6741 | SIOBICE5209 | Elephant Island | 6 |  | 2011 | Scotia 2011 | EI2 | 83 | -61.33877 | -55.62494 | 143 |
| Ophionotus | victoriae | SIOBICS6779 | SIOBICE5289 | Elephant Island | 6 |  | 2011 | Scotia 2011 | EI2 | 83 | -61.33877 | -55.62494 | 143 |
| Ophionotus | victoriae | SIOBICS1312C | SIOBICE4771 | Elephant Island | 7 |  | 2011 | Scotia 2011 | EI1 | 81 | -61.21769 | -54.25476 | 202 |
| Ophionotus | victoriae | SIOBICS1312J | SIOBICE4771 | Elephant Island | 7 |  | 2011 | Scotia 2011 | EI1 | 81 | -61.21769 | -54.25476 | 202 |
| Ophionotus | victoriae | SIOBICS1312D | SIOBICE4771 | Elephant Island | 8 |  | 2011 | Scotia 2011 | EI1 | 81 | -61.21769 | -54.25476 | 202 |
| Ophionotus | victoriae | SIOBICS1312E | SIOBICE4771 | Elephant Island | 8 |  | 2011 | Scotia 2011 | EI1 | 81 | -61.21769 | -54.25476 | 202 |
| Ophionotus | victoriae | SIOBICS6743 | SIOBICE5237 | Elephant Island | 24 |  | 2011 | Scotia 2011 | EI2 | 83 | -61.33877 | -55.62494 | 143 |
| Ophionotus | victoriae | SIOBICS1312L | SIOBICE4771 | Elephant Island | 25 |  | 2011 | Scotia 2011 | EI1 | 81 | -61.21769 | -54.25476 | 202 |
| Ophionotus | victoriae | AP43 (haplotype ID) |  | Elephant Island | 44 | FJ917349 | 2008 | JR179 | BIO6-AGT-2A |  | -61.2 | -54.733333 | 239 |
| Ophionotus | victoriae | SIOBICS1312G | SIOBICE4771 | Elephant Island | 84 |  | 2011 | Scotia 2011 | EI1 | 81 | -61.21769 | -54.25476 | 202 |
| Ophionotus | victoriae | PNG635 | PNG635 | Heard Island | 111 |  |  | AAD | SC50 | H257 | -52.385 | 75.0516666 | 240 |
| Ophionotus | victoriae | SIOBICS4494 | SIOBICE5353 | Herdman Bank | 5 |  | 2011 | Scotia 2011 | HB1 | 51 | -59.89862 | -32.4511 | 520 |
| Ophionotus | victoriae | SIOBICS4491 | SIOBICE5210 | Herdman Bank | 11 |  | 2011 | Scotia 2011 | HB1 | 51 | -59.89862 | -32.4511 | 520 |
| Ophionotus | victoriae | SIOBICS4472K | SIOBICE4791 | Herdman Bank | 31 |  | 2011 | Scotia 2011 | HB1 | 50 | -59.86338 | -32.47014 | 600 |
| Ophionotus | victoriae | SIOBICS4472L | SIOBICE4791 | Herdman Bank | 31 |  | 2011 | Scotia 2011 | HB1 | 50 | -59.86338 | -32.47014 | 600 |
| Ophionotus | victoriae | SIOBICS4472N | SIOBICE4791 | Herdman Bank | 31 |  | 2011 | Scotia 2011 | HB1 | 50 | -59.86338 | -32.47014 | 600 |
| Ophionotus | victoriae | SIOBICS4472Q | SIOBICE4791 | Herdman Bank | 31 |  | 2011 | Scotia 2011 | HB1 | 50 | -59.86338 | -32.47014 | 600 |
| Ophionotus | victoriae | SIOBICS4472R | SIOBICE4791 | Herdman Bank | 31 |  | 2011 | Scotia 2011 | HB1 | 50 | -59.86338 | -32.47014 | 600 |
| Ophionotus | victoriae | SIOBICS4472T | SIOBICE4791 | Herdman Bank | 31 |  | 2011 | Scotia 2011 | HB1 | 50 | -59.86338 | -32.47014 | 600 |
| Ophionotus | victoriae | SIOBICS4493 | SIOBICE5352 | Herdman Bank | 32 |  | 2011 | Scotia 2011 | HB1 | 51 | -59.89862 | -32.4511 | 520 |
| Ophionotus | victoriae | SIOBICS4495 | SIOBICE5354 | Herdman Bank | 32 |  | 2011 | Scotia 2011 | HB1 | 51 | -59.89862 | -32.4511 | 520 |
| Ophionotus | victoriae | SIOBICS4496 | SIOBICE5355 | Herdman Bank | 32 |  | 2011 | Scotia 2011 | HB1 | 51 | -59.89862 | -32.4511 | 520 |
| Ophionotus | victoriae | SIOBICS4497 | SIOBICE5356 | Herdman Bank | 32 |  | 2011 | Scotia 2011 | HB1 | 51 | -59.89862 | -32.4511 | 520 |
| Ophionotus | victoriae | SIOBICS4498 | SIOBICE5357 | Herdman Bank | 32 |  | 2011 | Scotia 2011 | HB1 | 51 | -59.89862 | -32.4511 | 520 |
| Ophionotus | victoriae | SIOBICS4472M | SIOBICE4791 | Herdman Bank | 34 |  | 2011 | Scotia 2011 | HB1 | 50 | -59.86338 | -32.47014 | 600 |
| Ophionotus | victoriae | SIOBICS4472O | SIOBICE4791 | Herdman Bank | 35 |  | 2011 | Scotia 2011 | HB1 | 50 | -59.86338 | -32.47014 | 600 |
| Ophionotus | victoriae | SIOBICS4472P | SIOBICE4791 | Herdman Bank | 35 |  | 2011 | Scotia 2011 | HB1 | 50 | -59.86338 | -32.47014 | 600 |
| Ophionotus | victoriae | SIOBICS4472S | SIOBICE4791 | Herdman Bank | 35 |  | 2011 | Scotia 2011 | HB1 | 50 | -59.86338 | -32.47014 | 600 |
| Ophionotus | victoriae | SIOBICS4499 | SIOBICE5358 | Herdman Bank | 35 |  | 2011 | Scotia 2011 | HB1 | 51 | -59.89862 | -32.4511 | 520 |
| Ophionotus | victoriae | SIOBICS4500 | SIOBICE5360 | Herdman Bank | 121 |  | 2011 | Scotia 2011 | HB1 | 51 | -59.89862 | -32.4511 | 520 |
| Ophionotus | victoriae | SIOBICS4492 | SIOBICE5351 | Herdman Bank | 135 |  | 2011 | Scotia 2011 | HB1 | 51 | -59.89862 | -32.4511 | 520 |
| Ophionotus | victoriae | DSOPH2904 |  | Larsen Ice Shelf | 1 | FJ917337 | 2011 | Polarstern ANT-XXVII/3 | PS77_250-6 |  | -65.384 | -61.548 | 567 |
| Ophionotus | victoriae | DSOPH3807 |  | Larsen Ice Shelf | 1 | FJ917337 | 2011 | Polarstern ANT-XXVII/3 | PS77_239-3 |  | -66.20017 | -60.171 | 360 |
| Ophionotus | victoriae | DSOPH3810 |  | Larsen Ice Shelf | 1 | FJ917337 | 2011 | Polarstern ANT-XXVII/3 | PS77_239-3 |  | -66.20017 | -60.171 | 360 |
| Ophionotus | victoriae | DSOPH3859 |  | Larsen Ice Shelf | 1 | FJ917337 | 2011 | Polarstern ANT-XXVII/3 | PS77_233-3 |  | -65.558 | -61.622 | 324 |
| Ophionotus | victoriae | DSOPH3871 |  | Larsen Ice Shelf | 1 | FJ917337 | 2011 | Polarstern ANT-XXVII/3 | PS77_233-3 |  | -65.558 | -61.622 | 324 |
| Ophionotus | victoriae | WAMZ88551 | WAMZ88551 | Larsen Ice Shelf | 1 |  | 2011 | Polarstern ANT-XXVII/3 | PS77_228-3 |  | -64.903056 | -60.490278 | 570 |
| Ophionotus | victoriae | WAMZ88553 | WAMZ88553 | Larsen Ice Shelf | 1 |  | 2011 | Polarstern ANT-XXVII/3 | PS77_228-4 |  | -64.932778 | -60.560278 | 329 |
| Ophionotus | victoriae | WAMZ88560 | WAMZ88560 | Larsen Ice Shelf | 1 |  | 2011 | Polarstern ANT-XXVII/3 | PS77_233-3 |  | -65.558333 | -61.623056 | 320 |
| Ophionotus | victoriae | WAMZ88574 | WAMZ88574 | Larsen Ice Shelf | 1 |  | 2011 | Polarstern ANT-XXVII/3 | PS77_233-3 |  | -65.558333 | -61.623056 | 320 |
| Ophionotus | victoriae | 57.3C.13 |  | Larsen Ice Shelf | 4 | FJ917313 | 2004 | LMG-04-14 | 40 |  | -63.666667 | -57.329167 | 335 |
| Ophionotus | victoriae | Op913_3E_7 |  | Larsen Ice Shelf | 4 | FJ917313 | 2013 | LMG-13-12 | 8 |  | -64.134392 | -56.860217 | 310 |
| Ophionotus | victoriae | Op917_3E_1 |  | Larsen Ice Shelf | 4 | KY048266 | 2013 | LMG-13-12 | 10 |  | -63.685783 | -56.859 | 400 |
| Ophionotus | victoriae | Op917_3E_5 |  | Larsen Ice Shelf | 4 | FJ917313 | 2013 | LMG-13-12 | 10 |  | -63.685783 | -56.859 | 400 |
| Ophionotus | victoriae | Op917_3E_8 |  | Larsen Ice Shelf | 4 | FJ917313 | 2013 | LMG-13-12 | 10 |  | -63.685783 | -56.859 | 400 |
| Ophionotus | victoriae | Op917_3E_9 |  | Larsen Ice Shelf | 4 | FJ917313 | 2013 | LMG-13-12 | 10 |  | -63.685783 | -56.859 | 400 |
| Ophionotus | victoriae | DSOPH2924 |  | Larsen Ice Shelf | 5 | FJ917320 | 2011 | Polarstern ANT-XXVII/3 | PS77_231-3 |  | -64.914 | -60.515 | 314 |
| Ophionotus | victoriae | DSOPH3837 |  | Larsen Ice Shelf | 5 | FJ917320 | 2011 | Polarstern ANT-XXVII/3 | PS77_226-7 |  | -64.914 | -60.621 | 226 |
| Ophionotus | victoriae | DSOPH3839 |  | Larsen Ice Shelf | 5 | FJ917320 | 2011 | Polarstern ANT-XXVII/3 | PS77_226-7 |  | -64.914 | -60.621 | 226 |
| Ophionotus | victoriae | WAMZ88561 | WAMZ88561 | Larsen Ice Shelf | 5 |  | 2011 | Polarstern ANT-XXVII/3 | PS77_226-7 |  | -64.913333 | -60.624333 | 226 |
| Ophionotus | victoriae | WAMZ88566 | WAMZ88566 | Larsen Ice Shelf | 5 |  | 2011 | Polarstern ANT-XXVII/3 | PS77_226-7 |  | -64.913333 | -60.624333 | 226 |
| Ophionotus | victoriae | 57.3C.03 |  | Larsen Ice Shelf | 6 | FJ917309 | 2004 | LMG-04-14 | 40 |  | -63.666667 | -57.329167 | 335 |
| Ophionotus | victoriae | 57.3C.14 |  | Larsen Ice Shelf | 6 | FJ917309 | 2004 | LMG-04-14 | 40 |  | -63.666667 | -57.329167 | 335 |
| Ophionotus | victoriae | DSOPH3838 |  | Larsen Ice Shelf | 6 | KY048254 | 2011 | Polarstern ANT-XXVII/3 | PS77_226-7 |  | -64.914001 | -60.620998 | 226 |
| Ophionotus | victoriae | Op895_3E_5 |  | Larsen Ice Shelf | 6 | FJ917309 | 2013 | LMG-13-12 | 6 |  | -64.302283 | -56.136417 | 290 |
| Ophionotus | victoriae | Op895_3E_7 |  | Larsen Ice Shelf | 6 | FJ917309 | 2013 | LMG-13-12 | 6 |  | -64.302283 | -56.136417 | 290 |
| Ophionotus | victoriae | WAMZ88567 | WAMZ88567 | Larsen Ice Shelf | 6 |  | 2011 | Polarstern ANT-XXVII/3 | PS77_226-7 |  | -64.913333 | -60.624333 | 226 |
| Ophionotus | victoriae | DSOPH2918 |  | Larsen Ice Shelf | 7 | FJ917322 | 2011 | Polarstern ANT-XXVII/3 | PS77_237-2 |  | -66.209 | -60.162 | 383 |
| Ophionotus | victoriae | DSOPH3809 |  | Larsen Ice Shelf | 7 | FJ917322 | 2011 | Polarstern ANT-XXVII/3 | PS77_239-3 |  | -66.20017 | -60.171 | 360 |
| Ophionotus | victoriae | DSOPH3876 |  | Larsen Ice Shelf | 7 | FJ917322 | 2011 | Polarstern ANT-XXVII/3 | PS77_233-3 |  | -65.558 | -61.622 | 324 |
| Ophionotus | victoriae | Op895_3E_10 |  | Larsen Ice Shelf | 7 | KY048263 | 2013 | LMG-13-12 | 6 |  | -64.302283 | -56.136417 | 290 |
| Ophionotus | victoriae | WAMZ88556 | WAMZ88556 | Larsen Ice Shelf | 7 |  | 2011 | Polarstern ANT-XXVII/3 | PS77_237-2 |  | -66.208 | -60.161333 | 362 |
| Ophionotus | victoriae | DSOPH3848 |  | Larsen Ice Shelf | 8 | FJ917316 | 2011 | Polarstern ANT-XXVII/3 | PS77_228-3 |  | -64.918 | -60.537 | 280 |
| Ophionotus | victoriae | DSOPH3892 |  | Larsen Ice Shelf | 8 | FJ917316 | 2011 | Polarstern ANT-XXVII/3 | PS77_228-4 |  | -64.929 | -60.565 | 316 |
| Ophionotus | victoriae | WAMZ88557 | WAMZ88557 | Larsen Ice Shelf | 8 |  | 2011 | Polarstern ANT-XXVII/3 | PS77_228-4 |  | -64.932778 | -60.560278 | 329 |
| Ophionotus | victoriae | 57.3C.11 |  | Larsen Ice Shelf | 10 | FJ917312 | 2004 | LMG-04-14 | 40 |  | -63.666667 | -57.329167 | 335 |
| Ophionotus | victoriae | DSOPH2923 |  | Larsen Ice Shelf | 10 | KY048244 | 2011 | Polarstern ANT-XXVII/3 | PS77_231-3 |  | -64.914 | -60.515 | 314 |
| Ophionotus | victoriae | Op914_3E_10 |  | Larsen Ice Shelf | 10 | FJ917312 | 2013 | LMG-13-12 | 9 |  | -63.742367 | -57.431867 | 692 |
| Ophionotus | victoriae | 321.2C.04 |  | Larsen Ice Shelf | 14 | FJ917310 | 2006 | LMG-06-05 | 21 |  | -64.350361 | -57.076617 | 146 |
| Ophionotus | victoriae | 57.3C.05 |  | Larsen Ice Shelf | 14 | FJ917310 | 2004 | LMG-04-14 | 40 |  | -63.666667 | -57.329167 | 335 |
| Ophionotus | victoriae | 57.3C.10 |  | Larsen Ice Shelf | 14 | FJ917310 | 2004 | LMG-04-14 | 40 |  | -63.666667 | -57.329167 | 335 |
| Ophionotus | victoriae | 57.3C.12 |  | Larsen Ice Shelf | 14 | FJ917310 | 2004 | LMG-04-14 | 40 |  | -63.666667 | -57.329167 | 335 |
| Ophionotus | victoriae | 59.2C.03 |  | Larsen Ice Shelf | 14 | FJ917310 | 2004 | LMG-04-14 | 40 |  | -63.666667 | -57.329167 | 335 |
| Ophionotus | victoriae | 59.2C.06 |  | Larsen Ice Shelf | 14 | FJ917310 | 2004 | LMG-04-14 | 40 |  | -63.666667 | -57.329167 | 335 |
| Ophionotus | victoriae | 59.2C.07 |  | Larsen Ice Shelf | 14 | FJ917310 | 2004 | LMG-04-14 | 40 |  | -63.666667 | -57.329167 | 335 |
| Ophionotus | victoriae | DSOPH2888 |  | Larsen Ice Shelf | 14 | FJ917310 | 2011 | Polarstern ANT-XXVII/3 | PS77_248-3 |  | -65.924 | -60.332 | 443 |
| Ophionotus | victoriae | DSOPH3835 |  | Larsen Ice Shelf | 14 | FJ917310 | 2011 | Polarstern ANT-XXVII/3 | PS77_226-7 |  | -64.914 | -60.621 | 226 |
| Ophionotus | victoriae | Op913_3E_1 |  | Larsen Ice Shelf | 14 | FJ917310 | 2013 | LMG-13-12 | 8 |  | -64.134392 | -56.860217 | 310 |
| Ophionotus | victoriae | Op913_3E_2 |  | Larsen Ice Shelf | 14 | FJ917310 | 2013 | LMG-13-12 | 8 |  | -64.134392 | -56.860217 | 310 |
| Ophionotus | victoriae | Op913_3E_4 |  | Larsen Ice Shelf | 14 | FJ917310 | 2013 | LMG-13-12 | 8 |  | -64.134392 | -56.860217 | 310 |
| Ophionotus | victoriae | Op913_3E_5 |  | Larsen Ice Shelf | 14 | FJ917310 | 2013 | LMG-13-12 | 8 |  | -64.134392 | -56.860217 | 310 |
| Ophionotus | victoriae | Op913_3E_6 |  | Larsen Ice Shelf | 14 | FJ917310 | 2013 | LMG-13-12 | 8 |  | -64.134392 | -56.860217 | 310 |
| Ophionotus | victoriae | Op913_3E_9 |  | Larsen Ice Shelf | 14 | FJ917310 | 2013 | LMG-13-12 | 8 |  | -64.134392 | -56.860217 | 310 |
| Ophionotus | victoriae | Op914_3E_8 |  | Larsen Ice Shelf | 14 | FJ917310 | 2013 | LMG-13-12 | 9 |  | -63.742367 | -57.431867 | 692 |
| Ophionotus | victoriae | Op914_3E_9 |  | Larsen Ice Shelf | 14 | FJ917310 | 2013 | LMG-13-12 | 9 |  | -63.742367 | -57.431867 | 692 |
| Ophionotus | victoriae | Op917_3E_10 |  | Larsen Ice Shelf | 14 | FJ917310 | 2013 | LMG-13-12 | 10 |  | -63.685783 | -56.859 | 400 |
| Ophionotus | victoriae | Op917_3E_4 |  | Larsen Ice Shelf | 14 | FJ917310 | 2013 | LMG-13-12 | 10 |  | -63.685783 | -56.859 | 400 |
| Ophionotus | victoriae | Op917_3E_6 |  | Larsen Ice Shelf | 14 | FJ917310 | 2013 | LMG-13-12 | 10 |  | -63.685783 | -56.859 | 400 |
| Ophionotus | victoriae | Op917_3E_7 |  | Larsen Ice Shelf | 14 | FJ917310 | 2013 | LMG-13-12 | 10 |  | -63.685783 | -56.859 | 400 |
| Ophionotus | victoriae | WAMZ88564 | WAMZ88564 | Larsen Ice Shelf | 14 |  | 2011 | Polarstern ANT-XXVII/3 | PS77_233-3 |  | -65.558333 | -61.623056 | 320 |
| Ophionotus | victoriae | WAMZ88565 | WAMZ88565 | Larsen Ice Shelf | 14 |  | 2011 | Polarstern ANT-XXVII/3 | PS77_233-3 |  | -65.558333 | -61.623056 | 320 |
| Ophionotus | victoriae | WAMZ88572 | WAMZ88572 | Larsen Ice Shelf | 14 |  | 2011 | Polarstern ANT-XXVII/3 | PS77_248-3 |  | -65.928167 | -60.3345 | 433 |
| Ophionotus | victoriae | WAMZ88581 | WAMZ88581 | Larsen Ice Shelf | 14 |  | 2011 | Polarstern ANT-XXVII/3 | PS77_226-7 |  | -64.913333 | -60.624333 | 226 |
| Ophionotus | victoriae | 57.3C.08 |  | Larsen Ice Shelf | 20 | FJ917311 | 2004 | LMG-04-14 | 40 |  | -63.666667 | -57.329167 | 335 |
| Ophionotus | victoriae | Op917_3E_2 |  | Larsen Ice Shelf | 20 | FJ917311 | 2013 | LMG-13-12 | 10 |  | -63.685783 | -56.859 | 400 |
| Ophionotus | victoriae | 321.2C.01 |  | Larsen Ice Shelf | 24 | FJ917318 | 2006 | LMG-06-05 | 21 |  | -64.350361 | -57.076617 | 146 |
| Ophionotus | victoriae | DSOPH3808 |  | Larsen Ice Shelf | 24 | FJ917318 | 2011 | Polarstern ANT-XXVII/3 | PS77_239-3 |  | -66.20017 | -60.171 | 360 |
| Ophionotus | victoriae | DSOPH3847 |  | Larsen Ice Shelf | 24 | FJ917318 | 2011 | Polarstern ANT-XXVII/3 | PS77_228-3 |  | -64.918 | -60.537 | 280 |
| Ophionotus | victoriae | DSOPH3850 |  | Larsen Ice Shelf | 24 | FJ917318 | 2011 | Polarstern ANT-XXVII/3 | PS77_228-3 |  | -64.918 | -60.537 | 280 |
| Ophionotus | victoriae | DSOPH3851 |  | Larsen Ice Shelf | 24 | FJ917318 | 2011 | Polarstern ANT-XXVII/3 | PS77_228-3 |  | -64.918 | -60.537 | 280 |
| Ophionotus | victoriae | Op914_3E_6 |  | Larsen Ice Shelf | 24 | FJ917318 | 2013 | LMG-13-12 | 9 |  | -63.742367 | -57.431867 | 692 |
| Ophionotus | victoriae | Op914_3E_7 |  | Larsen Ice Shelf | 24 | FJ917318 | 2013 | LMG-13-12 | 9 |  | -63.742367 | -57.431867 | 692 |
| Ophionotus | victoriae | WAMZ88568 | WAMZ88568 | Larsen Ice Shelf | 24 |  | 2011 | Polarstern ANT-XXVII/3 | PS77_252-7 |  | -64.7035 | -60.5295 | 343 |
| Ophionotus | victoriae | 321.2C.03 |  | Larsen Ice Shelf | 25 | FJ917319 | 2006 | LMG-06-05 | 21 |  | -64.350361 | -57.076617 | 146 |
| Ophionotus | victoriae | 321.2C.06 |  | Larsen Ice Shelf | 25 | FJ917319 | 2006 | LMG-06-05 | 21 |  | -64.350361 | -57.076617 | 146 |
| Ophionotus | victoriae | Op895_3E_2 |  | Larsen Ice Shelf | 25 | FJ917319 | 2013 | LMG-13-12 | 6 |  | -64.302283 | -56.136417 | 290 |
| Ophionotus | victoriae | Op895_3E_4 |  | Larsen Ice Shelf | 25 | FJ917319 | 2013 | LMG-13-12 | 6 |  | -64.302283 | -56.136417 | 290 |
| Ophionotus | victoriae | Op895_3E_8 |  | Larsen Ice Shelf | 25 | FJ917319 | 2013 | LMG-13-12 | 6 |  | -64.302283 | -56.136417 | 290 |
| Ophionotus | victoriae | Op913_3E_8 |  | Larsen Ice Shelf | 25 | FJ917319 | 2013 | LMG-13-12 | 8 |  | -64.134392 | -56.860217 | 310 |
| Ophionotus | victoriae | Op914_3E_2 |  | Larsen Ice Shelf | 25 | FJ917319 | 2013 | LMG-13-12 | 8 |  | -64.134392 | -56.860217 | 310 |
| Ophionotus | victoriae | Op917_3E_3 |  | Larsen Ice Shelf | 25 | KY048267 | 2013 | LMG-13-12 | 10 |  | -63.685783 | -56.859 | 400 |
| Ophionotus | victoriae | Op895_3E_3 |  | Larsen Ice Shelf | 32 | KY048264 | 2013 | LMG-13-12 | 6 |  | -64.302283 | -56.136417 | 290 |
| Ophionotus | victoriae | 321.2C.02 |  | Larsen Ice Shelf | 38 | FJ917341 | 2006 | LMG-06-05 | 21 |  | -64.350361 | -57.076617 | 146 |
| Ophionotus | victoriae | Op913_3E_3 |  | Larsen Ice Shelf | 55 | KY048218 | 2013 | LMG-13-12 | 8 |  | -64.134392 | -56.860217 | 310 |
| Ophionotus | victoriae | Op913_3E_10 |  | Larsen Ice Shelf | 58 | KY048231 | 2013 | LMG-13-12 | 8 |  | -64.134392 | -56.860217 | 310 |
| Ophionotus | victoriae | DSOPH2903 |  | Larsen Ice Shelf | 64 | KY048228 | 2011 | Polarstern ANT-XXVII/3 | PS77_250-6 |  | -65.384 | -61.548 | 567 |
| Ophionotus | victoriae | WAMZ88582 | WAMZ88582 | Larsen Ice Shelf | 64 |  | 2011 | Polarstern ANT-XXVII/3 | PS77_250-6 |  | -65.380833 | -61.556667 | 581 |
| Ophionotus | victoriae | DSOPH2867 |  | Larsen Ice Shelf | 74 | KY048241 | 2011 | Polarstern ANT-XXVII/3 | PS77_252-3 |  | -64.694 | -60.518 | 316 |
| Ophionotus | victoriae | WAMZ88555 | WAMZ88555 | Larsen Ice Shelf | 74 |  | 2011 | Polarstern ANT-XXVII/3 | PS77_255-3 |  | -64.832778 | -60.596667 | 682 |
| Ophionotus | victoriae | DSOPH2902 |  | Larsen Ice Shelf | 75 | KY048242 | 2011 | Polarstern ANT-XXVII/3 | PS77_250-6 |  | -65.384 | -61.548 | 567 |
| Ophionotus | victoriae | DSOPH2905 |  | Larsen Ice Shelf | 75 | KY048242 | 2011 | Polarstern ANT-XXVII/3 | PS77_250-6 |  | -65.384 | -61.548 | 567 |
| Ophionotus | victoriae | DSOPH2906 |  | Larsen Ice Shelf | 75 | KY048242 | 2011 | Polarstern ANT-XXVII/3 | PS77_250-6 |  | -65.384 | -61.548 | 567 |
| Ophionotus | victoriae | WAMZ88578 | WAMZ88578 | Larsen Ice Shelf | 75 |  | 2011 | Polarstern ANT-XXVII/3 | PS77_250-6 |  | -65.380833 | -61.556667 | 581 |
| Ophionotus | victoriae | DSOPH2914 |  | Larsen Ice Shelf | 76 | KY048243 | 2011 | Polarstern ANT-XXVII/3 | PS77_235-8 |  | -65.528 | -61.552 | 449 |
| Ophionotus | victoriae | DSOPH3872 |  | Larsen Ice Shelf | 76 | KY048243 | 2011 | Polarstern ANT-XXVII/3 | PS77_233-3 |  | -65.558 | -61.622 | 324 |
| Ophionotus | victoriae | WAMZ88569 | WAMZ88569 | Larsen Ice Shelf | 76 |  | 2011 | Polarstern ANT-XXVII/3 | PS77_233-3 |  | -65.558333 | -61.623056 | 320 |
| Ophionotus | victoriae | DSOPH3873 |  | Larsen Ice Shelf | 83 | KY048252 | 2011 | Polarstern ANT-XXVII/3 | PS77_233-3 |  | -65.557999 | -61.621998 | 324 |
| Ophionotus | victoriae | WAMZ88573 | WAMZ88573 | Larsen Ice Shelf | 83 |  | 2011 | Polarstern ANT-XXVII/3 | PS77_248-3 |  | -65.928167 | -60.3345 | 433 |
| Ophionotus | victoriae | WAMZ88575 | WAMZ88575 | Larsen Ice Shelf | 83 |  | 2011 | Polarstern ANT-XXVII/3 | PS77_233-3 |  | -65.558333 | -61.623056 | 320 |
| Ophionotus | victoriae | DSOPH3836 |  | Larsen Ice Shelf | 84 | KY048253 | 2011 | Polarstern ANT-XXVII/3 | PS77_226-7 |  | -64.914001 | -60.620998 | 226 |
| Ophionotus | victoriae | DSOPH3849 |  | Larsen Ice Shelf | 85 | KY048255 | 2011 | Polarstern ANT-XXVII/3 | PS77_228-3 |  | -64.918001 | -60.537 | 280 |
| Ophionotus | victoriae | WAMZ88563 | WAMZ88563 | Larsen Ice Shelf | 85 |  | 2011 | Polarstern ANT-XXVII/3 | PS77_228-3 |  | -64.903056 | -60.490278 | 570 |
| Ophionotus | victoriae | Op914_3E_3 |  | Larsen Ice Shelf | 92 | KY048265 | 2013 | LMG-13-12 | 9 |  | -63.742367 | -57.431867 | 692 |
| Ophionotus | victoriae | Op914_3E_5 |  | Larsen Ice Shelf | 92 | KY048265 | 2013 | LMG-13-12 | 9 |  | -63.742367 | -57.431867 | 692 |
| Ophionotus | victoriae | DSOPH3811 |  | Larsen Ice Shelf | 93 | KY048268 | 2011 | Polarstern ANT-XXVII/3 | PS77_239-3 |  | -66.20017 | -60.171 | 360 |
| Ophionotus | victoriae | WAMZ88552 | WAMZ88552 | Larsen Ice Shelf | 158 |  | 2011 | Polarstern ANT-XXVII/3 | PS77_250-6 |  | -65.380833 | -61.556667 | 581 |
| Ophionotus | victoriae | WAMZ88562 | WAMZ88562 | Larsen Ice Shelf | 159 |  | 2011 | Polarstern ANT-XXVII/3 | PS77_228-4 |  | -64.932778 | -60.560278 | 329 |
| Ophionotus | victoriae | AAD107 | AAD107 | Prydz Bay | 1 |  | 2001 | AAD | AL27-130 | 58.4.2 | -66.7911667 | 62.442 | 213 |
| Ophionotus | victoriae | AAD140 | AAD140 | Prydz Bay | 1 |  | 2001 | AAD | AL27-127 | 58.4.2 | -66.7923333 | 62.096 | 270 |
| Ophionotus | victoriae | AAD141 | AAD141 | Prydz Bay | 1 |  | 2001 | AAD | AL27-127 | 58.4.2 | -66.7923333 | 62.096 | 270 |
| Ophionotus | victoriae | AAD143 | AAD143 | Prydz Bay | 1 |  | 2001 | AAD | AL27-127 | 58.4.2 | -66.7923333 | 62.096 | 270 |
| Ophionotus | victoriae | AAD139 | AAD139 | Prydz Bay | 2 |  | 2001 | AAD | AL27-127 | 58.4.2 | -66.7923333 | 62.096 | 270 |
| Ophionotus | victoriae | AAD145 | AAD145 | Prydz Bay | 3 |  | 2001 | AAD | AL27-127 | 58.4.2 | -66.7923333 | 62.096 | 270 |
| Ophionotus | victoriae | 36968A | NIWA36968 | Ross Sea | 1 |  | 2008 | TAN0802 |  | 100 | -76.202 | 176.248 | 447 |
| Ophionotus | victoriae | 36968B | NIWA36968 | Ross Sea | 1 |  | 2008 | TAN0802 |  | 100 | -76.202 | 176.248 | 447 |
| Ophionotus | victoriae | 36968C | NIWA36968 | Ross Sea | 1 |  | 2008 | TAN0802 |  | 100 | -76.202 | 176.248 | 447 |
| Ophionotus | victoriae | 36968E | NIWA36968 | Ross Sea | 1 |  | 2008 | TAN0802 |  | 100 | -76.202 | 176.248 | 447 |
| Ophionotus | victoriae | 36968F | NIWA36968 | Ross Sea | 1 |  | 2008 | TAN0802 |  | 100 | -76.202 | 176.248 | 447 |
| Ophionotus | victoriae | 37157A | NIWA37157 | Ross Sea | 1 |  | 2008 | TAN0802 |  | 117 | -72.59033333 | 175.3423333 | 175 |
| Ophionotus | victoriae | 37157B | NIWA37157 | Ross Sea | 1 |  | 2008 | TAN0802 |  | 117 | -72.59033333 | 175.3423333 | 175 |
| Ophionotus | victoriae | 94866B | NIWA94866 | Ross Sea | 1 |  | 2004 | TAN0402 |  | 132 | -71.64766693 | 170.1801605 | 172 |
| Ophionotus | victoriae | N0065 | NIWA85184 | Ross Sea | 1 |  | 2008 | TAN0802 |  | 61 | -75.621667 | 169.8045 | 521 |
| Ophionotus | victoriae | N0068 | NIWA85184 | Ross Sea | 1 |  | 2008 | TAN0802 |  | 61 | -75.621667 | 169.8045 | 521 |
| Ophionotus | victoriae | N0069 | NIWA85184 | Ross Sea | 1 |  | 2008 | TAN0802 |  | 61 | -75.621667 | 169.8045 | 521 |
| Ophionotus | victoriae | N0072 | NIWA85184 | Ross Sea | 1 |  | 2008 | TAN0802 |  | 61 | -75.621667 | 169.8045 | 521 |
| Ophionotus | victoriae | N0073 | NIWA85184 | Ross Sea | 1 |  | 2008 | TAN0802 |  | 61 | -75.621667 | 169.8045 | 521 |
| Ophionotus | victoriae | N0075 | NIWA85184 | Ross Sea | 1 |  | 2008 | TAN0802 |  | 61 | -75.621667 | 169.8045 | 521 |
| Ophionotus | victoriae | N0099 | NIWA85183 | Ross Sea | 1 |  | 2008 | TAN0802 |  | 77 | -76.833333 | 179.95 | 664 |
| Ophionotus | victoriae | N0100 | NIWA85183 | Ross Sea | 1 |  | 2008 | TAN0802 |  | 77 | -76.833333 | 179.95 | 664 |
| Ophionotus | victoriae | N0101 | NIWA84672 | Ross Sea | 1 |  | 2008 | TAN0802 |  | 77 | -76.833333 | 179.95 | 664 |
| Ophionotus | victoriae | N0105 | NIWA84672 | Ross Sea | 1 |  | 2008 | TAN0802 |  | 77 | -76.833333 | 179.95 | 664 |
| Ophionotus | victoriae | N0106 | NIWA84672 | Ross Sea | 1 |  | 2008 | TAN0802 |  | 77 | -76.833333 | 179.95 | 664 |
| Ophionotus | victoriae | N0107 | NIWA84672 | Ross Sea | 1 |  | 2008 | TAN0802 |  | 77 | -76.833333 | 179.95 | 664 |
| Ophionotus | victoriae | N0108 | NIWA84672 | Ross Sea | 1 |  | 2008 | TAN0802 |  | 77 | -76.833333 | 179.95 | 664 |
| Ophionotus | victoriae | Op762_2E |  | Ross Sea | 1 | FJ917337 | 2013 | NBP-12-10 | 21 |  | -78.06324 | -169.99115 | 549 |
| Ophionotus | victoriae | Op762_4C_1 |  | Ross Sea | 1 | KY048262 | 2013 | NBP-12-10 | 21 |  | -78.06324 | -169.99115 | 549 |
| Ophionotus | victoriae | Op762_5C_1 |  | Ross Sea | 1 | FJ917337 | 2013 | NBP-12-10 | 21 |  | -78.06324 | -169.99115 | 549 |
| Ophionotus | victoriae | Op762_6C_1 |  | Ross Sea | 1 | FJ917337 | 2013 | NBP-12-10 | 21 |  | -78.06324 | -169.99115 | 549 |
| Ophionotus | victoriae | Op762_6C_2 |  | Ross Sea | 1 | FJ917337 | 2013 | NBP-12-10 | 21 |  | -78.06324 | -169.99115 | 549 |
| Ophionotus | victoriae | Op787_5C_1 |  | Ross Sea | 1 | FJ917337 | 2013 | NBP-12-10 | 22 |  | -76.998275 | -175.0932 | 541 |
| Ophionotus | victoriae | Op787_5C_2 |  | Ross Sea | 1 | FJ917337 | 2013 | NBP-12-10 | 22 |  | -76.998275 | -175.0932 | 541 |
| Ophionotus | victoriae | Op787_5C_3 |  | Ross Sea | 1 | FJ917337 | 2013 | NBP-12-10 | 22 |  | -76.998275 | -175.0932 | 541 |
| Ophionotus | victoriae | Op787_5C_4 |  | Ross Sea | 1 | FJ917337 | 2013 | NBP-12-10 | 22 |  | -76.998275 | -175.0932 | 541 |
| Ophionotus | victoriae | Op787_6C_1 |  | Ross Sea | 1 | FJ917337 | 2013 | NBP-12-10 | 22 |  | -76.998275 | -175.0932 | 541 |
| Ophionotus | victoriae | Op787_6C_2 |  | Ross Sea | 1 | FJ917337 | 2013 | NBP-12-10 | 22 |  | -76.998275 | -175.0932 | 541 |
| Ophionotus | victoriae | Op787_6C_3 |  | Ross Sea | 1 | FJ917337 | 2013 | NBP-12-10 | 22 |  | -76.998275 | -175.0932 | 541 |
| Ophionotus | victoriae | Op787_6C_5 |  | Ross Sea | 1 | FJ917337 | 2013 | NBP-12-10 | 22 |  | -76.998275 | -175.0932 | 541 |
| Ophionotus | victoriae | Op803_3C_1 |  | Ross Sea | 1 | FJ917337 | 2013 | NBP-12-10 | 23 |  | -76.245261 | 174.50412 | 604 |
| Ophionotus | victoriae | Op803_3C_2 |  | Ross Sea | 1 | FJ917337 | 2013 | NBP-12-10 | 23 |  | -76.245261 | 174.50412 | 604 |
| Ophionotus | victoriae | Op803_3C_3 |  | Ross Sea | 1 | FJ917337 | 2013 | NBP-12-10 | 23 |  | -76.245261 | 174.50412 | 604 |
| Ophionotus | victoriae | Op803_3C_4 |  | Ross Sea | 1 | FJ917337 | 2013 | NBP-12-10 | 23 |  | -76.245261 | 174.50412 | 604 |
| Ophionotus | victoriae | Op803_3C_5 |  | Ross Sea | 1 | FJ917337 | 2013 | NBP-12-10 | 23 |  | -76.245261 | 174.50412 | 604 |
| Ophionotus | victoriae | Op803_3C_6 |  | Ross Sea | 1 | FJ917337 | 2013 | NBP-12-10 | 23 |  | -76.245261 | 174.50412 | 604 |
| Ophionotus | victoriae | Op803_4C_2 |  | Ross Sea | 1 | FJ917337 | 2013 | NBP-12-10 | 23 |  | -76.245261 | 174.50412 | 604 |
| Ophionotus | victoriae | Op803_4C_3 |  | Ross Sea | 1 | FJ917337 | 2013 | NBP-12-10 | 23 |  | -76.245261 | 174.50412 | 604 |
| Ophionotus | victoriae | Op806_2E |  | Ross Sea | 1 | FJ917337 | 2013 | NBP-12-10 | 24 |  | -76.9038 | 169.96525 | 764 |
| Ophionotus | victoriae | Op806_3C_3 |  | Ross Sea | 1 | FJ917337 | 2013 | NBP-12-10 | 24 |  | -76.9038 | 169.96525 | 764 |
| Ophionotus | victoriae | Op806_3C_4 |  | Ross Sea | 1 | FJ917337 | 2013 | NBP-12-10 | 24 |  | -76.9038 | 169.96525 | 764 |
| Ophionotus | victoriae | Op806_8C_2 |  | Ross Sea | 1 | FJ917337 | 2013 | NBP-12-10 | 24 |  | -76.9038 | 169.96525 | 764 |
| Ophionotus | victoriae | Op806_8C_3 |  | Ross Sea | 1 | FJ917337 | 2013 | NBP-12-10 | 24 |  | -76.9038 | 169.96525 | 764 |
| Ophionotus | victoriae | Op806_8C_4 |  | Ross Sea | 1 | FJ917337 | 2013 | NBP-12-10 | 24 |  | -76.9038 | 169.96525 | 764 |
| Ophionotus | victoriae | Op818_3C_1 |  | Ross Sea | 1 | FJ917337 | 2013 | NBP-12-10 | 25 |  | -75.833465 | 166.50549 | 552 |
| Ophionotus | victoriae | Op818_3C_2 |  | Ross Sea | 1 | FJ917337 | 2013 | NBP-12-10 | 25 |  | -75.833465 | 166.50549 | 552 |
| Ophionotus | victoriae | Op818_3C_4 |  | Ross Sea | 1 | FJ917337 | 2013 | NBP-12-10 | 25 |  | -75.833465 | 166.50549 | 552 |
| Ophionotus | victoriae | Op818_3C_5 |  | Ross Sea | 1 | FJ917337 | 2013 | NBP-12-10 | 25 |  | -75.833465 | 166.50549 | 552 |
| Ophionotus | victoriae | Op818_4C_1 |  | Ross Sea | 1 | FJ917337 | 2013 | NBP-12-10 | 25 |  | -75.833465 | 166.50549 | 552 |
| Ophionotus | victoriae | Op818_4C_2 |  | Ross Sea | 1 | FJ917337 | 2013 | NBP-12-10 | 25 |  | -75.833465 | 166.50549 | 552 |
| Ophionotus | victoriae | Op818_4C_5 |  | Ross Sea | 1 | FJ917337 | 2013 | NBP-12-10 | 25 |  | -75.833465 | 166.50549 | 552 |
| Ophionotus | victoriae | Op826_2E |  | Ross Sea | 1 | FJ917337 | 2013 | NBP-12-10 | 26 |  | -74.70781 | 168.40783 | 489 |
| Ophionotus | victoriae | Op826_3C_1 |  | Ross Sea | 1 | FJ917337 | 2013 | NBP-12-10 | 26 |  | -74.70781 | 168.40783 | 489 |
| Ophionotus | victoriae | Op843_2E |  | Ross Sea | 1 | FJ917337 | 2013 | NBP-12-10 | 28 |  | -74.995422 | 165.74422 | 1101 |
| Ophionotus | victoriae | Op843_7C_2 |  | Ross Sea | 1 | FJ917337 | 2013 | NBP-12-10 | 28 |  | -74.995422 | 165.74422 | 1101 |
| Ophionotus | victoriae | Op843_7C_3 |  | Ross Sea | 1 | FJ917337 | 2013 | NBP-12-10 | 28 |  | -74.995422 | 165.74422 | 1101 |
| Ophionotus | victoriae | PDIVA-A | SIOBICE4766 | Ross Sea | 1 |  | 2010 | SIO McMurdo |  | IVA | -77.5717 | 163.5117 |  |
| Ophionotus | victoriae | PDIVA-C | SIOBICE4766 | Ross Sea | 1 |  | 2010 | SIO McMurdo |  | IVA | -77.5717 | 163.5117 |  |
| Ophionotus | victoriae | PDIVA-D | SIOBICE4766 | Ross Sea | 1 |  | 2010 | SIO McMurdo |  | IVA | -77.5717 | 163.5117 |  |
| Ophionotus | victoriae | PDIVA-E | SIOBICE4766 | Ross Sea | 1 |  | 2010 | SIO McMurdo |  | IVA | -77.5717 | 163.5117 |  |
| Ophionotus | victoriae | PDIVA-F | SIOBICE4766 | Ross Sea | 1 |  | 2010 | SIO McMurdo |  | IVA | -77.5717 | 163.5117 |  |
| Ophionotus | victoriae | PDIVA-G | SIOBICE4766 | Ross Sea | 1 |  | 2010 | SIO McMurdo |  | IVA | -77.5717 | 163.5117 |  |
| Ophionotus | victoriae | PDIVA-I | SIOBICE4766 | Ross Sea | 1 |  | 2010 | SIO McMurdo |  | IVA | -77.5717 | 163.5117 |  |
| Ophionotus | victoriae | PDIVA-J | SIOBICE4766 | Ross Sea | 1 |  | 2010 | SIO McMurdo |  | IVA | -77.5717 | 163.5117 |  |
| Ophionotus | victoriae | PDIVA-K | SIOBICE4766 | Ross Sea | 1 |  | 2010 | SIO McMurdo |  | IVA | -77.5717 | 163.5117 |  |
| Ophionotus | victoriae | PDIVA-O | SIOBICE4766 | Ross Sea | 1 |  | 2010 | SIO McMurdo |  | IVA | -77.5717 | 163.5117 |  |
| Ophionotus | victoriae | PDIVA-P | SIOBICE4766 | Ross Sea | 1 |  | 2010 | SIO McMurdo |  | IVA | -77.5717 | 163.5117 |  |
| Ophionotus | victoriae | PDIVA-Q | SIOBICE4766 | Ross Sea | 1 |  | 2010 | SIO McMurdo |  | IVA | -77.5717 | 163.5117 |  |
| Ophionotus | victoriae | PDIVA-R | SIOBICE4766 | Ross Sea | 1 |  | 2010 | SIO McMurdo |  | IVA | -77.5717 | 163.5117 |  |
| Ophionotus | victoriae | PDIVA-S | SIOBICE4766 | Ross Sea | 1 |  | 2010 | SIO McMurdo |  | IVA | -77.5717 | 163.5117 |  |
| Ophionotus | victoriae | PDIVA-T | SIOBICE4766 | Ross Sea | 1 |  | 2010 | SIO McMurdo |  | IVA | -77.5717 | 163.5117 |  |
| Ophionotus | victoriae | 94869B | NIWA94869 | Ross Sea | 24 |  | 2004 | TAN0402 |  | 174 | -71.4936676 | 171.6041718 | 483 |
| Ophionotus | victoriae | A04N.01 |  | Ross Sea | 24 | GU227093 | 2008 |  |  |  | Cape Harlett |  |  |
| Ophionotus | victoriae | N0077 | NIWA85184 | Ross Sea | 24 |  | 2008 | TAN0802 |  | 61 | -75.621667 | 169.8045 | 521 |
| Ophionotus | victoriae | N0110 | NIWA84672 | Ross Sea | 24 |  | 2008 | TAN0802 |  | 77 | -76.833333 | 179.95 | 664 |
| Ophionotus | victoriae | N0102 | NIWA84672 | Ross Sea | 43 |  | 2008 | TAN0802 |  | 77 | -76.833333 | 179.95 | 664 |
| Ophionotus | victoriae | 140217A | NIWA140217 | Ross Sea | 49 |  | 2019 | TAN1901 | SRS2_7 | 175 | -75.5246667 | -172.992 | 1376 |
| Ophionotus | victoriae | 140217B | NIWA140217 | Ross Sea | 49 |  | 2019 | TAN1901 | SRS2_7 | 175 | -75.5246667 | -172.992 | 1376 |
| Ophionotus | victoriae | N0067 | NIWA85184 | Ross Sea | 49 |  | 2008 | TAN0802 |  | 61 | -75.621667 | 169.8045 | 521 |
| Ophionotus | victoriae | N0070 | NIWA85184 | Ross Sea | 49 |  | 2008 | TAN0802 |  | 61 | -75.621667 | 169.8045 | 521 |
| Ophionotus | victoriae | N0103 | NIWA84672 | Ross Sea | 49 |  | 2008 | TAN0802 |  | 77 | -76.833333 | 179.95 | 664 |
| Ophionotus | victoriae | Op803_4C_1 |  | Ross Sea | 49 | KY048234 | 2013 | NBP-12-10 | 23 |  | -76.245261 | 174.50412 | 604 |
| Ophionotus | victoriae | Op806_3C_2 |  | Ross Sea | 49 | KY048234 | 2013 | NBP-12-10 | 24 |  | -76.9038 | 169.96525 | 764 |
| Ophionotus | victoriae | PDIVA-L | SIOBICE4766 | Ross Sea | 50 |  | 2010 | SIO McMurdo |  | IVA | -77.5717 | 163.5117 |  |
| Ophionotus | victoriae | 36968D | NIWA36968 | Ross Sea | 91 |  | 2008 | TAN0802 |  | 100 | -76.202 | 176.248 | 447 |
| Ophionotus | victoriae | N0071 | NIWA85184 | Ross Sea | 91 |  | 2008 | TAN0802 |  | 61 | -75.621667 | 169.8045 | 521 |
| Ophionotus | victoriae | N0076 | NIWA85184 | Ross Sea | 91 |  | 2008 | TAN0802 |  | 61 | -75.621667 | 169.8045 | 521 |
| Ophionotus | victoriae | N0109 | NIWA84672 | Ross Sea | 91 |  | 2008 | TAN0802 |  | 77 | -76.833333 | 179.95 | 664 |
| Ophionotus | victoriae | Op762_3C_2 |  | Ross Sea | 91 | KY048261 | 2013 | NBP-12-10 | 21 |  | -78.06324 | -169.99115 | 549 |
| Ophionotus | victoriae | Op787_6C_4 |  | Ross Sea | 91 | KY048261 | 2013 | NBP-12-10 | 22 |  | -76.998275 | -175.0932 | 541 |
| Ophionotus | victoriae | Op806_3C_1 |  | Ross Sea | 91 | KY048261 | 2013 | NBP-12-10 | 24 |  | -76.9038 | 169.96525 | 764 |
| Ophionotus | victoriae | Op806_7C |  | Ross Sea | 91 | KY048261 | 2013 | NBP-12-10 | 24 |  | -76.9038 | 169.96525 | 764 |
| Ophionotus | victoriae | Op806_8C_1 |  | Ross Sea | 91 | KY048261 | 2013 | NBP-12-10 | 24 |  | -76.9038 | 169.96525 | 764 |
| Ophionotus | victoriae | Op818_2E |  | Ross Sea | 91 | KY048261 | 2013 | NBP-12-10 | 25 |  | -75.833465 | 166.50549 | 552 |
| Ophionotus | victoriae | Op818_3C_3 |  | Ross Sea | 91 | KY048261 | 2013 | NBP-12-10 | 25 |  | -75.833465 | 166.50549 | 552 |
| Ophionotus | victoriae | Op818_4C_3 |  | Ross Sea | 91 | KY048261 | 2013 | NBP-12-10 | 25 |  | -75.833465 | 166.50549 | 552 |
| Ophionotus | victoriae | Op818_4C_4 |  | Ross Sea | 91 | KY048261 | 2013 | NBP-12-10 | 25 |  | -75.833465 | 166.50549 | 552 |
| Ophionotus | victoriae | Op826_3C_2 |  | Ross Sea | 91 | KY048261 | 2013 | NBP-12-10 | 26 |  | -74.70781 | 168.40783 | 489 |
| Ophionotus | victoriae | Op843_3C_1 |  | Ross Sea | 91 | KY048261 | 2013 | NBP-12-10 | 28 |  | -74.995422 | 165.74422 | 1101 |
| Ophionotus | victoriae | Op843_7C_1 |  | Ross Sea | 91 | KY048261 | 2013 | NBP-12-10 | 28 |  | -74.995422 | 165.74422 | 1101 |
| Ophionotus | victoriae | Op843_7C_4 |  | Ross Sea | 91 | KY048261 | 2013 | NBP-12-10 | 28 |  | -74.995422 | 165.74422 | 1101 |
| Ophionotus | victoriae | N0066 | NIWA85184 | Ross Sea | 94 |  | 2008 | TAN0802 |  | 61 | -75.621667 | 169.8045 | 521 |
| Ophionotus | victoriae | N0074 | NIWA85184 | Ross Sea | 95 |  | 2008 | TAN0802 |  | 61 | -75.621667 | 169.8045 | 521 |
| Ophionotus | victoriae | 94869A | NIWA94869 | Ross Sea | 96 |  | 2004 | TAN0402 |  | 174 | -71.4936676 | 171.6041718 | 483 |
| Ophionotus | victoriae | N0104 | NIWA84672 | Ross Sea | 102 |  | 2008 | TAN0802 |  | 77 | -76.833333 | 179.95 | 664 |
| Ophionotus | victoriae | PDIVA-M | SIOBICE4766 | Ross Sea | 109 |  | 2010 | SIO McMurdo |  | IVA | -77.5717 | 163.5117 |  |
| Ophionotus | victoriae | PDIVA-N | SIOBICE4766 | Ross Sea | 110 |  | 2010 | SIO McMurdo |  | IVA | -77.5717 | 163.5117 |  |
| Ophionotus | victoriae | 93825B | NIWA93825 | Ross Sea | 163 |  | 2004 | TAN0402 |  | 174 | -71.4936676 | 171.6041718 | 483 |
| Ophionotus | victoriae | 94858A | NIWA94858 | Ross Sea | 164 |  | 2004 | TAN0402 |  | 133 | -71.64466858 | 170.2188263 | 252 |
| Ophionotus | victoriae | 94866A | NIWA94866 | Ross Sea | 165 |  | 2004 | TAN0402 |  | 132 | -71.64766693 | 170.1801605 | 172 |
| Ophionotus | victoriae | N0111 | NIWA84671 | Scott Island | 24 |  | 2008 | TAN0802 |  | 223 | -67.828833 | -179.587 | 403 |
| Ophionotus | victoriae | N0114 | NIWA84671 | Scott Island | 24 |  | 2008 | TAN0802 |  | 223 | -67.828833 | -179.587 | 403 |
| Ophionotus | victoriae | N0115 | NIWA84671 | Scott Island | 24 |  | 2008 | TAN0802 |  | 223 | -67.828833 | -179.587 | 403 |
| Ophionotus | victoriae | N0116 | NIWA84671 | Scott Island | 24 |  | 2008 | TAN0802 |  | 223 | -67.828833 | -179.587 | 403 |
| Ophionotus | victoriae | N0119 | NIWA84671 | Scott Island | 24 |  | 2008 | TAN0802 |  | 223 | -67.828833 | -179.587 | 403 |
| Ophionotus | victoriae | N0120 | NIWA84671 | Scott Island | 24 |  | 2008 | TAN0802 |  | 223 | -67.828833 | -179.587 | 403 |
| Ophionotus | victoriae | N0121 | NIWA84671 | Scott Island | 24 |  | 2008 | TAN0802 |  | 223 | -67.828833 | -179.587 | 403 |
| Ophionotus | victoriae | N0123 | NIWA84675 | Scott Island | 24 |  | 2008 | TAN0802 |  | 247 | -67.3875 | -179.897167 | 144 |
| Ophionotus | victoriae | N0124 | NIWA84675 | Scott Island | 24 |  | 2008 | TAN0802 |  | 247 | -67.3875 | -179.897167 | 144 |
| Ophionotus | victoriae | N0125 | NIWA84675 | Scott Island | 24 |  | 2008 | TAN0802 |  | 247 | -67.3875 | -179.897167 | 144 |
| Ophionotus | victoriae | N0126 | NIWA84675 | Scott Island | 24 |  | 2008 | TAN0802 |  | 247 | -67.3875 | -179.897167 | 144 |
| Ophionotus | victoriae | N0127 | NIWA84675 | Scott Island | 24 |  | 2008 | TAN0802 |  | 247 | -67.3875 | -179.897167 | 144 |
| Ophionotus | victoriae | N0128 | NIWA84675 | Scott Island | 24 |  | 2008 | TAN0802 |  | 247 | -67.3875 | -179.897167 | 144 |
| Ophionotus | victoriae | N0129 | NIWA84675 | Scott Island | 24 |  | 2008 | TAN0802 |  | 247 | -67.3875 | -179.897167 | 144 |
| Ophionotus | victoriae | N0133 | NIWA84675 | Scott Island | 24 |  | 2008 | TAN0802 |  | 247 | -67.3875 | -179.897167 | 144 |
| Ophionotus | victoriae | N0134 | NIWA84675 | Scott Island | 24 |  | 2008 | TAN0802 |  | 247 | -67.3875 | -179.897167 | 144 |
| Ophionotus | victoriae | N0113 | NIWA84671 | Scott Island | 46 |  | 2008 | TAN0802 |  | 223 | -67.828833 | -179.587 | 403 |
| Ophionotus | victoriae | N0132 | NIWA84675 | Scott Island | 46 |  | 2008 | TAN0802 |  | 247 | -67.3875 | -179.897167 | 144 |
| Ophionotus | victoriae | N0136 | NIWA84675 | Scott Island | 100 |  | 2008 | TAN0802 |  | 247 | -67.3875 | -179.897167 | 144 |
| Ophionotus | victoriae | N0112 | NIWA84671 | Scott Island | 103 |  | 2008 | TAN0802 |  | 223 | -67.828833 | -179.587 | 403 |
| Ophionotus | victoriae | N0118 | NIWA84671 | Scott Island | 104 |  | 2008 | TAN0802 |  | 223 | -67.828833 | -179.587 | 403 |
| Ophionotus | victoriae | N0122 | NIWA84671 | Scott Island | 105 |  | 2008 | TAN0802 |  | 223 | -67.828833 | -179.587 | 403 |
| Ophionotus | victoriae | N0130 | NIWA84675 | Scott Island | 106 |  | 2008 | TAN0802 |  | 247 | -67.3875 | -179.897167 | 144 |
| Ophionotus | victoriae | N0131 | NIWA84675 | Scott Island | 107 |  | 2008 | TAN0802 |  | 247 | -67.3875 | -179.897167 | 144 |
| Ophionotus | victoriae | N0138 | NIWA84675 | Scott Island | 108 |  | 2008 | TAN0802 |  | 247 | -67.3875 | -179.897167 | 144 |
| Ophionotus | victoriae | DSOPH1908 |  | Shetland Islands | 1 | FJ917337 | 2006 | JR144 | EI-AGT-4 |  | -61.334 | -55.195 | 201 |
| Ophionotus | victoriae | DSOPH2154 |  | Shetland Islands | 1 | FJ917337 | 2006 | JR144 | ST-EBS-4 |  | -59.47 | -27.276 | 308 |
| Ophionotus | victoriae | DSOPH2327 |  | Shetland Islands | 1 | FJ917339 | 2006 | JR144 | EI-AGT-3 |  | -61.386 | -55.193 | 483 |
| Ophionotus | victoriae | DSOPH721 |  | Shetland Islands | 1 | FJ917337 | 2006 | JR144 | EI-AGT-3 |  | -61.386 | -55.193 | 483 |
| Ophionotus | victoriae | DSOPH722 |  | Shetland Islands | 1 | FJ917337 | 2006 | JR144 | EI-AGT-3 |  | -61.386 | -55.193 | 483 |
| Ophionotus | victoriae | DSOPH724 |  | Shetland Islands | 1 | FJ917339 | 2006 | JR144 | EI-AGT-3 |  | -61.386 | -55.193 | 483 |
| Ophionotus | victoriae | DSOPH725 |  | Shetland Islands | 1 | FJ917337 | 2006 | JR144 | EI-AGT-3 |  | -61.386 | -55.193 | 483 |
| Ophionotus | victoriae | DSOPH1756 |  | Shetland Islands | 3 | FJ917340 | 2006 | JR144 | EI-AGT-4 |  | -61.334 | -55.195 | 201 |
| Ophionotus | victoriae | DSOPH2155 |  | Shetland Islands | 3 | FJ917340 | 2006 | JR144 | LI-AGT-4 |  | -62.525 | -61.827 | 193 |
| Ophionotus | victoriae | SIOBICS5741E | SIOBICE7575 | Shetland Islands | 4 |  | 2011 | Scotia 2011 | SSH1 | 94 | -62.33746 | -60.74441 | 183 |
| Ophionotus | victoriae | 114.3C |  | Shetland Islands | 5 | FJ917320 | 2004 | LMG-04-14 | 64 |  | -62.933694 | -60.65995 | 161 |
| Ophionotus | victoriae | E73.2C.10 |  | Shetland Islands | 5 | FJ917320 | 2004 | LMG-04-14 | 44 |  | -62.100361 | -58.393283 | 276 |
| Ophionotus | victoriae | SIOBICS5741B | SIOBICE7575 | Shetland Islands | 5 |  | 2011 | Scotia 2011 | SSH1 | 94 | -62.33746 | -60.74441 | 183 |
| Ophionotus | victoriae | SIOBICS5741H | SIOBICE7575 | Shetland Islands | 5 |  | 2011 | Scotia 2011 | SSH1 | 94 | -62.33746 | -60.74441 | 183 |
| Ophionotus | victoriae | AP46 (haplotype ID) |  | Shetland Islands | 6 | FJ917352 |  | Laurence M. Gould |  |  | -62.283333 | -58.45 | 192 |
| Ophionotus | victoriae | DSOPH2156 |  | Shetland Islands | 6 | FJ917309 | 2006 | JR144 | LI-AGT-4 |  | -62.525 | -61.827 | 193 |
| Ophionotus | victoriae | E73.2C.01 |  | Shetland Islands | 6 | FJ917309 | 2004 | LMG-04-14 | 44 |  | -62.100361 | -58.393283 | 276 |
| Ophionotus | victoriae | E73.2C.05 |  | Shetland Islands | 6 | FJ917309 | 2004 | LMG-04-14 | 44 |  | -62.100361 | -58.393283 | 276 |
| Ophionotus | victoriae | SIOBICS5741K | SIOBICE7575 | Shetland Islands | 6 |  | 2011 | Scotia 2011 | SSH1 | 94 | -62.33746 | -60.74441 | 183 |
| Ophionotus | victoriae | SIOBICS5741O | SIOBICE7575 | Shetland Islands | 6 |  | 2011 | Scotia 2011 | SSH1 | 94 | -62.33746 | -60.74441 | 183 |
| Ophionotus | victoriae | SIOBICS5741P | SIOBICE7575 | Shetland Islands | 6 |  | 2011 | Scotia 2011 | SSH1 | 94 | -62.33746 | -60.74441 | 183 |
| Ophionotus | victoriae | SIOBICS5743 | SIOBICE5023 | Shetland Islands | 6 |  | 2011 | Scotia 2011 | SSH1 | 94 | -62.33746 | -60.74441 | 183 |
| Ophionotus | victoriae | 114.13C |  | Shetland Islands | 7 | FJ917322 | 2004 | LMG-04-14 | 64 |  | -62.933694 | -60.65995 | 161 |
| Ophionotus | victoriae | 114.6C |  | Shetland Islands | 7 | FJ917322 | 2004 | LMG-04-14 | 64 |  | -62.933694 | -60.65995 | 161 |
| Ophionotus | victoriae | DSOPH1910 |  | Shetland Islands | 7 | FJ917322 | 2006 | JR144 | EI-AGT-4 |  | -61.334 | -55.195 | 201 |
| Ophionotus | victoriae | E73.2C.06 |  | Shetland Islands | 7 | FJ917322 | 2004 | LMG-04-14 | 44 |  | -62.100361 | -58.393283 | 276 |
| Ophionotus | victoriae | SIOBICS5741C | SIOBICE7575 | Shetland Islands | 7 |  | 2011 | Scotia 2011 | SSH1 | 94 | -62.33746 | -60.74441 | 183 |
| Ophionotus | victoriae | SIOBICS5741I | SIOBICE7575 | Shetland Islands | 7 |  | 2011 | Scotia 2011 | SSH1 | 94 | -62.33746 | -60.74441 | 183 |
| Ophionotus | victoriae | 114.10C |  | Shetland Islands | 8 | FJ917316 | 2004 | LMG-04-14 | 64 |  | -62.933694 | -60.65995 | 161 |
| Ophionotus | victoriae | 114.4C |  | Shetland Islands | 8 | FJ917316 | 2004 | LMG-04-14 | 64 |  | -62.933694 | -60.65995 | 161 |
| Ophionotus | victoriae | 114.7C |  | Shetland Islands | 8 | FJ917316 | 2004 | LMG-04-14 | 64 |  | -62.933694 | -60.65995 | 161 |
| Ophionotus | victoriae | 114.8C |  | Shetland Islands | 8 | FJ917316 | 2004 | LMG-04-14 | 64 |  | -62.933694 | -60.65995 | 161 |
| Ophionotus | victoriae | E73.2C.09 |  | Shetland Islands | 8 | FJ917316 | 2004 | LMG-04-14 | 44 |  | -62.100361 | -58.393283 | 276 |
| Ophionotus | victoriae | SIOBICS5741N | SIOBICE7575 | Shetland Islands | 8 |  | 2011 | Scotia 2011 | SSH1 | 94 | -62.33746 | -60.74441 | 183 |
| Ophionotus | victoriae | 114.5C |  | Shetland Islands | 10 | FJ917312 | 2004 | LMG-04-14 | 64 |  | -62.933694 | -60.65995 | 161 |
| Ophionotus | victoriae | DSOPH1909 |  | Shetland Islands | 10 | FJ917312 | 2006 | JR144 | EI-AGT-4 |  | -61.334 | -55.195 | 201 |
| Ophionotus | victoriae | DSOPH1912 |  | Shetland Islands | 14 | FJ917310 | 2006 | JR144 | EI-AGT-4 |  | -61.334 | -55.195 | 201 |
| Ophionotus | victoriae | DSOPH2161 |  | Shetland Islands | 14 | FJ917310 | 2006 | JR144 | LI-AGT-1 |  | -62.276 | -61.596 | 1511 |
| Ophionotus | victoriae | SIOBICS5741Q | SIOBICE7575 | Shetland Islands | 14 |  | 2011 | Scotia 2011 | SSH1 | 94 | -62.33746 | -60.74441 | 183 |
| Ophionotus | victoriae | SIOBICS5742 | SIOBICE5171 | Shetland Islands | 14 |  | 2011 | Scotia 2011 | SSH1 | 94 | -62.33746 | -60.74441 | 183 |
| Ophionotus | victoriae | AP44 (haplotype ID) |  | Shetland Islands | 24 | FJ917350 | 2008 | JR179 | BIO6-AGT-2A |  | -62.283333 | -58.45 | 192 |
| Ophionotus | victoriae | E73.2C.02 |  | Shetland Islands | 24 | FJ917318 | 2004 | LMG-04-14 | 44 |  | -62.100361 | -58.393283 | 276 |
| Ophionotus | victoriae | E73.2C.11 |  | Shetland Islands | 24 | FJ917318 | 2004 | LMG-04-14 | 44 |  | -62.100361 | -58.393283 | 276 |
| Ophionotus | victoriae | SIOBICS5741M | SIOBICE7575 | Shetland Islands | 24 |  | 2011 | Scotia 2011 | SSH1 | 94 | -62.33746 | -60.74441 | 183 |
| Ophionotus | victoriae | DSOPH1898 |  | Shetland Islands | 25 | FJ917319 | 2006 | JR144 | EI-AGT-4 |  | -61.334 | -55.195 | 201 |
| Ophionotus | victoriae | E73.2C.08 |  | Shetland Islands | 25 | FJ917319 | 2004 | LMG-04-14 | 44 |  | -62.100361 | -58.393283 | 276 |
| Ophionotus | victoriae | SIOBICS5741R | SIOBICE7575 | Shetland Islands | 25 |  | 2011 | Scotia 2011 | SSH1 | 94 | -62.33746 | -60.74441 | 183 |
| Ophionotus | victoriae | 114.2C |  | Shetland Islands | 26 | FJ917321 | 2004 | LMG-04-14 | 64 |  | -62.933694 | -60.65995 | 161 |
| Ophionotus | victoriae | 114.11C |  | Shetland Islands | 27 | FJ917323 | 2004 | LMG-04-14 | 64 |  | -62.933694 | -60.65995 | 161 |
| Ophionotus | victoriae | SIOBICS5741F | SIOBICE7575 | Shetland Islands | 32 |  | 2011 | Scotia 2011 | SSH1 | 94 | -62.33746 | -60.74441 | 183 |
| Ophionotus | victoriae | SIOBICS5741A | SIOBICE7575 | Shetland Islands | 39 |  | 2011 | Scotia 2011 | SSH1 | 94 | -62.33746 | -60.74441 | 183 |
| Ophionotus | victoriae | SIOBICS5741D | SIOBICE7575 | Shetland Islands | 39 |  | 2011 | Scotia 2011 | SSH1 | 94 | -62.33746 | -60.74441 | 183 |
| Ophionotus | victoriae | SIOBICS5741G | SIOBICE7575 | Shetland Islands | 40 |  | 2011 | Scotia 2011 | SSH1 | 94 | -62.33746 | -60.74441 | 183 |
| Ophionotus | victoriae | SIOBICS5741J | SIOBICE7575 | Shetland Islands | 40 |  | 2011 | Scotia 2011 | SSH1 | 94 | -62.33746 | -60.74441 | 183 |
| Ophionotus | victoriae | E73.2C.03 |  | Shetland Islands | 45 | FJ917351 | 2004 | LMG-04-14 | 44 |  | -62.100361 | -58.393283 | 276 |
| Ophionotus | victoriae | E73.2C.12 |  | Shetland Islands | 46 | FJ917353 | 2004 | LMG-04-14 | 44 |  | -62.100361 | -58.393283 | 276 |
| Ophionotus | victoriae | DSOPH1899 |  | Shetland Islands | 55 | KY048218 | 2006 | JR144 | EI-AGT-4 |  | -61.334 | -55.195 | 201 |
| Ophionotus | victoriae | DSOPH1900 |  | Shetland Islands | 55 | KY048218 | 2006 | JR144 | EI-AGT-4 |  | -61.334 | -55.195 | 201 |
| Ophionotus | victoriae | DSOPH1911 |  | Shetland Islands | 56 | KY048219 | 2006 | JR144 | EI-AGT-4 |  | -61.334 | -55.195 | 201 |
| Ophionotus | victoriae | DSOPH2157 |  | Shetland Islands | 60 | KY048223 | 2006 | JR144 | RGBT-02 |  | -61.966 | -57.244 | 129.76 |
| Ophionotus | victoriae | DSOPH3528 |  | Shetland Islands | 83 | KY048252 | 2011 | Polarstern ANT-XXVII/3 | PS77_222-5 |  | -62.297002 | -58.678 | 873 |
| Ophionotus | victoriae | SIOBICS5741L | SIOBICE7575 | Shetland Islands | 136 |  | 2011 | Scotia 2011 | SSH1 | 94 | -62.33746 | -60.74441 | 183 |
| Ophionotus | victoriae | SIOBICS5741S | SIOBICE7575 | Shetland Islands | 137 |  | 2011 | Scotia 2011 | SSH1 | 94 | -62.33746 | -60.74441 | 183 |
| Ophionotus | victoriae | SIOBICS20231 | SIOBICE6408 | South Georgia | 18 |  | 2013 | Scotia 2013 | SG4b | 9 | -53.633817 | -37.307067 | 167 |
| Ophionotus | victoriae | SIOBICS20237 | SIOBICE6420 | South Georgia | 19 |  | 2013 | Scotia 2013 | SG4 | 5 | -53.715167 | -36.83575 | 190 |
| Ophionotus | victoriae | DSOPH1904 |  | South Orkney Island | 31 | FJ917328 | 2006 | JR144 | PB-AGT-1B |  | -61.036 | -46.955 | 1630 |
| Ophionotus | victoriae | SIOBICS0990J | SIOBICE4784 | South Sandwich Islands | 10 |  | 2011 | Scotia 2011 | SS1 | 25 | -57.03366 | -26.75914 | 118 |
| Ophionotus | victoriae | DSOPH2160 |  | South Sandwich Islands | 14 | KY048225 | 2006 | JR144 | LI-AGT-1 |  | -62.276 | -61.596 | 1511 |
| Ophionotus | victoriae | 194.1E.01 |  | South Sandwich Islands | 31 | FJ917328 | 2004 | LMG-04-14 | 34 |  | -58.783694 | -26.343283 | 270 |
| Ophionotus | victoriae | 194.1E.06 |  | South Sandwich Islands | 31 | FJ917328 | 2004 | LMG-04-14 | 34 |  | -58.783694 | -26.343283 | 270 |
| Ophionotus | victoriae | 194.1E.09 |  | South Sandwich Islands | 31 | FJ917335 | 2004 | LMG-04-14 | 34 |  | -58.783694 | -26.343283 | 270 |
| Ophionotus | victoriae | 195.1E.03 |  | South Sandwich Islands | 31 | FJ917328 | 2006 | LMG-04-14 | 32 |  | -57.088533 | -30.398971 | 130 |
| Ophionotus | victoriae | 195.1E.06 |  | South Sandwich Islands | 31 | FJ917328 | 2006 | LMG-04-14 | 32 |  | -57.088533 | -30.398971 | 130 |
| Ophionotus | victoriae | 195.1E.08 |  | South Sandwich Islands | 31 | FJ917328 | 2006 | LMG-04-14 | 32 |  | -57.088533 | -30.398971 | 130 |
| Ophionotus | victoriae | DSOPH2149 |  | South Sandwich Islands | 31 | FJ917328 | 2006 | JR144 | ST-AGT-1 |  | -59.518 | -27.436 | 1545 |
| Ophionotus | victoriae | SIOBICS0539A | SIOBICE4802 | South Sandwich Islands | 31 |  | 2011 | Scotia 2011 | SS2 | 33 | -58.475 | -26.205 | 161 |
| Ophionotus | victoriae | SIOBICS0539C | SIOBICE4802 | South Sandwich Islands | 31 |  | 2011 | Scotia 2011 | SS2 | 33 | -58.475 | -26.205 | 161 |
| Ophionotus | victoriae | SIOBICS0539E | SIOBICE4802 | South Sandwich Islands | 31 |  | 2011 | Scotia 2011 | SS2 | 33 | -58.475 | -26.205 | 161 |
| Ophionotus | victoriae | SIOBICS0539F | SIOBICE4802 | South Sandwich Islands | 31 |  | 2011 | Scotia 2011 | SS2 | 33 | -58.475 | -26.205 | 161 |
| Ophionotus | victoriae | SIOBICS0539L | SIOBICE4802 | South Sandwich Islands | 31 |  | 2011 | Scotia 2011 | SS2 | 33 | -58.475 | -26.205 | 161 |
| Ophionotus | victoriae | SIOBICS0539M | SIOBICE4802 | South Sandwich Islands | 31 |  | 2011 | Scotia 2011 | SS2 | 33 | -58.475 | -26.205 | 161 |
| Ophionotus | victoriae | SIOBICS0539Q | SIOBICE4802 | South Sandwich Islands | 31 |  | 2011 | Scotia 2011 | SS2 | 33 | -58.475 | -26.205 | 161 |
| Ophionotus | victoriae | SIOBICS0539R | SIOBICE4802 | South Sandwich Islands | 31 |  | 2011 | Scotia 2011 | SS2 | 33 | -58.475 | -26.205 | 161 |
| Ophionotus | victoriae | SIOBICS0539S | SIOBICE4802 | South Sandwich Islands | 31 |  | 2011 | Scotia 2011 | SS2 | 33 | -58.475 | -26.205 | 161 |
| Ophionotus | victoriae | SIOBICS0539T | SIOBICE4802 | South Sandwich Islands | 31 |  | 2011 | Scotia 2011 | SS2 | 33 | -58.475 | -26.205 | 161 |
| Ophionotus | victoriae | SIOBICS0554N | SIOBICE4786 | South Sandwich Islands | 31 |  | 2011 | Scotia 2011 | SS3 | 41 | -59.39364 | -27.32269 | 110 |
| Ophionotus | victoriae | SIOBICS0554P | SIOBICE4786 | South Sandwich Islands | 31 |  | 2011 | Scotia 2011 | SS3 | 41 | -59.39364 | -27.32269 | 110 |
| Ophionotus | victoriae | SIOBICS0554Q | SIOBICE4786 | South Sandwich Islands | 31 |  | 2011 | Scotia 2011 | SS3 | 41 | -59.39364 | -27.32269 | 110 |
| Ophionotus | victoriae | SIOBICS0554R | SIOBICE4786 | South Sandwich Islands | 31 |  | 2011 | Scotia 2011 | SS3 | 41 | -59.39364 | -27.32269 | 110 |
| Ophionotus | victoriae | SIOBICS0554S | SIOBICE4786 | South Sandwich Islands | 31 |  | 2011 | Scotia 2011 | SS3 | 41 | -59.39364 | -27.32269 | 110 |
| Ophionotus | victoriae | SIOBICS0990A | SIOBICE4784 | South Sandwich Islands | 31 |  | 2011 | Scotia 2011 | SS1 | 25 | -57.03366 | -26.75914 | 118 |
| Ophionotus | victoriae | SIOBICS0990D | SIOBICE4784 | South Sandwich Islands | 31 |  | 2011 | Scotia 2011 | SS1 | 25 | -57.03366 | -26.75914 | 118 |
| Ophionotus | victoriae | SIOBICS0990L | SIOBICE4784 | South Sandwich Islands | 31 |  | 2011 | Scotia 2011 | SS1 | 25 | -57.03366 | -26.75914 | 118 |
| Ophionotus | victoriae | SIOBICS0990M | SIOBICE4784 | South Sandwich Islands | 31 |  | 2011 | Scotia 2011 | SS1 | 25 | -57.03366 | -26.75914 | 118 |
| Ophionotus | victoriae | SIOBICS0990P | SIOBICE4784 | South Sandwich Islands | 31 |  | 2011 | Scotia 2011 | SS1 | 25 | -57.03366 | -26.75914 | 118 |
| Ophionotus | victoriae | SIOBICS0990T | SIOBICE4784 | South Sandwich Islands | 31 |  | 2011 | Scotia 2011 | SS1 | 25 | -57.03366 | -26.75914 | 118 |
| Ophionotus | victoriae | WAMZ44932 | WAMZ44932 | South Sandwich Islands | 31 |  | 2017 | ACE 2016/17 | 90 | 2590 | -59.4718 | -27.264 | 230 |
| Ophionotus | victoriae | WAMZ44934 | WAMZ44934 | South Sandwich Islands | 31 |  | 2017 | ACE 2016/17 | 90 | 2590 | -59.4718 | -27.264 | 230 |
| Ophionotus | victoriae | WAMZ44935 | WAMZ44935 | South Sandwich Islands | 31 |  | 2017 | ACE 2016/17 | 90 | 2590 | -59.4718 | -27.264 | 230 |
| Ophionotus | victoriae | WAMZ44936 | WAMZ44936 | South Sandwich Islands | 31 |  | 2017 | ACE 2016/17 | 90 | 2590 | -59.4718 | -27.264 | 230 |
| Ophionotus | victoriae | WAMZ44938 | WAMZ44938 | South Sandwich Islands | 31 |  | 2017 | ACE 2016/17 | 90 | 2590 | -59.4718 | -27.264 | 230 |
| Ophionotus | victoriae | WAMZ44939 | WAMZ44939 | South Sandwich Islands | 31 |  | 2017 | ACE 2016/17 | 90 | 2590 | -59.4718 | -27.264 | 230 |
| Ophionotus | victoriae | WAMZ44941 | WAMZ44941 | South Sandwich Islands | 31 |  | 2017 | ACE 2016/17 | 90 | 2590 | -59.4718 | -27.264 | 230 |
| Ophionotus | victoriae | WAMZ44942 | WAMZ44942 | South Sandwich Islands | 31 |  | 2017 | ACE 2016/17 | 90 | 2590 | -59.4718 | -27.264 | 230 |
| Ophionotus | victoriae | WAMZ44944 | WAMZ44944 | South Sandwich Islands | 31 |  | 2017 | ACE 2016/17 | 90 | 2590 | -59.4718 | -27.264 | 230 |
| Ophionotus | victoriae | WAMZ44945 | WAMZ44945 | South Sandwich Islands | 31 |  | 2017 | ACE 2016/17 | 90 | 2590 | -59.4718 | -27.264 | 230 |
| Ophionotus | victoriae | WAMZ44946 | WAMZ44946 | South Sandwich Islands | 31 |  | 2017 | ACE 2016/17 | 90 | 2590 | -59.4718 | -27.264 | 230 |
| Ophionotus | victoriae | 194.1E.02 |  | South Sandwich Islands | 32 | FJ917329 | 2004 | LMG-04-14 | 34 |  | -58.783694 | -26.343283 | 270 |
| Ophionotus | victoriae | 194.1E.05 |  | South Sandwich Islands | 32 | FJ917332 | 2004 | LMG-04-14 | 34 |  | -58.783694 | -26.343283 | 270 |
| Ophionotus | victoriae | 195.1E.05 |  | South Sandwich Islands | 32 | FJ917329 | 2006 | LMG-04-14 | 32 |  | -57.088533 | -30.398971 | 130 |
| Ophionotus | victoriae | 195.1E.07 |  | South Sandwich Islands | 32 | FJ917332 | 2006 | LMG-04-14 | 32 |  | -57.088533 | -30.398971 | 130 |
| Ophionotus | victoriae | DSOPH1903 |  | South Sandwich Islands | 32 | FJ917329 | 2006 | JR144 | ST-AGT-3 |  | -59.481 | -27.279 | 550 |
| Ophionotus | victoriae | DSOPH2142 |  | South Sandwich Islands | 32 | FJ917329 | 2006 | JR144 | ST-AGT-3 |  | -59.481 | -27.279 | 550 |
| Ophionotus | victoriae | DSOPH2143 |  | South Sandwich Islands | 32 | FJ917329 | 2006 | JR144 | ST-AGT-1 |  | -59.518 | -27.436 | 1545 |
| Ophionotus | victoriae | DSOPH2144 |  | South Sandwich Islands | 32 | FJ917329 | 2006 | JR144 | ST-AGT-1 |  | -59.518 | -27.436 | 1545 |
| Ophionotus | victoriae | DSOPH2145 |  | South Sandwich Islands | 32 | FJ917332 | 2006 | JR144 | ST-AGT-1 |  | -59.518 | -27.436 | 1545 |
| Ophionotus | victoriae | DSOPH2148 |  | South Sandwich Islands | 32 | FJ917329 | 2006 | JR144 | ST-AGT-1 |  | -59.518 | -27.436 | 1545 |
| Ophionotus | victoriae | DSOPH2152 |  | South Sandwich Islands | 32 | FJ917329 | 2006 | JR144 | ST-AGT-1 |  | -59.518 | -27.436 | 1545 |
| Ophionotus | victoriae | SIOBICS0185 | SIOBICE5232 | South Sandwich Islands | 32 |  | 2011 | Scotia 2011 | SS1A | 30 | -56.72348 | -27.03589 | 134 |
| Ophionotus | victoriae | SIOBICS0554T | SIOBICE4786 | South Sandwich Islands | 32 |  | 2011 | Scotia 2011 | SS3 | 41 | -59.39364 | -27.32269 | 110 |
| Ophionotus | victoriae | SIOBICS0594A | SIOBICE4790 | South Sandwich Islands | 32 |  | 2011 | Scotia 2011 | SS3a | 43 | -59.3831 | -27.34543 | 926 |
| Ophionotus | victoriae | SIOBICS0594B | SIOBICE4790 | South Sandwich Islands | 32 |  | 2011 | Scotia 2011 | SS3a | 43 | -59.3831 | -27.34543 | 926 |
| Ophionotus | victoriae | SIOBICS0594C | SIOBICE4790 | South Sandwich Islands | 32 |  | 2011 | Scotia 2011 | SS3a | 43 | -59.3831 | -27.34543 | 926 |
| Ophionotus | victoriae | SIOBICS0594E | SIOBICE4790 | South Sandwich Islands | 32 |  | 2011 | Scotia 2011 | SS3a | 43 | -59.3831 | -27.34543 | 926 |
| Ophionotus | victoriae | SIOBICS0594G | SIOBICE4790 | South Sandwich Islands | 32 |  | 2011 | Scotia 2011 | SS3a | 43 | -59.3831 | -27.34543 | 926 |
| Ophionotus | victoriae | SIOBICS0594H | SIOBICE4790 | South Sandwich Islands | 32 |  | 2011 | Scotia 2011 | SS3a | 43 | -59.3831 | -27.34543 | 926 |
| Ophionotus | victoriae | SIOBICS0990G | SIOBICE4784 | South Sandwich Islands | 32 |  | 2011 | Scotia 2011 | SS1 | 25 | -57.03366 | -26.75914 | 118 |
| Ophionotus | victoriae | SIOBICS0990I | SIOBICE4784 | South Sandwich Islands | 32 |  | 2011 | Scotia 2011 | SS1 | 25 | -57.03366 | -26.75914 | 118 |
| Ophionotus | victoriae | SIOBICS0990Q | SIOBICE4784 | South Sandwich Islands | 32 |  | 2011 | Scotia 2011 | SS1 | 25 | -57.03366 | -26.75914 | 118 |
| Ophionotus | victoriae | WAMZ44590 | WAMZ44590 | South Sandwich Islands | 32 |  | 2017 | ACE 2016/17 | 90 | 2590 | -59.4718 | -27.264 | 230 |
| Ophionotus | victoriae | WAMZ44937 | WAMZ44937 | South Sandwich Islands | 32 |  | 2017 | ACE 2016/17 | 90 | 2590 | -59.4718 | -27.264 | 230 |
| Ophionotus | victoriae | WAMZ44940 | WAMZ44940 | South Sandwich Islands | 32 |  | 2017 | ACE 2016/17 | 90 | 2590 | -59.4718 | -27.264 | 230 |
| Ophionotus | victoriae | WAMZ44943 | WAMZ44943 | South Sandwich Islands | 32 |  | 2017 | ACE 2016/17 | 90 | 2590 | -59.4718 | -27.264 | 230 |
| Ophionotus | victoriae | 194.1E.03 |  | South Sandwich Islands | 33 | FJ917330 | 2004 | LMG-04-14 | 34 |  | -58.783694 | -26.343283 | 270 |
| Ophionotus | victoriae | 194.1E.04 |  | South Sandwich Islands | 34 | FJ917331 | 2004 | LMG-04-14 | 34 |  | -58.783694 | -26.343283 | 270 |
| Ophionotus | victoriae | SIOBICS0539J | SIOBICE4802 | South Sandwich Islands | 34 |  | 2011 | Scotia 2011 | SS2 | 33 | -58.475 | -26.205 | 161 |
| Ophionotus | victoriae | 194.1E.07 |  | South Sandwich Islands | 35 | FJ917333 | 2004 | LMG-04-14 | 34 |  | -58.783694 | -26.343283 | 270 |
| Ophionotus | victoriae | 195.1E.04 |  | South Sandwich Islands | 35 | FJ917333 | 2004 | LMG-04-14 | 32 |  | -57.088533 | -30.398971 | 130 |
| Ophionotus | victoriae | SIOBICS0539I | SIOBICE4802 | South Sandwich Islands | 35 |  | 2011 | Scotia 2011 | SS2 | 33 | -58.475 | -26.205 | 161 |
| Ophionotus | victoriae | SIOBICS0554K | SIOBICE4786 | South Sandwich Islands | 35 |  | 2011 | Scotia 2011 | SS3 | 41 | -59.39364 | -27.32269 | 110 |
| Ophionotus | victoriae | SIOBICS0990B | SIOBICE4784 | South Sandwich Islands | 35 |  | 2011 | Scotia 2011 | SS1 | 25 | -57.03366 | -26.75914 | 118 |
| Ophionotus | victoriae | WAMZ44933 | WAMZ44933 | South Sandwich Islands | 35 |  | 2017 | ACE 2016/17 | 90 | 2590 | -59.4718 | -27.264 | 230 |
| Ophionotus | victoriae | 194.1E.08 |  | South Sandwich Islands | 36 | FJ917334 | 2004 | LMG-04-14 | 34 |  | -58.783694 | -26.343283 | 270 |
| Ophionotus | victoriae | SIOBICS0539N | SIOBICE4802 | South Sandwich Islands | 36 |  | 2011 | Scotia 2011 | SS2 | 33 | -58.475 | -26.205 | 161 |
| Ophionotus | victoriae | SIOBICS0539O | SIOBICE4802 | South Sandwich Islands | 36 |  | 2011 | Scotia 2011 | SS2 | 33 | -58.475 | -26.205 | 161 |
| Ophionotus | victoriae | SIOBICS0554M | SIOBICE4786 | South Sandwich Islands | 36 |  | 2011 | Scotia 2011 | SS3 | 41 | -59.39364 | -27.32269 | 110 |
| Ophionotus | victoriae | SIOBICS0554O | SIOBICE4786 | South Sandwich Islands | 36 |  | 2011 | Scotia 2011 | SS3 | 41 | -59.39364 | -27.32269 | 110 |
| Ophionotus | victoriae | SIOBICS0990R | SIOBICE4784 | South Sandwich Islands | 36 |  | 2011 | Scotia 2011 | SS1 | 25 | -57.03366 | -26.75914 | 118 |
| Ophionotus | victoriae | DSOPH2146 |  | South Sandwich Islands | 57 | KY048220 | 2006 | JR144 | ST-AGT-1 |  | -59.518 | -27.436 | 1545 |
| Ophionotus | victoriae | DSOPH2150 |  | South Sandwich Islands | 58 | KY048221 | 2006 | JR144 | ST-AGT-1 |  | -59.518 | -27.436 | 1545 |
| Ophionotus | victoriae | DSOPH2151 |  | South Sandwich Islands | 59 | KY048222 | 2006 | JR144 | ST-AGT-1 |  | -59.518 | -27.436 | 1545 |
| Ophionotus | victoriae | DSOPH2159 |  | South Sandwich Islands | 60 | KY048223 | 2006 | JR144 | LI-AGT-1 |  | -62.276 | -61.596 | 1511 |
| Ophionotus | victoriae | DSOPH2158 |  | South Sandwich Islands | 61 | KY048224 | 2006 | JR144 | LI-AGT-1 |  | -62.276 | -61.596 | 1511 |
| Ophionotus | victoriae | SIOBICS0460 | SIOBICE5187 | South Sandwich Islands | 113 |  | 2011 | Scotia 2011 | SS1A | 32 | -56.70875 | -27.04858 | 116 |
| Ophionotus | victoriae | SIOBICS0539B | SIOBICE4802 | South Sandwich Islands | 114 |  | 2011 | Scotia 2011 | SS2 | 33 | -58.475 | -26.205 | 161 |
| Ophionotus | victoriae | SIOBICS0539D | SIOBICE4802 | South Sandwich Islands | 115 |  | 2011 | Scotia 2011 | SS2 | 33 | -58.475 | -26.205 | 161 |
| Ophionotus | victoriae | SIOBICS0539G | SIOBICE4802 | South Sandwich Islands | 116 |  | 2011 | Scotia 2011 | SS2 | 33 | -58.475 | -26.205 | 161 |
| Ophionotus | victoriae | SIOBICS0539H | SIOBICE4802 | South Sandwich Islands | 117 |  | 2011 | Scotia 2011 | SS2 | 33 | -58.475 | -26.205 | 161 |
| Ophionotus | victoriae | SIOBICS0539K | SIOBICE4802 | South Sandwich Islands | 118 |  | 2011 | Scotia 2011 | SS2 | 33 | -58.475 | -26.205 | 161 |
| Ophionotus | victoriae | SIOBICS0539P | SIOBICE4802 | South Sandwich Islands | 119 |  | 2011 | Scotia 2011 | SS2 | 33 | -58.475 | -26.205 | 161 |
| Ophionotus | victoriae | SIOBICS0554L | SIOBICE4786 | South Sandwich Islands | 120 |  | 2011 | Scotia 2011 | SS3 | 41 | -59.39364 | -27.32269 | 110 |
| Ophionotus | victoriae | SIOBICS0594D | SIOBICE4790 | South Sandwich Islands | 121 |  | 2011 | Scotia 2011 | SS3a | 43 | -59.3831 | -27.34543 | 926 |
| Ophionotus | victoriae | SIOBICS0594I | SIOBICE4790 | South Sandwich Islands | 122 |  | 2011 | Scotia 2011 | SS3a | 43 | -59.3831 | -27.34543 | 926 |
| Ophionotus | victoriae | SIOBICS0594J | SIOBICE4790 | South Sandwich Islands | 123 |  | 2011 | Scotia 2011 | SS3a | 43 | -59.3831 | -27.34543 | 926 |
| Ophionotus | victoriae | SIOBICS0990C | SIOBICE4784 | South Sandwich Islands | 124 |  | 2011 | Scotia 2011 | SS1 | 25 | -57.03366 | -26.75914 | 118 |
| Ophionotus | victoriae | SIOBICS0990E | SIOBICE4784 | South Sandwich Islands | 125 |  | 2011 | Scotia 2011 | SS1 | 25 | -57.03366 | -26.75914 | 118 |
| Ophionotus | victoriae | SIOBICS0990F | SIOBICE4784 | South Sandwich Islands | 126 |  | 2011 | Scotia 2011 | SS1 | 25 | -57.03366 | -26.75914 | 118 |
| Ophionotus | victoriae | SIOBICS0990K | SIOBICE4784 | South Sandwich Islands | 127 |  | 2011 | Scotia 2011 | SS1 | 25 | -57.03366 | -26.75914 | 118 |
| Ophionotus | victoriae | SIOBICS0990N | SIOBICE4784 | South Sandwich Islands | 128 |  | 2011 | Scotia 2011 | SS1 | 25 | -57.03366 | -26.75914 | 118 |
| Ophionotus | victoriae | SIOBICS0990O | SIOBICE4784 | South Sandwich Islands | 129 |  | 2011 | Scotia 2011 | SS1 | 25 | -57.03366 | -26.75914 | 118 |
| Ophionotus | victoriae | DSOPH2962 |  | Weddell Sea | 1 | FJ917337 | 2011 | Polarstern ANT-XXVII/3 | PS77_265-2 |  | -70.794 | -10.67 | 634 |
| Ophionotus | victoriae | DSOPH2963 |  | Weddell Sea | 1 | FJ917337 | 2011 | Polarstern ANT-XXVII/3 | PS77_265-2 |  | -70.794 | -10.67 | 634 |
| Ophionotus | victoriae | DSOPH3033 |  | Weddell Sea | 1 | FJ917337 | 2011 | Polarstern ANT-XXVII/3 | PS77_260-6 |  | -70.84 | -10.597 | 260 |
| Ophionotus | victoriae | DSOPH3035 |  | Weddell Sea | 1 | FJ917337 | 2011 | Polarstern ANT-XXVII/3 | PS77_260-6 |  | -70.84 | -10.597 | 260 |
| Ophionotus | victoriae | DSOPH3185 |  | Weddell Sea | 1 | FJ917337 | 2011 | Polarstern ANT-XXVII/3 | PS77_308-1 |  | -70.855 | -10.589 | 224 |
| Ophionotus | victoriae | DSOPH3216 |  | Weddell Sea | 1 | FJ917337 | 2011 | Polarstern ANT-XXVII/3 | PS77_301-1 |  | -70.851 | -10.588 | 226 |
| Ophionotus | victoriae | DSOPH3239 |  | Weddell Sea | 1 | FJ917337 | 2011 | Polarstern ANT-XXVII/3 | PS77_284-1 |  | -70.972 | -10.504 | 290 |
| Ophionotus | victoriae | 308-1.7 | WAMZ88554 | Weddell Sea | 1 |  | 2011 | Polarstern ANT-XXVII/3 | PS77_308-1 |  | -70.858333 | -10.593056 | 250 |
| Ophionotus | victoriae | 265-2.12 | WAMZ88558 | Weddell Sea | 1 |  | 2011 | Polarstern ANT-XXVII/3 | PS77_265-2 |  | -70.792778 | -10.6775 | 615 |
| Ophionotus | victoriae | 265-2.11 | WAMZ88576 | Weddell Sea | 1 |  | 2011 | Polarstern ANT-XXVII/3 | PS77_265-2 |  | -70.792778 | -10.6775 | 615 |
| Ophionotus | victoriae | 308-1.10 | WAMZ88584 | Weddell Sea | 1 |  | 2011 | Polarstern ANT-XXVII/3 | PS77_308-10 |  | -70.858333 | -10.593056 | 250 |
| Ophionotus | victoriae | 1.140.3 | WAMZ88587 | Weddell Sea | 1 |  | 2014 | Polarstern ANT-XXIX/9 | PS82_191-1 |  | -74.665556 | -33.733056 | 592 |
| Ophionotus | victoriae | 1.83.2 | WAMZ88590 | Weddell Sea | 1 |  | 2014 | Polarstern ANT-XXIX/9 | PS82_67-1 |  | -77.101333 | -36.546 | 1101 |
| Ophionotus | victoriae | DSOPH3120 |  | Weddell Sea | 2 | KY048248 | 2011 | Polarstern ANT-XXVII/3 | PS77_286-1 |  | -70.844 | -10.602 | 248 |
| Ophionotus | victoriae | DSOPH2971 |  | Weddell Sea | 3 | FJ917340 | 2011 | Polarstern ANT-XXVII/3 | PS77_265-2 |  | -70.794 | -10.67 | 634 |
| Ophionotus | victoriae | DSOPH3098 |  | Weddell Sea | 3 | FJ917340 | 2011 | Polarstern ANT-XXVII/3 | PS77_291-1 |  | -70.842 | -10.587 | 268 |
| Ophionotus | victoriae | 265-2.10 | WAMZ88570 | Weddell Sea | 3 |  | 2011 | Polarstern ANT-XXVII/3 | PS77_265-2 |  | -70.792778 | -10.6775 | 615 |
| Ophionotus | victoriae | 1.140.4 | WAMZ88588 | Weddell Sea | 14 |  | 2014 | Polarstern ANT-XXIX/9 | PS82_67-1 |  | -77.101333 | -36.546 | 1101 |
| Ophionotus | victoriae | 265-2.11 | WAMZ88559 | Weddell Sea | 24 |  | 2011 | Polarstern ANT-XXVII/3 | PS77_265-2 |  | -70.792778 | -10.6775 | 615 |
| Ophionotus | victoriae | 1.140.1 | WAMZ88585 | Weddell Sea | 24 |  | 2014 | Polarstern ANT-XXIX/9 | PS82_191-1 |  | -74.665556 | -33.733056 | 592 |
| Ophionotus | victoriae | 1.140.2 | WAMZ88586 | Weddell Sea | 24 |  | 2014 | Polarstern ANT-XXIX/9 | PS82_191-1 |  | -74.665556 | -33.733056 | 592 |
| Ophionotus | victoriae | 1.158 | WAMZ88595 | Weddell Sea | 24 |  | 2014 | Polarstern ANT-XXIX/9 | PS82_126-1 |  | -75.512222 | -27.487222 | 282 |
| Ophionotus | victoriae | 1.158.1 | WAMZ88596 | Weddell Sea | 24 |  | 2014 | Polarstern ANT-XXIX/9 | PS82_126-1 |  | -75.512222 | -27.487222 | 282 |
| Ophionotus | victoriae | DSOPH2964 |  | Weddell Sea | 43 | FJ917348 | 2011 | Polarstern ANT-XXVII/3 | PS77_265-2 |  | -70.794 | -10.67 | 634 |
| Ophionotus | victoriae | 265-2.13 | WAMZ88577 | Weddell Sea | 43 |  | 2011 | Polarstern ANT-XXVII/3 | PS77_265-2 |  | -70.792778 | -10.6775 | 615 |
| Ophionotus | victoriae | 1.164.1 | WAMZ88591 | Weddell Sea | 64 |  | 2014 | Polarstern ANT-XXIX/9 | PS82_151-1 |  | -74.540556 | -28.530556 | 1750 |
| Ophionotus | victoriae | 1.152.1 | WAMZ88597 | Weddell Sea | 64 |  | 2014 | Polarstern ANT-XXIX/9 | PS82_115-1 |  | -77.611333 | -38.938833 | 1058 |
| Ophionotus | victoriae | 1.164.2 | WAMZ88592 | Weddell Sea | 68 |  | 2014 | Polarstern ANT-XXIX/9 | PS82_151-1 |  | -74.540556 | -28.530556 | 1750 |
| Ophionotus | victoriae | 1.152.3 | WAMZ88599 | Weddell Sea | 68 |  | 2014 | Polarstern ANT-XXIX/9 | PS82_115-1 |  | -77.611333 | -38.938833 | 1058 |
| Ophionotus | victoriae | DSOPH3096 |  | Weddell Sea | 78 | KY048246 | 2011 | Polarstern ANT-XXVII/3 | PS77_291-1 |  | -70.842 | -10.587 | 268 |
| Ophionotus | victoriae | DSOPH3311 |  | Weddell Sea | 78 | KY048246 | 2011 | Polarstern ANT-XXVII/3 | PS77_275-3 |  | -70.934 | -10.496 | 238 |
| Ophionotus | victoriae | 274-3.14 | WAMZ88571 | Weddell Sea | 78 |  | 2011 | Polarstern ANT-XXVII/3 | PS77_274-3 |  | -70.949167 | -10.574167 | 333 |
| Ophionotus | victoriae | 308-1.8 | WAMZ88583 | Weddell Sea | 78 |  | 2011 | Polarstern ANT-XXVII/3 | PS77_308-1 |  | -70.858333 | -10.593056 | 250 |
| Ophionotus | victoriae | DSOPH3097 |  | Weddell Sea | 79 | KY048247 | 2011 | Polarstern ANT-XXVII/3 | PS77_291-1 |  | -70.842 | -10.587 | 268 |
| Ophionotus | victoriae | 291-1.3 | WAMZ88579 | Weddell Sea | 79 |  | 2011 | Polarstern ANT-XXVII/3 | PS77_291-1 |  | -70.847222 | -10.59 | 284 |
| Ophionotus | victoriae | 1.164.3 | WAMZ88593 | Weddell Sea | 160 |  | 2014 | Polarstern ANT-XXIX/9 | PS82_151-1 |  | -74.540556 | -28.530556 | 1750 |
| Ophionotus | victoriae | 1.164.4 | WAMZ88594 | Weddell Sea | 161 |  | 2014 | Polarstern ANT-XXIX/9 | PS82_151-1 |  | -74.540556 | -28.530556 | 1750 |
| Ophionotus | victoriae | 1.152.2 | WAMZ88598 | Weddell Sea | 162 |  | 2014 | Polarstern ANT-XXIX/9 | PS82_115-1 |  | -77.611333 | -38.938833 | 1058 |
| Ophionotus | victoriae | 312.3C.01 |  | West Antarctic Peninsula | 1 | FJ917337 | 2006 | LMG-06-05 | 17 |  | -64.350361 | -61.759953 | 334 |
| Ophionotus | victoriae | 312.3C.03 |  | West Antarctic Peninsula | 1 | FJ917337 | 2006 | LMG-06-05 | 17 |  | -64.350361 | -61.759953 | 334 |
| Ophionotus | victoriae | 312.3C.07 |  | West Antarctic Peninsula | 1 | FJ917339 | 2006 | LMG-06-05 | 17 |  | -64.350361 | -61.759953 | 334 |
| Ophionotus | victoriae | 312.3C.09 |  | West Antarctic Peninsula | 1 | FJ917339 | 2006 | LMG-06-05 | 17 |  | -64.350361 | -61.759953 | 334 |
| Ophionotus | victoriae | 312.3C.16 |  | West Antarctic Peninsula | 1 | FJ917337 | 2006 | LMG-06-05 | 17 |  | -64.350361 | -61.759953 | 334 |
| Ophionotus | victoriae | 398.1E.12 |  | West Antarctic Peninsula | 1 | FJ917337 | 2006 | LMG-06-05 | 47 |  | -67.717028 | -68.243286 | 170 |
| Ophionotus | victoriae | 398.1E.13 |  | West Antarctic Peninsula | 1 | FJ917345 | 2006 | LMG-06-05 | 47 |  | -67.717028 | -68.243286 | 170 |
| Ophionotus | victoriae | 422.1C.01 |  | West Antarctic Peninsula | 1 | FJ917339 | 2006 | LMG-06-05 | 58 |  | -65.183694 | -64.243283 | 285 |
| Ophionotus | victoriae | 422.1C.03 |  | West Antarctic Peninsula | 1 | FJ917337 | 2006 | LMG-06-05 | 58 |  | -65.183694 | -64.243283 | 285 |
| Ophionotus | victoriae | 422.1C.04 |  | West Antarctic Peninsula | 1 | FJ917339 | 2006 | LMG-06-05 | 58 |  | -65.183694 | -64.243283 | 285 |
| Ophionotus | victoriae | 422.1C.05 |  | West Antarctic Peninsula | 1 | FJ917339 | 2006 | LMG-06-05 | 58 |  | -65.183694 | -64.243283 | 285 |
| Ophionotus | victoriae | 422.1C.06 |  | West Antarctic Peninsula | 1 | FJ917337 | 2006 | LMG-06-05 | 58 |  | -65.183694 | -64.243283 | 285 |
| Ophionotus | victoriae | 422.1C.08 |  | West Antarctic Peninsula | 1 | FJ917339 | 2006 | LMG-06-05 | 58 |  | -65.183694 | -64.243283 | 285 |
| Ophionotus | victoriae | 422.1C.14 |  | West Antarctic Peninsula | 1 | FJ917339 | 2006 | LMG-06-05 | 58 |  | -65.183694 | -64.243283 | 285 |
| Ophionotus | victoriae | DSOPH446 |  | West Antarctic Peninsula | 1 | FJ917337 | 2009 | JR230 | AGT-2B |  | -67.983 | -68.438 | 586 |
| Ophionotus | victoriae | Op1042_3E_1 |  | West Antarctic Peninsula | 1 | FJ917337 | 2013 | LMG-13-12 | 26 |  | -64.846183 | -62.959483 | 301 |
| Ophionotus | victoriae | Op1042_3E_2 |  | West Antarctic Peninsula | 1 | FJ917339 | 2013 | LMG-13-12 | 26 |  | -64.846183 | -62.959483 | 301 |
| Ophionotus | victoriae | Op1042_3E_3 |  | West Antarctic Peninsula | 1 | FJ917339 | 2013 | LMG-13-12 | 26 |  | -64.846183 | -62.959483 | 301 |
| Ophionotus | victoriae | Op1042_3E_4 |  | West Antarctic Peninsula | 1 | FJ917339 | 2013 | LMG-13-12 | 26 |  | -64.846183 | -62.959483 | 301 |
| Ophionotus | victoriae | Op1042_3E_6 |  | West Antarctic Peninsula | 1 | FJ917337 | 2013 | LMG-13-12 | 26 |  | -64.846183 | -62.959483 | 301 |
| Ophionotus | victoriae | Op1042_3E_9 |  | West Antarctic Peninsula | 1 | FJ917339 | 2013 | LMG-13-12 | 26 |  | -64.846183 | -62.959483 | 301 |
| Ophionotus | victoriae | Op867_4E_10 |  | West Antarctic Peninsula | 1 | FJ917339 | 2013 | LMG-13-12 | 3 |  | -63.805542 | -60.479083 | 428 |
| Ophionotus | victoriae | Op867_4E_2 |  | West Antarctic Peninsula | 1 | FJ917339 | 2013 | LMG-13-12 | 3 |  | -63.805542 | -60.479083 | 428 |
| Ophionotus | victoriae | Op867_4E_3 |  | West Antarctic Peninsula | 1 | FJ917339 | 2013 | LMG-13-12 | 3 |  | -63.805542 | -60.479083 | 428 |
| Ophionotus | victoriae | Op867_4E_5 |  | West Antarctic Peninsula | 1 | FJ917337 | 2013 | LMG-13-12 | 3 |  | -63.805542 | -60.479083 | 428 |
| Ophionotus | victoriae | Op867_4E_6 |  | West Antarctic Peninsula | 1 | FJ917339 | 2013 | LMG-13-12 | 3 |  | -63.805542 | -60.479083 | 428 |
| Ophionotus | victoriae | Op867_4E_7 |  | West Antarctic Peninsula | 1 | FJ917337 | 2013 | LMG-13-12 | 3 |  | -63.805542 | -60.479083 | 428 |
| Ophionotus | victoriae | Op867_4E_8 |  | West Antarctic Peninsula | 1 | FJ917339 | 2013 | LMG-13-12 | 3 |  | -63.805542 | -60.479083 | 428 |
| Ophionotus | victoriae | Op867_4E_9 |  | West Antarctic Peninsula | 1 | FJ917339 | 2013 | LMG-13-12 | 3 |  | -63.805542 | -60.479083 | 428 |
| Ophionotus | victoriae | 312.3C.05 |  | West Antarctic Peninsula | 3 | FJ917338 | 2006 | LMG-06-05 | 17 |  | -64.350361 | -61.759953 | 334 |
| Ophionotus | victoriae | 312.3C.15 |  | West Antarctic Peninsula | 3 | FJ917340 | 2006 | LMG-06-05 | 17 |  | -64.350361 | -61.759953 | 334 |
| Ophionotus | victoriae | Op1042_3E_5 |  | West Antarctic Peninsula | 3 | FJ917340 | 2013 | LMG-13-12 | 26 |  | -64.846183 | -62.959483 | 301 |
| Ophionotus | victoriae | Op867_4E_1 |  | West Antarctic Peninsula | 3 | FJ917340 | 2013 | LMG-13-12 | 3 |  | -63.805542 | -60.479083 | 428 |
| Ophionotus | victoriae | 362.1C.02 |  | West Antarctic Peninsula | 6 | FJ917309 | 2006 | LMG-06-05 | 33 |  | -67.733694 | -69.293283 | 122 |
| Ophionotus | victoriae | 362.1C.03 |  | West Antarctic Peninsula | 6 | FJ917309 | 2006 | LMG-06-05 | 33 |  | -67.733694 | -69.293283 | 122 |
| Ophionotus | victoriae | 362.1C.04 |  | West Antarctic Peninsula | 6 | FJ917309 | 2006 | LMG-06-05 | 33 |  | -67.733694 | -69.293283 | 122 |
| Ophionotus | victoriae | 362.1C.05 |  | West Antarctic Peninsula | 6 | FJ917309 | 2006 | LMG-06-05 | 33 |  | -67.733694 | -69.293283 | 122 |
| Ophionotus | victoriae | 362.1C.11 |  | West Antarctic Peninsula | 6 | FJ917344 | 2006 | LMG-06-05 | 33 |  | -67.733694 | -69.293283 | 122 |
| Ophionotus | victoriae | DSOPH1263 |  | West Antarctic Peninsula | 6 | FJ917309 | 2009 | JR230 | AGT-21A |  | -67.546 | -70.189 | 508 |
| Ophionotus | victoriae | 312.3C.02 |  | West Antarctic Peninsula | 7 | FJ917322 | 2006 | LMG-06-05 | 17 |  | -64.350361 | -61.759953 | 334 |
| Ophionotus | victoriae | 398.1E.07 |  | West Antarctic Peninsula | 7 | FJ917322 | 2006 | LMG-06-05 | 47 |  | -67.717028 | -68.243286 | 170 |
| Ophionotus | victoriae | 362.1C.09 |  | West Antarctic Peninsula | 8 | FJ917316 | 2006 | LMG-06-05 | 33 |  | -67.733694 | -69.293283 | 122 |
| Ophionotus | victoriae | 362.1C.12 |  | West Antarctic Peninsula | 8 | FJ917316 | 2006 | LMG-06-05 | 33 |  | -67.733694 | -69.293283 | 122 |
| Ophionotus | victoriae | 398.1E.02 |  | West Antarctic Peninsula | 8 | FJ917316 | 2006 | LMG-06-05 | 47 |  | -67.717028 | -68.243286 | 170 |
| Ophionotus | victoriae | 398.1E.14 |  | West Antarctic Peninsula | 8 | FJ917316 | 2006 | LMG-06-05 | 47 |  | -67.717028 | -68.243286 | 170 |
| Ophionotus | victoriae | 362.1C.01 |  | West Antarctic Peninsula | 39 | FJ917342 | 2006 | LMG-06-05 | 33 |  | -67.733694 | -69.293283 | 122 |
| Ophionotus | victoriae | 362.1C.10 |  | West Antarctic Peninsula | 39 | FJ917342 | 2006 | LMG-06-05 | 33 |  | -67.733694 | -69.293283 | 122 |
| Ophionotus | victoriae | 362.1C.07 |  | West Antarctic Peninsula | 40 | FJ917343 | 2006 | LMG-06-05 | 33 |  | -67.733694 | -69.293283 | 122 |
| Ophionotus | victoriae | 398.1E.01 |  | West Antarctic Peninsula | 40 | FJ917343 | 2006 | LMG-06-05 | 47 |  | -67.717028 | -68.243286 | 170 |
| Ophionotus | victoriae | 398.1E.15 |  | West Antarctic Peninsula | 40 | FJ917343 | 2006 | LMG-06-05 | 47 |  | -67.717028 | -68.243286 | 170 |
| Ophionotus | victoriae | 422.1C.02 |  | West Antarctic Peninsula | 41 | FJ917346 | 2006 | LMG-06-05 | 58 |  | -65.183694 | -64.243283 | 285 |
| Ophionotus | victoriae | 422.1C.07 |  | West Antarctic Peninsula | 42 | FJ917347 | 2006 | LMG-06-05 | 58 |  | -65.183694 | -64.243283 | 285 |
| Ophionotus | victoriae | 422.1C.10 |  | West Antarctic Peninsula | 43 | FJ917348 | 2006 | LMG-06-05 | 58 |  | -65.183694 | -64.243283 | 285 |
| Ophionotus | victoriae | Op1042_3E_7 |  | West Antarctic Peninsula | 83 | KY048252 | 2013 | LMG-13-12 | 26 |  | -64.846183 | -62.959483 | 301 |
| Ophionotus | victoriae | Op1042_3E_10 |  | West Antarctic Peninsula | 86 | KY048256 | 2013 | LMG-13-12 | 26 |  | -64.846183 | -62.959483 | 301 |
| Ophionotus | victoriae | Op1042_3E_8 |  | West Antarctic Peninsula | 87 | KY048257 | 2013 | LMG-13-12 | 26 |  | -64.846183 | -62.959483 | 301 |
| Ophionotus | hexactis | SIOBICE5493A | SIOBICE5493 | Bransfield Strait | 11 |  | 2012 | Polarstern ANT-XXVIII/4 |  | 79279 | -62.278167 | -55.832667 | 302 |
| Ophionotus | hexactis | SIOBICE5493B | SIOBICE5493 | Bransfield Strait | 11 |  | 2012 | Polarstern ANT-XXVIII/4 |  | 79279 | -62.278167 | -55.832667 | 302 |
| Ophionotus | hexactis | SIOBICE5493C | SIOBICE5493 | Bransfield Strait | 11 |  | 2012 | Polarstern ANT-XXVIII/4 |  | 79279 | -62.278167 | -55.832667 | 302 |
| Ophionotus | hexactis | SIOBICE5493E | SIOBICE5493 | Bransfield Strait | 11 |  | 2012 | Polarstern ANT-XXVIII/4 |  | 79279 | -62.278167 | -55.832667 | 302 |
| Ophionotus | hexactis | SIOBICE5493G | SIOBICE5493 | Bransfield Strait | 11 |  | 2012 | Polarstern ANT-XXVIII/4 |  | 79279 | -62.278167 | -55.832667 | 302 |
| Ophionotus | hexactis | SIOBICE5493H | SIOBICE5493 | Bransfield Strait | 11 |  | 2012 | Polarstern ANT-XXVIII/4 |  | 79279 | -62.278167 | -55.832667 | 302 |
| Ophionotus | hexactis | SIOBICE5493I | SIOBICE5493 | Bransfield Strait | 11 |  | 2012 | Polarstern ANT-XXVIII/4 |  | 79279 | -62.278167 | -55.832667 | 302 |
| Ophionotus | hexactis | SIOBICE5493J | SIOBICE5493 | Bransfield Strait | 11 |  | 2012 | Polarstern ANT-XXVIII/4 |  | 79279 | -62.278167 | -55.832667 | 302 |
| Ophionotus | hexactis | SIOBICE5493K | SIOBICE5493 | Bransfield Strait | 11 |  | 2012 | Polarstern ANT-XXVIII/4 |  | 79279 | -62.278167 | -55.832667 | 302 |
| Ophionotus | hexactis | SIOBICE5493L | SIOBICE5493 | Bransfield Strait | 11 |  | 2012 | Polarstern ANT-XXVIII/4 |  | 79279 | -62.278167 | -55.832667 | 302 |
| Ophionotus | hexactis | SIOBICE5493D | SIOBICE5493 | Bransfield Strait | 12 |  | 2012 | Polarstern ANT-XXVIII/4 |  | 79279 | -62.278167 | -55.832667 | 302 |
| Ophionotus | hexactis | SIOBICE5493F | SIOBICE5493 | Bransfield Strait | 13 |  | 2012 | Polarstern ANT-XXVIII/4 |  | 79279 | -62.278167 | -55.832667 | 302 |
| Ophionotus | hexactis | WAMZ43230 | WAMZ43230 | Heard Island | 111 |  | 2017 | ACE 2016/17 | 18 | 279 | -52.3554 | 74.801 | 203 |
| Ophionotus | hexactis | WAMZ43231 | WAMZ43231 | Heard Island | 111 |  | 2017 | ACE 2016/17 | 18 | 279 | -52.3554 | 74.801 | 203 |
| Ophionotus | hexactis | WAMZ43232 | WAMZ43232 | Heard Island | 111 |  | 2017 | ACE 2016/17 | 18 | 279 | -52.3554 | 74.801 | 203 |
| Ophionotus | hexactis | WAMZ43233 | WAMZ43233 | Heard Island | 111 |  | 2017 | ACE 2016/17 | 18 | 279 | -52.3554 | 74.801 | 203 |
| Ophionotus | hexactis | WAMZ43234 | WAMZ43234 | Heard Island | 111 |  | 2017 | ACE 2016/17 | 18 | 279 | -52.3554 | 74.801 | 203 |
| Ophionotus | hexactis | WAMZ43235 | WAMZ43235 | Heard Island | 111 |  | 2017 | ACE 2016/17 | 18 | 279 | -52.3554 | 74.801 | 203 |
| Ophionotus | hexactis | WAMZ43236 | WAMZ43236 | Heard Island | 111 |  | 2017 | ACE 2016/17 | 18 | 279 | -52.3554 | 74.801 | 203 |
| Ophionotus | hexactis | WAMZ43237 | WAMZ43237 | Heard Island | 111 |  | 2017 | ACE 2016/17 | 18 | 279 | -52.3554 | 74.801 | 203 |
| Ophionotus | hexactis | WAMZ43238 | WAMZ43238 | Heard Island | 111 |  | 2017 | ACE 2016/17 | 18 | 279 | -52.3554 | 74.801 | 203 |
| Ophionotus | hexactis | WAMZ43239 | WAMZ43239 | Heard Island | 144 |  | 2017 | ACE 2016/17 | 18 | 279 | -52.3554 | 74.801 | 203 |
| Ophionotus | hexactis | PS133-4 |  | Larsen Ice Shelf | 48 | KU895454 | 2007 | Polarstern ANT-XIX/4 | PS61_113-4 |  | -65.3285 | -54.2417 | 1113 |
| Ophionotus | hexactis | SIOBICS3753 | SIOBICE5236 | Shag Rocks | 144 |  | 2011 | Scotia 2011 | SR1 | 4 | -53.45317 | -42.0579 | 174 |
| Ophionotus | hexactis | SIOBICS3755 | SIOBICE5225 | Shag Rocks | 144 |  | 2011 | Scotia 2011 | SR1 | 4 | -53.45317 | -42.0579 | 174 |
| Ophionotus | hexactis | SIOBICS3756 | SIOBICE5180 | Shag Rocks | 144 |  | 2011 | Scotia 2011 | SR1 | 4 | -53.45317 | -42.0579 | 174 |
| Ophionotus | hexactis | SIOBICS3757 | SIOBICE5230 | Shag Rocks | 144 |  | 2011 | Scotia 2011 | SR1 | 4 | -53.45317 | -42.0579 | 174 |
| Ophionotus | hexactis | SIOBICS3758 | SIOBICE5246 | Shag Rocks | 144 |  | 2011 | Scotia 2011 | SR1 | 4 | -53.45317 | -42.0579 | 174 |
| Ophionotus | hexactis | SIOBICS3759 | SIOBICE5283 | Shag Rocks | 144 |  | 2011 | Scotia 2011 | SR1 | 4 | -53.45317 | -42.0579 | 174 |
| Ophionotus | hexactis | SIOBICS3760 | SIOBICE5227 | Shag Rocks | 144 |  | 2011 | Scotia 2011 | SR1 | 4 | -53.45317 | -42.0579 | 174 |
| Ophionotus | hexactis | SIOBICS3761 | SIOBICE5184 | Shag Rocks | 144 |  | 2011 | Scotia 2011 | SR1 | 4 | -53.45317 | -42.0579 | 174 |
| Ophionotus | hexactis | SIOBICS3762 | SIOBICE5219 | Shag Rocks | 144 |  | 2011 | Scotia 2011 | SR1 | 4 | -53.45317 | -42.0579 | 174 |
| Ophionotus | hexactis | SIOBICS3754 | SIOBICE5251 | Shag Rocks | 151 |  | 2011 | Scotia 2011 | SR1 | 4 | -53.45317 | -42.0579 | 174 |
| Ophionotus | hexactis | SIOBICS0036A | SIOBICE4798 | South Georgia | 144 |  | 2011 | Scotia 2011 | SG3 | 21 | -55.05153 | -35.3955 | 119 |
| Ophionotus | hexactis | SIOBICS0036C | SIOBICE4798 | South Georgia | 144 |  | 2011 | Scotia 2011 | SG3 | 21 | -55.05153 | -35.3955 | 119 |
| Ophionotus | hexactis | SIOBICS0036D | SIOBICE4798 | South Georgia | 144 |  | 2011 | Scotia 2011 | SG3 | 21 | -55.05153 | -35.3955 | 119 |
| Ophionotus | hexactis | SIOBICS0036E | SIOBICE4798 | South Georgia | 144 |  | 2011 | Scotia 2011 | SG3 | 21 | -55.05153 | -35.3955 | 119 |
| Ophionotus | hexactis | SIOBICS0036F | SIOBICE4798 | South Georgia | 144 |  | 2011 | Scotia 2011 | SG3 | 21 | -55.05153 | -35.3955 | 119 |
| Ophionotus | hexactis | SIOBICS0036H | SIOBICE4798 | South Georgia | 144 |  | 2011 | Scotia 2011 | SG3 | 21 | -55.05153 | -35.3955 | 119 |
| Ophionotus | hexactis | SIOBICS0036I | SIOBICE4798 | South Georgia | 144 |  | 2011 | Scotia 2011 | SG3 | 21 | -55.05153 | -35.3955 | 119 |
| Ophionotus | hexactis | SIOBICS0036J | SIOBICE4798 | South Georgia | 144 |  | 2011 | Scotia 2011 | SG3 | 21 | -55.05153 | -35.3955 | 119 |
| Ophionotus | hexactis | SIOBICS0037A | SIOBICE4780 | South Georgia | 144 |  | 2011 | Scotia 2011 | SG3 | 21 | -55.05153 | -35.3955 | 119 |
| Ophionotus | hexactis | SIOBICS0037B | SIOBICE4780 | South Georgia | 144 |  | 2011 | Scotia 2011 | SG3 | 21 | -55.05153 | -35.3955 | 119 |
| Ophionotus | hexactis | SIOBICS0037D | SIOBICE4780 | South Georgia | 144 |  | 2011 | Scotia 2011 | SG3 | 21 | -55.05153 | -35.3955 | 119 |
| Ophionotus | hexactis | SIOBICS0037H | SIOBICE4780 | South Georgia | 144 |  | 2011 | Scotia 2011 | SG3 | 21 | -55.05153 | -35.3955 | 119 |
| Ophionotus | hexactis | SIOBICS0037I | SIOBICE4780 | South Georgia | 144 |  | 2011 | Scotia 2011 | SG3 | 21 | -55.05153 | -35.3955 | 119 |
| Ophionotus | hexactis | SIOBICS0037J | SIOBICE4780 | South Georgia | 144 |  | 2011 | Scotia 2011 | SG3 | 21 | -55.05153 | -35.3955 | 119 |
| Ophionotus | hexactis | SIOBICS3354B | SIOBICE4774 | South Georgia | 144 |  | 2011 | Scotia 2011 | SG2a | 18 | -53.80054 | -37.21886 | 145 |
| Ophionotus | hexactis | SIOBICS3354C | SIOBICE4774 | South Georgia | 144 |  | 2011 | Scotia 2011 | SG2a | 18 | -53.80054 | -37.21886 | 145 |
| Ophionotus | hexactis | SIOBICS3354D | SIOBICE4774 | South Georgia | 144 |  | 2011 | Scotia 2011 | SG2a | 18 | -53.80054 | -37.21886 | 145 |
| Ophionotus | hexactis | SIOBICS3354E | SIOBICE4774 | South Georgia | 144 |  | 2011 | Scotia 2011 | SG2a | 18 | -53.80054 | -37.21886 | 145 |
| Ophionotus | hexactis | SIOBICS3354I | SIOBICE4774 | South Georgia | 144 |  | 2011 | Scotia 2011 | SG2a | 18 | -53.80054 | -37.21886 | 145 |
| Ophionotus | hexactis | SIOBICS3355A | SIOBICE4782 | South Georgia | 144 |  | 2011 | Scotia 2011 | SG2a | 18 | -53.80054 | -37.21886 | 145 |
| Ophionotus | hexactis | SIOBICS3355C | SIOBICE4782 | South Georgia | 144 |  | 2011 | Scotia 2011 | SG2a | 18 | -53.80054 | -37.21886 | 145 |
| Ophionotus | hexactis | SIOBICS3355D | SIOBICE4782 | South Georgia | 144 |  | 2011 | Scotia 2011 | SG2a | 18 | -53.80054 | -37.21886 | 145 |
| Ophionotus | hexactis | SIOBICS3355E | SIOBICE4782 | South Georgia | 144 |  | 2011 | Scotia 2011 | SG2a | 18 | -53.80054 | -37.21886 | 145 |
| Ophionotus | hexactis | SIOBICS3355F | SIOBICE4782 | South Georgia | 144 |  | 2011 | Scotia 2011 | SG2a | 18 | -53.80054 | -37.21886 | 145 |
| Ophionotus | hexactis | SIOBICS3355G | SIOBICE4782 | South Georgia | 144 |  | 2011 | Scotia 2011 | SG2a | 18 | -53.80054 | -37.21886 | 145 |
| Ophionotus | hexactis | SIOBICS3355I | SIOBICE4782 | South Georgia | 144 |  | 2011 | Scotia 2011 | SG2a | 18 | -53.80054 | -37.21886 | 145 |
| Ophionotus | hexactis | SIOBICS3355J | SIOBICE4782 | South Georgia | 144 |  | 2011 | Scotia 2011 | SG2a | 18 | -53.80054 | -37.21886 | 145 |
| Ophionotus | hexactis | SIOBICS0036B | SIOBICE4798 | South Georgia | 145 |  | 2011 | Scotia 2011 | SG3 | 21 | -55.05153 | -35.3955 | 119 |
| Ophionotus | hexactis | SIOBICS0036G | SIOBICE4798 | South Georgia | 145 |  | 2011 | Scotia 2011 | SG3 | 21 | -55.05153 | -35.3955 | 119 |
| Ophionotus | hexactis | SIOBICS0037E | SIOBICE4780 | South Georgia | 145 |  | 2011 | Scotia 2011 | SG3 | 21 | -55.05153 | -35.3955 | 119 |
| Ophionotus | hexactis | SIOBICS0037F | SIOBICE4780 | South Georgia | 145 |  | 2011 | Scotia 2011 | SG3 | 21 | -55.05153 | -35.3955 | 119 |
| Ophionotus | hexactis | SIOBICS0037C | SIOBICE4780 | South Georgia | 146 |  | 2011 | Scotia 2011 | SG3 | 21 | -55.05153 | -35.3955 | 119 |
| Ophionotus | hexactis | SIOBICS0037G | SIOBICE4780 | South Georgia | 147 |  | 2011 | Scotia 2011 | SG3 | 21 | -55.05153 | -35.3955 | 119 |
| Ophionotus | hexactis | SIOBICS3354J | SIOBICE4774 | South Georgia | 147 |  | 2011 | Scotia 2011 | SG2a | 18 | -53.80054 | -37.21886 | 145 |
| Ophionotus | hexactis | SIOBICS3355B | SIOBICE4782 | South Georgia | 147 |  | 2011 | Scotia 2011 | SG2a | 18 | -53.80054 | -37.21886 | 145 |
| Ophionotus | hexactis | SIOBICS3354A | SIOBICE4774 | South Georgia | 148 |  | 2011 | Scotia 2011 | SG2a | 18 | -53.80054 | -37.21886 | 145 |
| Ophionotus | hexactis | SIOBICS3354F | SIOBICE4774 | South Georgia | 149 |  | 2011 | Scotia 2011 | SG2a | 18 | -53.80054 | -37.21886 | 145 |
| Ophionotus | hexactis | SIOBICS3354G | SIOBICE4774 | South Georgia | 149 |  | 2011 | Scotia 2011 | SG2a | 18 | -53.80054 | -37.21886 | 145 |
| Ophionotus | hexactis | SIOBICS3355H | SIOBICE4782 | South Georgia | 149 |  | 2011 | Scotia 2011 | SG2a | 18 | -53.80054 | -37.21886 | 145 |
| Ophionotus | hexactis | SIOBICS3354H | SIOBICE4774 | South Georgia | 150 |  | 2011 | Scotia 2011 | SG2a | 18 | -53.80054 | -37.21886 | 145 |

**Appendix S2.** (a, b) *Ophionotus victoriae* from South Georgia, SIO-BIC E6408 (field number S20231) and (c, d) *O. victoriae* from South Georgia, SIO-BIC 6420 (field number S20237). (e) *O. hexactis* from Bransfield Strait (SIO-BIC E5493A-E5493L). Note specimen from the top right corner of this photo was not sequenced in this study. (f) *O. hexactis* from Heard Island, Kerguelen Plateau (WAMZ43230-WAM43239). Note 10 out of 17 specimens were sequenced from this lot (lot number WAMZ44197) in this study.

**Appendix S3.**

**Methods**

Phylogenetic analyses and molecular species delimitation

A maximum likelihood (ML) estimate and a Bayesian inference (BI) of phylogeny were reconstructed to determine relationships between *O. victoriae* and *O. hexactis*, as well as input for tree-based species delineation analyses. For ML tree-based species delimitation analyses, sequences were first collapsed into unique haplotypes for easier presentation using FaBox v1.5 (Villesen 2007) (see Appendix S1 for haplotype information). No sequences were collapsed into unique haplotypes for BI tree-based species delimitation analyses. Sequences were not collapsed into unique haplotypes for BI tree is due to the fact that, in genealogy-based approach such as BEAST, identical sequences are treated as different alleles coalescing back to the most common recent ancestor (Talavera et al. 2013). During this process, non-zero branch lengths will be inserted between identical sequences, which would have an effect on the overall branch length for each group (Talavera et al. 2013). Whether the sequences are collapsed or not, however, does not seem have an effect on the BI-tree based species delimitation analysis used in this study (Talavera et al. 2013). The COI sequences of *Ophiura aequalis*, *O. micracantha*, *Ophiocten ludwigi* and *Ophiocrossota multispina* were included as outgroups in order to root the tree (Hugall et al. 2016) (GenBank accession numbers: KU894989, KU894990, KU895450 and KU895449, respectively). The ML tree was generated using the IQ-TREE web server v1.6.11 (Trifinopoulos et al. 2016) using ultrafast bootstrap support of 1000 iterations for node support. A substitution model of the *Ophionotus* spp. sequences (TN+F+I+G4) was determined based on Bayesian Information Criterion (BIC) using ModelFinder on the IQ-TREE web server (Kalyaanamoorthy et al. 2017). The Bayesian inference (BI) phylogenetic tree was generated using all COI sequences in BEAST under the substitution model of TN+F+I+G4, uncorrelated lognormal relaxed clock and using a constant coalescent constant population tree prior (Michonneau 2016). A Markov Chain Monte Carlo (MCMC) analysis was run for 200 million generations sampled at every 5,000 generations. Tracer used to inspect convergence values based on based on trace plots and ESS > 200. The final 20,000 trees were kept using Tree Annotator v1.8.4 (from the BEAST pacakge). The final ML and BI consensus tree were visualised in FigTree v1.4.3 (Rambaut 2016).

To assess species limits within *O. victoriae*, as well as between *O. victoriae* and *O. hexactis*, four single-locus species delineation methods were used: ABGD algorithm (Automated Barcode Gap Discovery) (Puillandre et al. 2012), mPTP (multi-rate Poisson Tree Processes) (Kapli et al. 2017), bPTP (Bayesian implementation of PTP mode) (Zhang et al. 2013) and sGMYC (single-threshold General Mixed Yule Coalescent) (Fujisawa and Barraclough 2013). ABGD was performed using the online web server (https://bioinfo.mnhn.fr/abi/public/abgd/abgdweb.html) based on genetic p-distance between haplotypes with a priori thresholds (P) set between 0.001 and 0.1. Both mPTP and bPTP were performed using online web servers (https://mptp.h-its.org and https://species.h-its.org/). For both PTP analyses, a rooted ML tree was used as input, with outgroup taxa excluded prior to species delimitation. During the search in bPTP, 500,000 MCMC generations were used, with a thinning parameter of 100 and the first 10% discarded as burn-in. For sGMYC, an unrooted BI tree with outgroup taxa excluded was used as input and sGMYC was performed using the R packages ‘SPLITS’ (Ezard et al. 2009), ‘APE’ (Paradis et al. 2004), ‘PARAN’ (Dinno 2012) and ‘RNCL’ (Michonneau et al. 2016).

**Results**

Phylogenetic trees and species delimitation

The topology of ML and BI trees revealed that both *O. victoriae* and *O. hexactis* are paraphyletic species, with both trees showing *O. victoriae* plus *O. hexactis* sequences comprising a single lineage together (Appendix S9 and Appendix S10). The ML tree showed short internal branches for the clade containing *O. victoriae* haplotypes, with *O. hexactis* haplotypes nested within *O. victoriae* haplotypes (SH-aLRT support = 98.7%, Ultrafast BS = 99%) (Appendix S9). In the ML tree, *O. victoriae* COI sequences were also nested within clades of *O. hexactis*. The BI tree, also with short internal branches, suggested *O. victoriae* forms two separate clades (posterior probability = 100%) (Appendix S10). On the BI tree, *O. hexactis* is nested within one of *O. victoriae*’s clades alongside *O. victoriae* sequences (Appendix S10).

Species delimitation analyses by genetic distance (ABGD) suggests *O. victoriae* and *O. hexactis* are a single species with no barcoding gap between sampled haplotypes (Appendix S9). Tree-based species delineation analyses (mPTP, bPTP and sGMYC) indicate 1, 128 and 301 putative species, respectively, among *O. victoriae* and *O. hexactis*. However, no tree-based analyses distinguished *O. hexactis* and *O. victoriae* as monophyletic species; each delimitation analysis has defined at least one putative species comprised of haplotypes/or sequences from both species.

**References**

Dinno, A. (2012). paran: Horn's test of principal components/factors. https://cran.r‐project.org/web/packages/paran/index.html

Ezard, T., Fujisawa, T., & Barraclough, T. G. (2009). Splits: SPecies' LImits by threshold statistics. https://rdrr.io/rforge/splits/

Fujisawa, T., & Barraclough, T. G. (2013). Delimiting species using single‐locus data and the generalized mixed yule coalescent approach: A revised method and evaluation on simulated data sets. Systematic Biology, 62, 707–724. https://doi.org/10.1093/sysbio/syt033

Hugall, A. F., O'Hara, T. D., Hunjan, S., Nilsen, R., & Moussalli, A. (2016). An exon‐capture system for the entire class Ophiuroidea. Molecular Biology and Evolution, 33, 281–294. https://doi.org/10.1093/molbev/msv216

Kalyaanamoorthy, S., Minh, B. Q., Wong, T. K. F., Von Haeseler, A., & Jermiin, L. S. (2017). ModelFinder: Fast model selection for accurate phylogenetic estimates. Nature Methods, 14, 587–589.

Kapli, P., Lutteropp, S., Zhang, J., Kobert, K., Pavlidis, P., Stamatakis, A., & Flouri, T. Multi-rate Poisson tree processes for single-locus species delimitation under maximum likelihood and Markov chain Monte Carlo. Bioinformatics, 33, 1630–1638.

Michonneau, F. (2016). Using GMYC for species delineation. Zenodo. https://doi.org/10.5281/zenodo.838260

Michonneau, F., Bolker, B., Holder, M., Lewis, P., & O'Meara, B. (2016). rncl: An interface to the Nexus Class Library. https://cran.r‐project.org/package=rncl

Paradis, E., Claude, J., & Strimmer, K. (2004). APE: Analyses of phylogenetics and evolution in R language. Bioinformatics, 20, 289–290. https://doi.org/10.1093/bioinformatics/btg412

Pullandre, N., Lambert, A., Brouillet, S., & Achaz G. ABGD, Automatic Barcode Gap Discovery for primary species delimitation. Molecular Ecology, 21, 1864–1877.

Rambaut, A. (2016). FigTree v.1.4.3. http://tree.bio.ed.ac.uk/software/figtree/

Zhang, J., Kapli, P., Pavlidis, P., & Stamatakis, A. (2013). A general species delimitation method with applications to phylogenetic placements. Bioinformatics, 29, 2869–2876. https://doi.org/10.1093/bioinformatics/btt499

Talavera, G., Dincă, V., & Fila, R. (2013). Factors affecting species delimitations with the GMYC model: Insights from a butterfly survey. Methods in Ecology and Evolution, 4, 1101–1110. https://doi.org/10.1111/2041‐210X.12107

Trifinopoulos, J., Nguyen, L. T., von Haeseler, A., & Minh, B. Q. (2016). W‐IQ‐TREE: A fast online phylogenetic tool for maximum likelihood analysis. Nucleic Acids Research, 44, W232–W235. https://doi.org/10.1093/nar/gkw256

Villesen, P. (2007). FaBox: An online toolbox for FASTA sequences. Molecular Ecology Resources, 7, 965–968.

 **Appendix S4.** Raster surfaces of six environmental parameters used as the input spatial predictors of spatial genetic patterns of *Ophionotus victoriae* and *O. hexactis*. The raster surfaces representing each environmental parameter were either interpolated (a-d) or resampled (f) to achieve a cell resolution of 16 km that was pre-defined in (e).

**Appendix S5.** TCS haplotype network of *Ophionotus victoriae* and *O. hexactis* COI sequences (434 bp, n = 935), separated by species. Size and colours of circle represent the number of samples and sample locations associated with each haplotype. Black circle = inferred haplotype missing in the dataset. Hatch lines = inferred mutation steps between haplotypes.

**Appendix S6.** Analysis of molecular variable (AMOVA) between sample localities and species in *Ophionotus victoriae* and *O. hexactis*.

| Source of variation | Degrees of freedom | Sums of squares | Variance components | Percentage of variation |
| --- | --- | --- | --- | --- |
| Among species | 1 | 15.393 | 0.08441 *V_a_* | 15.86 |
| Among locations within species | 23 | 108.136 | 0.12071 *V_b_* | 22.67 |
| Within locations | 910 | 297.784 | 0.32724 *V_c_* | 61.47 |
| Total | 934 | 421.313 | 0.53235 |  |
| Fixation indices |  |  |  |  |
| F_ST_ | 0.26947 |  |  |  |
| F_SC_ | 0.38530 |  |  |  |
| F_CT_ | 0.15856 |  |  |  |
| Significant tests (1023 permutations) |  | | | |
| *V_c_* and F_ST_ | p <0.0000 ± 0.0000 | | | |
| *V_b_* and F_SC_ | p <0.0000 ± 0.0000 | | | |
| *V_a_* and F_CT_ | p = 0.00293 ± 0.00164 | | | |

**Appendix S7.** Pairwise F_ST_ between *Ophionotus victoriae* and *O. hexactis*. *significant value with p < 0.0001, with significant tests performed with 1023 permutations.

|  | *O. victoriae* | *O. hexactis* |
| --- | --- | --- |
| *O. hexactis* | 0 |  |
| *O. victoriae* | 0.20345* | 0 |
|  |  |  |

**Appendix S8.** Heatmap showing pairwise F_ST_ values estimated from COI data of *Ophionotus victoriae* and *O. hexactis*, between sample localities with sample size (n) > 5. Dots indicate significant difference after Bonferroni corrections (p < 0.00014).


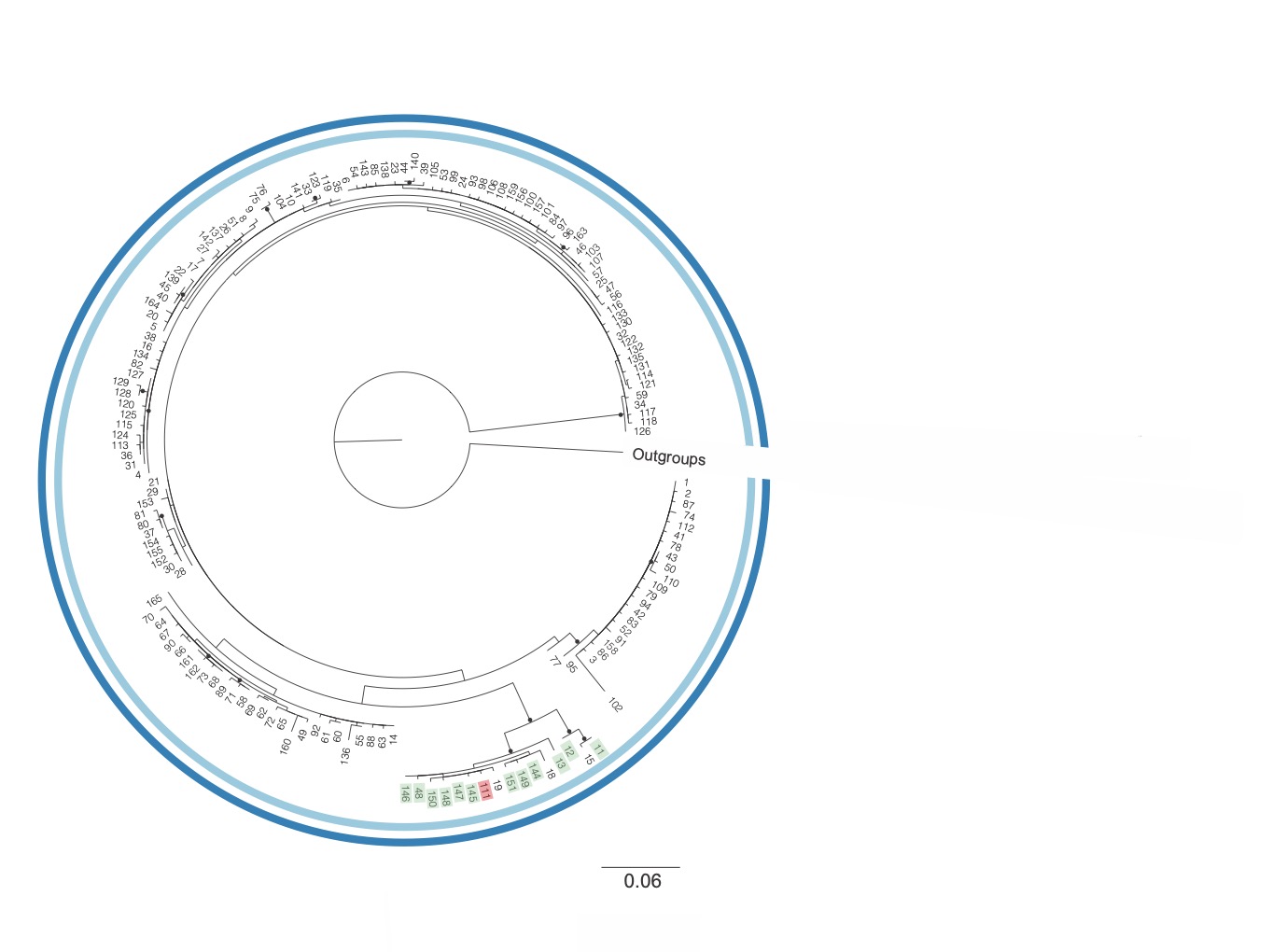


**Appendix S9.** Maximum likelihood tree of *Ophionotus victoriae* COI haplotypes (non-shaded) and *O. hexactis* (shaded in green). Haplotype ID shaded in red = shared haplotype between *O. victoriae* and *O. hexactis*. A solid circle at the node represents Shimodaira–Hasegawa approximate likelihood ratio test (SH-aLRT) >= 80% and ultrafast bootstrap (UFBoot) >= 95%. Bars indicate results proposed by species delimitation analyses: ABGD (light blue) and mPTP (dark blue). Results of bPTP and sGMYC not shown.


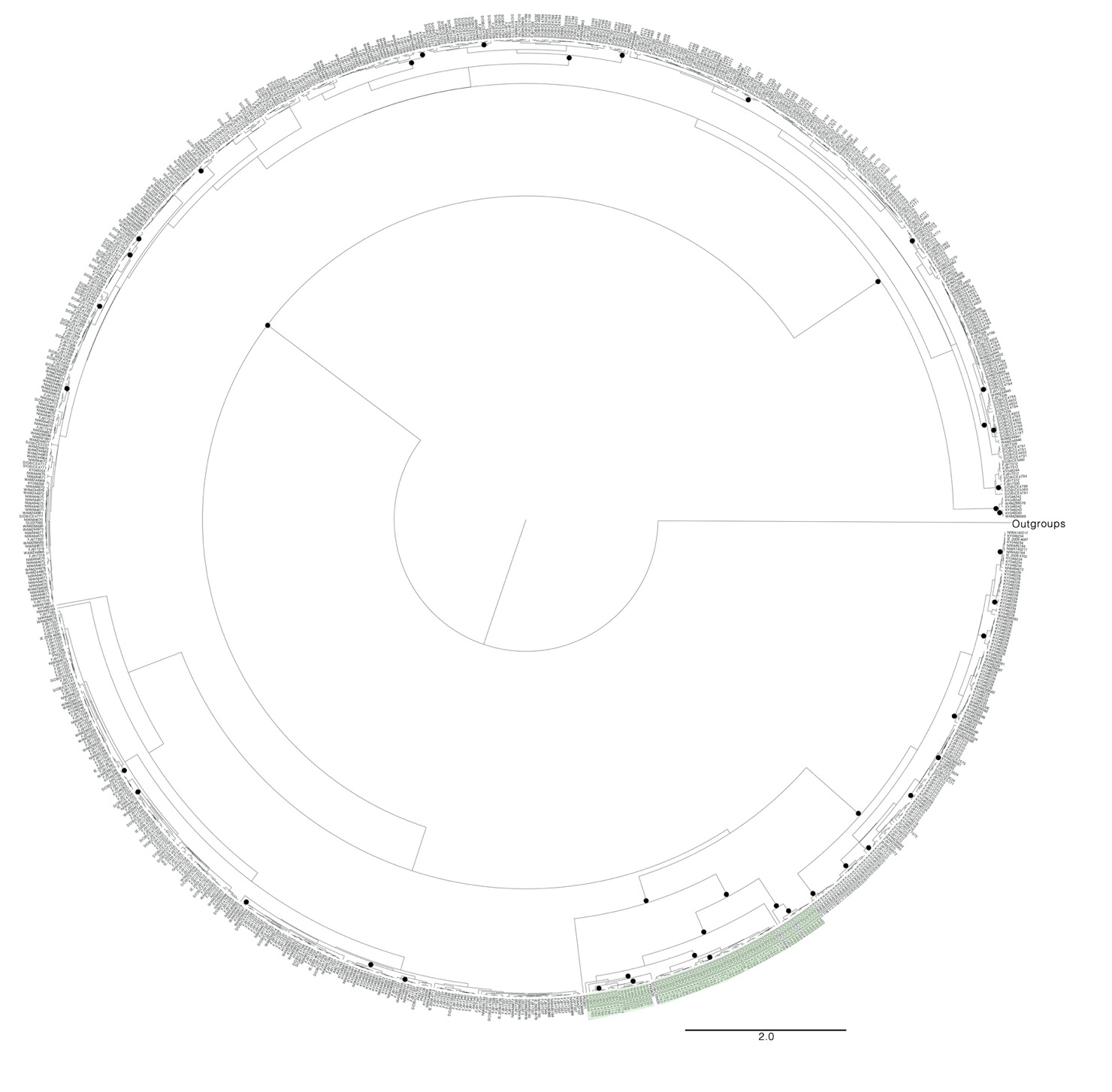


**Appendix S10.** Bayesian inference ultrametric tree of *Ophionotus victoriae* (non-shaded) and *O. hexactis* (shaded in green) COI sequences. A solid circle at the node represents posterior probability >= 90%.
